# Safety and Efficacy of Acetyl-DL-Leucine in Certain Types of Cerebellar Ataxia: The ALCAT Randomized Clinical Crossover Trial

**DOI:** 10.1001/jamanetworkopen.2021.35841

**Published:** 2021-12-14

**Authors:** Katharina Feil, Christine Adrion, Sylvia Boesch, Sarah Doss, Ilaria Giordano, Holger Hengel, Heike Jacobi, Thomas Klockgether, Thomas Klopstock, Wolfgang Nachbauer, Ludger Schöls, Katharina Marie Steiner, Claudia Stendel, Dagmar Timmann, Ivonne Naumann, Ulrich Mansmann, Michael Strupp, ALCAT Study Group

**Affiliations:** 1 Department of Neurology with Friedrich-Baur-Institute, Ludwig Maximilians University, University Hospital, Munich, Germany; 2 German Center for Vertigo and Balance Disorders (DSGZ), Ludwig Maximilians University, University Hospital, Campus Grosshadern, Munich, Germany; 3 Department of Neurology and Stroke, University Hospital Tübingen, Tübingen, Germany; 4 Institute for Medical Informatics, Biometry and Epidemiology (IBE), Ludwig Maximilians University, Munich, Germany; 5 Department of Neurology, Medical University Innsbruck, Innsbruck, Austria; 6 Department of Neurology and Center for Translational Neuro- and Behavioral Sciences, University Hospital Essen, Essen, Germany; 7 Department of Neurology, Charité – Universitätsmedizin Berlin, corporate member of Freie Universität Berlin, Humboldt-Universität zu Berlin, Berlin, Germany; 8 Department of Neurological Sciences, University of Nebraska Medical Center (UNMC), Omaha; 9 German Center for Neurodegenerative Diseases (DZNE), Center for Clinical Research, Bonn, Germany; 10 Department of Neurology and Hertie-Institute for Clinical Brain Research, University Hospital Tübingen, Tübingen, Germany; 11 Department of Neurology, University Hospital Heidelberg, Heidelberg, Germany; 12 Munich Cluster for Systems Neurology (SyNergy), Munich, Germany; 13 German Center for Neurodegenerative Diseases (DZNE), Munich, Germany; 14 German Center for Neurodegenerative Diseases (DZNE), Tübingen, Germany

## Abstract

**Importance:**

Cerebellar ataxia is a neurodegenerative disease impairing motor function characterized by ataxia of stance, gait, speech, and fine motor disturbances.

**Objective:**

To investigate the efficacy, safety, and tolerability of the modified essential amino acid acetyl-DL-leucine in treating patients who have cerebellar ataxia.

**Design, Setting, and Participants:**

The Acetyl-DL-leucine on Cerebellar Ataxia (ALCAT) trial was an investigator-initiated, multicenter, double-blind, randomized, placebo-controlled, clinical crossover trial. The study was conducted at 7 university hospitals in Germany and Austria between January 25, 2016, and February 17, 2017. Patients were aged at least 18 years and diagnosed with cerebellar ataxia of hereditary (suspected or genetically confirmed) or nonhereditary or unknown type presenting with a total score of at least 3 points on the Scale for the Assessment and Rating of Ataxia (SARA). Statistical analysis was performed from April 2018 to June 2018 and January 2020 to March 2020.

**Interventions:**

Patients were randomly assigned (1:1) to receive acetyl-DL-leucine orally (5 g per day after 2 weeks up-titration) followed by a matched placebo, each for 6 weeks, separated by a 4-week washout, or vice versa. The randomization was done via a web-based, permuted block-wise randomization list (block size, 2) that was stratified by disease subtype (hereditary vs nonhereditary or unknown) and site.

**Main Outcomes and Measures:**

Primary efficacy outcome was the absolute change of SARA total score from (period-dependent) baseline to week 6.

**Results:**

Among 108 patients who were randomly assigned to sequence groups (54 patients each), 55 (50.9%) were female; the mean (SD) age was 54.8 (14.4) years; and the mean (SD) SARA total score was 13.33 (5.57) points. The full analysis set included 105 patients (80 patients with hereditary, 25 with nonhereditary or unknown cerebellar ataxia). There was no evidence of a difference in the mean absolute change from baseline to week 6 in SARA total scores between both treatments (mean treatment difference: 0.23 points [95% CI, −0.40 to 0.85 points]).

**Conclusions and Relevance:**

In this large multicenter, double-blind, randomized, placebo-controlled clinical crossover trial, acetyl-DL-leucine in the investigated dosage and treatment duration was not superior to placebo for the symptomatic treatment of certain types of ataxia. The drug was well tolerated; and ALCAT yielded valuable information about the duration of treatment periods and the role of placebo response in cerebellar ataxia. These findings suggest that further symptom-oriented trials are needed for evaluating the long-term effects of acetyl-DL-leucine for well-defined subgroups of cerebellar ataxia.

**Trial Registration:**

EudraCT 2015-000460-34

## Key Points

**Question:**

Is the modified amino acid acetyl-DL-leucine effective and safe in patients with cerebellar ataxia of different etiologies?

**Findings:**

In this randomized clinical crossover trial including 108 patients with cerebellar ataxia, a 6-week treatment with acetyl-DL-leucine was not effective. Adverse events were mild.

**Meaning:**

This study’s findings suggest that further symptom-oriented trials evaluating the long-term effects of acetyl-DL-leucine for well-defined subgroups of cerebellar ataxia are needed.

## Teaser

This randomized clinical crossover trial investigates the efficacy and safety of the modified essential amino acid acetyl-DL-leucine in treating patients who have cerebellar ataxia.

## Introduction

Cerebellar ataxia is a frequent and disabling syndrome often caused by neurodegenerative cerebellar disorders.^1^ Clinical symptoms are disturbances of stance and gait, limb ataxia, fine motor deficits, slurred speech, as well as ocular motor disturbances.^2,3^ Most types of cerebellar ataxia are progressive.^4^ Possible effects of riluzole and varenicline have been described in certain subtypes of spinocerebellar ataxia (SCA) and of rovatirelin in a post-hoc, pooled subgroup analysis mainly in patients with SCA.^5,6,7,8^ In summary, no medication has convincingly shown efficacy for the symptomatic or causative treatment of degenerative cerebellar ataxia, and the treatment recommendation is physical therapy.^9,10^

Acetyl-DL-leucine is a modified, acetylated derivative of a natural essential amino acid and has been used for the symptomatic treatment of acute vertigo.^11^ In vitro animal studies showed effects of acetyl-DL-leucine on abnormally hyperpolarized and/or depolarized vestibular neurons by normalizing the membrane potential,^12^ as well as on clinical improvement of central compensation of postural symptoms in acute unilateral vestibulopathy, most likely by vestibulocerebellar activation with increased regional cerebral metabolic rate in the flocculus.^13^ Moreover, symptomatic and disease-modifying neuroprotective effects have been demonstrated in animal models of ataxias.^14^ Owing to phylogenetic and electrophysiological similarities between vestibular and cerebellar neurons, we hypothesized possible positive effects on cerebellar symptoms.^15,16^

Case series with different types of cerebellar ataxia revealed a clinical improvement of ataxia symptoms after short-term treatment with acetyl-DL-leucine in ataxia rating scores^17,18,19^ as well as in a reduced gait variability during slow walking in gait analysis.^18^ To fill the evidence gap of lacking double-blind randomized, placebo-controlled trials,^20^ the Acetyl-DL-leucine on Cerebellar Ataxia (ALCAT) trial was conducted to investigate the efficacy, safety, and tolerability of acetyl-DL-leucine for the symptomatic treatment in cerebellar ataxia.

## Methods

### Trial Design and Participants

ALCAT was an investigator-initiated, multicenter, double-blind, randomized, placebo-controlled, 2-treatment 2-period crossover phase 3 clinical trial at 7 university centers in Germany (Munich, Bonn, Essen, Tübingen, Berlin) and Austria (Innsbruck). Recruitment occurred between January 25, 2016, and February 17, 2017. The trial protocol (Supplement 1) was reported before enrollment was completed.^21^ ALCAT followed the Consolidated Standards of Reporting Trials (CONSORT) reporting guideline for randomized clinical trials. Eligible patients were aged at least 18 years, diagnosed with cerebellar ataxia of hereditary (suspected or genetically confirmed) or nonhereditary or unknown type presenting with a Scale for the Assessment and Rating of Ataxia (SARA) total score of at least 3 points. Key exclusion criteria included ataxia due to clinically likely multisystem atrophy type C (MSA-C), Friedreich ataxia, and rapid progression of ataxia (eTable 1 in Supplement 2).

Ethical approval of ALCAT was granted for each of the participating centers prior to patient recruitment (leading ethics committee: ethics committee of the Medical Faculty of the LMU Munich, Germany; ethics committee in Innsbruck, Austria), and by Germany’s Federal Institute for Drugs and Medical Devices. Clinical trial authorization was granted on November 18, 2015. All participants provided written informed consent before any study procedures or assessments were performed.

### Randomization and Masking

Patients were randomized in a 1:1 ratio using an internet-based, password-protected randomization tool. Randomization technique was based on random blocks of size 2 considering stratification by study site and hereditary vs nonhereditary or unknown cerebellar ataxia. All patients, investigators, and assessors were masked to treatment allocation (Supplement 1).

### Procedures

Patients were initially screened and assessed for eligibility at the first visit and randomized at the second to 1 of 2 treatment sequences (active treatment followed by placebo [denoted as A-P] or vice versa [P-A]). Each treatment sequence consisted of two 6-week treatment periods (42 days) divided by a 4-week washout (28 days). A total of 8 study visits were scheduled (eFigure 1 in Supplement 2). Participants received study medication at the beginning of each treatment period and were instructed to apply a 2-week uptitration scheme (initial dosage of 1.5 g acetyl-DL-leucine per day taking 1 tablet of 500 mg each 3 times per day in the first week, 3 g per day taking 2 tablets of 500 mg each 3 times per day in the second week). Full dosage (5 g per day taking 3 tablets in the morning, 3 tablets at noon, and 4 tablets in the evening of 500 mg each) was maintained for 4 weeks. In the case of adverse events (AE), a down-titration to a minimum dosage of 1.5 g per day was permitted at the investigators’ discretion. Medication intake was at least 30 minutes before and 2 hours after a meal. Treatment adherence was assessed by counting returned tablets and by assessing the actual treatment duration in each period.^21^ Baseline assessment included clinical history and neurological assessment. Besides self-administered patient questionnaires, information about the amount of preceding physical and speech therapy were obtained. Blood samples were done for routine laboratory testing. Safety was monitored at all visits (eMethods in Supplement 2).

### Outcome Measures

Primary efficacy end point was the absolute change in SARA total score from (period-dependent) baseline to week 6. Clinical outcome measures were assessed at the pretreatment or period-dependent baseline, 2 weeks after up-titration, at the end of both 6-week treatment periods, and during the posttreatment follow-up visit. Secondary outcome measures included the patient-reported health-related quality of life assessed by the EuroQol–5 Dimensions–5 Level (EQ-5D-5L) questionnaire^22^ and the *z* score of the Spinocerebellar Ataxia Functional Index (SCAFI) composed of 3 subtests (8-meter walk [8MW] assessing gait, 9-hole peg test [9HPT] assessing limb ataxia, and timed speech task [PATA]).^23^ The self-perceived symptom burden concerning the comorbidities depression and fatigue were graded by the Beck Depression Inventory (BDI-II) and Fatigue Severity Scale (FSS).^24,25^ Sum score (range 0 to 63) was specified as outcome measure for BDI-II and mean score (range 1 to 7) for FSS (higher score values indicated greater impairment for both measurement instruments).^21^ Any AE or serious AE (SAE) were documented (eMethods in Supplement 2).

### Sample Size Calculation

Presuming a minimum clinically relevant difference in the SARA total score of 1.5 points (ie, the mean absolute change on active treatment 1.5 score points better than on placebo) and a standard deviation (SD) of the individual SARA change of 4.2, a sample size of 86 in total would have 90% power to detect a difference in means of 1.5, using a paired *t* test with a .05 2-sided significance level (nQuery Advisor 7.0). For this conservative estimate of the required sample size, an intrapatient correlation of 0 was assumed. However, we expected a positive correlation resulting in an increase of power. With an expected dropout rate of about 20%, the target sample size was 108 patients to be enrolled.

### Statistical Analysis

Intention-to-treat (ITT) efficacy analyses were performed for the full analysis set (FAS), which included all randomized patients who did not fail to satisfy a major entry criterion (diagnosis of cerebellar ataxia), irrespective whether they were treated or not. The per-protocol (PP) sample defined for sensitivity analyses consisted of all participants of the FAS who did not substantially deviate from the protocol (determined on a per-participant basis at the blind data review meeting before final database lock) and who were on treatment for at least 21 days in both periods (half of the preplanned duration), counting from the day of first intake. Therefore, a sufficient criterion for exclusion of a randomized patient from the PP sample was a missing second treatment period. Safety was analyzed in the safety set, comprising all patients who received the allocated study drugs. For the principal analysis, a mixed model for repeated measures (MMRM) was applied according to ITT, with the raw SARA total score as outcome measure assessed at each visit of both treatment periods in order to handle incomplete individual patient profiles. As fixed effects we specified factor variables for treatment (acetyl-DL-leucine vs placebo), visit, and treatment period. The full model contained a 3-way interaction term between time, treatment, and period, and a treatment-by-time interaction term, in order to allow testing for sequence or interaction effects. The principal model was used to derive (marginal) mean absolute changes in SARA total score from (period-dependent) baseline to posttreatment values, and to compare between both treatment conditions (difference in mean absolute change scores at week 6 predefined as the treatment effect of primary interest). Random intercepts were specified to account for patient-to-patient variation in symptom level at baseline visits. Competing MMRMs were compared by a likelihood ratio (LR) test to investigate whether treatment effects, period effects or cross-over effects (treatment-by-time interaction) were present. A supplementary responder analysis based on the exact McNemar test for matched pairs was performed defining a decline in SARA total score from baseline to week 6 of at least 1.5 points (symptom relief in an absolute sense) as threshold for a binary outcome of treatment success (or treatment failure otherwise) (eMethods in Supplement 2). The preplanned subgroup analyses (hereditary versus nonhereditary or unknown cerebellar ataxia) were conducted to investigate the homogeneity of treatment response concerning the primary outcome. For secondary efficacy outcomes, the same modelling approach was used to estimate treatment effects. To analyze the differences between both treatments at the end of the 6-week treatment periods, 95% CIs for target estimates were provided to quantitatively describe effects and to assess their clinical relevance (eMethods in Supplement 2). All statistical tests were 2-sided, with a significance level of *P* < .05. Statistical analyses were performed using the statistical software package R version 3.5.2 (R Project for Statistical Computing) from April 2018 to June 2018 and January 2020 to March 2020.

## Results

### Participants

**Figure 1.**
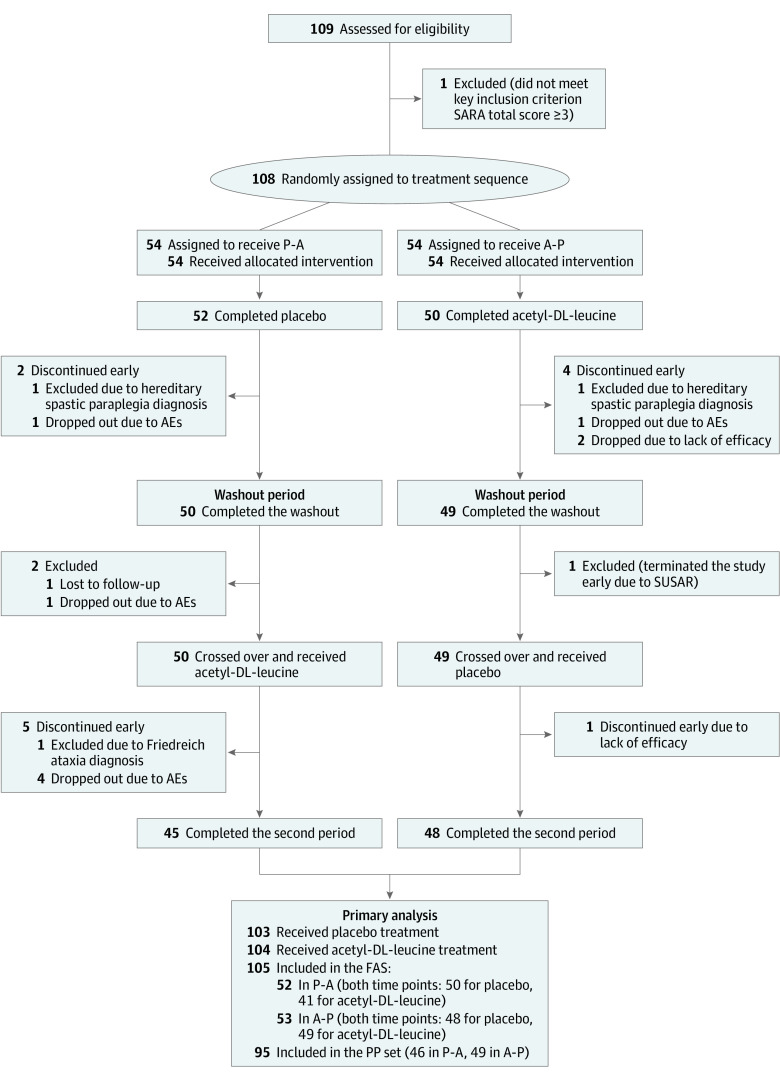
Study Flowchart Diagram Flow of patients through the trial and inclusion in the primary analysis. (Dropped out means study dropout with last contact, and no further data or visits documented.) A-P indicates treatment sequence acetyl-DL-leucine followed by placebo; AE, adverse event; FAS, full analysis set; P-A, treatment sequence placebo followed by acetyl-DL-leucine; PP, per protocol; SARA, Scale for the Assessment and Rating of Ataxia; SUSAR, suspected unexpected serious adverse reaction.

Between 2016 and 2017, 109 patients were assessed for eligibility and 108 patients were randomly assigned to sequence groups (54 patients each) (Table 1). Of the 108 patients randomized, 55 (50.9%) were female; the mean (SD) age was 54.8 (14.4) years. At enrollment, patients had symptoms for a median of 10 years (16 patients [14.8%] had symptoms for at least 20 years), and the mean (SD) SARA total score was 13.33 (5.57) points (median [IQR] SARA total score, 12.25 points [9.50-17.00 points]). Study visits occurred between January 25, 2016, and July 3, 2017. At baseline, there were no clinically relevant differences between sequence groups regarding demographics, clinical characteristics, and symptom scores (Table 1 and eTable 4 in Supplement 2). The full analysis set (FAS) included 105 patients (Figure 1). Eleven patients were misclassified owing to diagnostic uncertainties and reassessment based on genetic testing during the trial, 10 of these were part of the FAS (eTable 3 in Supplement 2). Ultimately, the FAS included 80 patients diagnosed with hereditary and 25 with nonhereditary or unknown cerebellar ataxia. In total, 95 patients (90.5%; 46 patients in the P-A sequence group and 49 in the A-P sequence group) in the FAS completed their treatment as per protocol. Overall, 88.6% (93 patients) completed the second treatment period (Figure 1). In the FAS, the mean treatment duration was comparable for acetyl-DL-leucine (39.32 days [95% CI, 37.13 to 41.52 days]) vs placebo (39.92 days [95% CI, 38.25 to 41.60 days]). Since no patients were misrandomized (ie, all patients received both treatments in the order they were assigned to), the safety and full analysis population were identical.

**Table 1.** Baseline Clinical and Demographic Characteristics of the Intention-to-Treat Population

Characteristics	Patients, No. (%) (N = 108)	
	Placebo followed by acetyl-DL-leucine (n = 54)	Acetyl-DL-leucine followed by placebo (n = 54)
Demographics		
Age, mean (SD), y	53.0 (14.3)	56.7 (14.3)
Sex		
Female	25 (46.3)	30 (55.6)
Male	29 (53.7)	24 (44.4)
Therapy prior to enrollment, median (range), min/wk		
Physical	40 (0-210)	50 (0-360)
Speech	0 (0-60)	0 (0-90)
Cerebellar ataxia subtypes^a^		
Hereditary	42 (77.8)	41 (75.9)
Nonhereditary	12 (22.2)	13 (24.1)
Exploratory subgroups		
SCA (autosomal dominant)	31 (57.4)	33 (61.1)
Autosomal recessive	6 (11.1)	2 (3.7)
Other types of hereditary (SCA)	5 (9.3)	6 (11.1)
Sporadic (SAOA)	12 (22.2)	13 (24.1)
Duration of cerebellar symptoms, mean (SD), y	11.77 (9.66)	11.39 (7.57)
Ataxia rating scales		
SARA, total score, mean (SD)	13.11 (5.10)	13.56 (6.03)
SCAFI *z* score, mean (SD)^b^	−0.15 (0.79)	−0.08 (1.00)
Self-report questionnaires		
EQ-5D-5L, health utility index, mean (SD)^c^	0.76 (0.22)	0.72 (0.22)
EQ VAS^d^		
Mean (SD)	59.94 (20.84)	65.19 (17.21)
Median (range)	70.00 (6.00-99.00)	62.50 (20.00-95.00)
BDI-II, sum score, mean (SD)^e^	11.50 (7.98)	10.57 (7.19)
FSS, mean score, mean (SD)^f^	4.11 (1.66)	4.06 (1.65)

Abbreviations: BDI-II, Beck Depression Inventory; EQ-5D-5L, EuroQol-5D-5L Questionnaire; FSS, Fatigue Severity Scale; SAOA, sporadic adult onset ataxia of unknown etiology; SARA, Scale for the Assessment and Rating of Ataxia; SCA, spinocerebellar ataxia; SCAFI, Spinocerebellar Ataxia Functional Index; VAS, Visual Analogue Scale.

^a^ Diagnosis hereditary (suspected or genetically confirmed) vs nonhereditary or unknown cerebellar ataxia (prespecified subgroups). If applicable, corrected after randomization (eTable 3 in Supplement 2).

^b^ Composite index calculated as the arithmetic mean of all 3 *z* scores (*z* scores for subtest 8m walk, 9-hole peg test, timed speech task, called PATA) according to the SCAFI Rating Manual. The individual *z* scores can be expressed as SD higher (positive *z* score) or lower (negative *z* score) than the baseline mean of the population under study in each subtest.

^c^ EQ-5D-5L utility index scores calculated for German value set (reference states: 1.00 = full health, 0 = death).

^d^ Visual analogue scale (range 0 to 100, the higher the better).

^e^ BDI-II sum score: range 0 to 63 (21 items, 4-point scale from 0 to 3, time frame: past 2 weeks), higher scores indicate greater impairment.

^f^ FSS total mean score: range 1 to 7 (9 items, 7-point scale from 1 to 7, time frame: within last week), higher scores indicate greater impairment.

### Primary Outcomes

**Figure 2.**
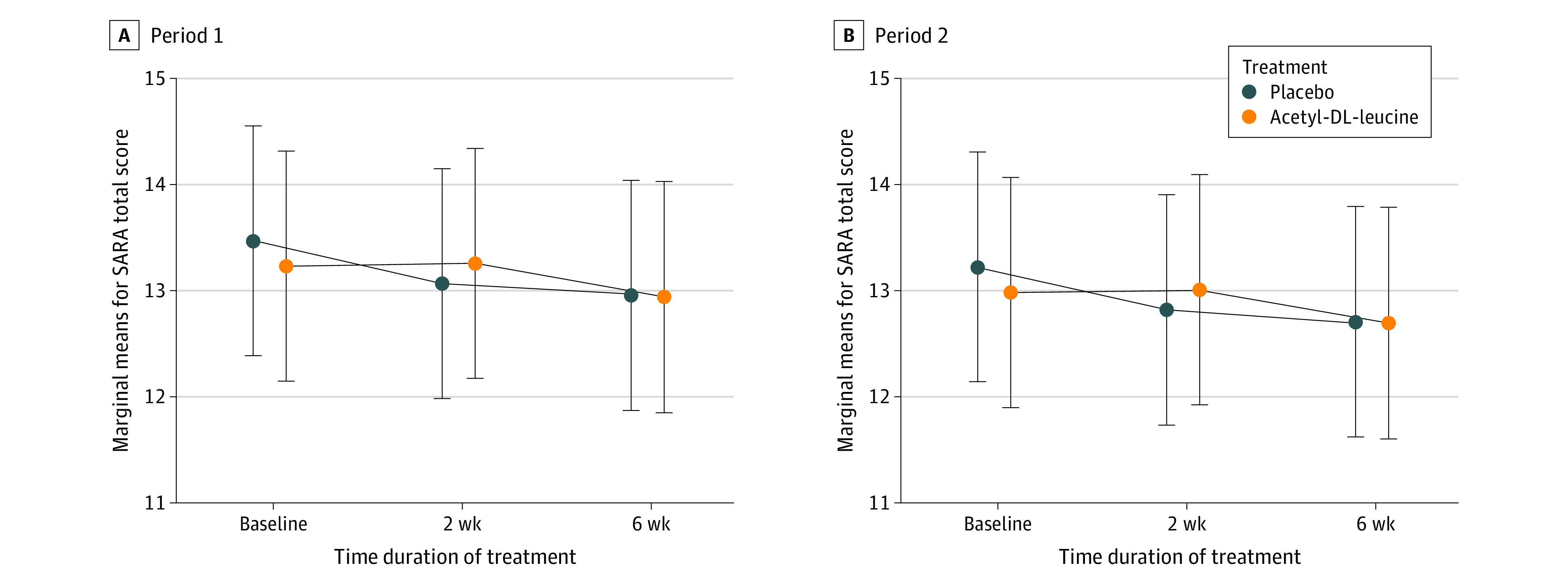
Principal Model-Based Analysis for the Primary Efficacy Outcome Scale for the Assessment and Rating of Ataxia (SARA) Total Score Interaction plot for estimated marginal means (with 95% CI) on acetyl-DL-leucine vs on placebo, at prerandomization or period-dependent baseline, after 2 weeks, and at the end of the 6-week treatment period. The fitted values are derived from a mixed model for repeated measures with treatment, time and period, and treatment-by-time interaction as fixed effects (all considered as factor variables), and patient-individual random intercepts.

The principal analysis found no evidence of a treatment benefit of acetyl-DL-leucine compared to placebo (Table 2 and Figure 2). The mean absolute change from baseline to week 6 in SARA total scores did not differ significantly between acetyl-DL-leucine and placebo (mean treatment difference: 0.23 points [95% CI, −0.40 to 0.85 points]; *P* = .48). There was some evidence for a time effect (*P* = .04, *F* test) (ie, a decline in SARA total score values within each treatment period). The period effect estimate was −0.25 points (95% CI, −0.50 to 0.01; *P* = .06) in SARA total score in period 2 compared to period 1 (eTable 2 in Supplement 2). Changes over time within periods and between both periods were not considered clinically relevant because this improvement in disease symptoms was far less than the prespecified threshold of 1.5 score points. At week 6, an overall mean reduction in SARA total score values of −0.40 points (95% CI, −0.78 to −0.03 points; *P* = .03) compared with the period-dependent baseline was observed, whereas at week 2, the overall mean difference was −0.19 points (95% CI, −0.56 to 0.18 points; *P* = .45). A sensitivity analysis of SARA total scores in the PP population and a supplementary analysis for the binary outcome treatment success (decrease in SARA total score of ≥1.5 points) revealed no statistically significant difference between acetyl-DL-leucine and placebo after 6 weeks, supporting the results of the principal analysis (eAppendix 1 and eAppendix 2 in Supplement 2). The proportion of missingness with respect to the primary outcome SARA was low due to the small number of dropouts (eFigure 2 in Supplement 2).

**Table 2.** Summary for the Primary Outcome in the Full Analysis Set of 105 Patients

	Marginal means (95% CI)		Acetyl-DL-leucine − placebo, mean difference (95% CI)^a^	*P* value^b^
	Acetyl-DL-leucine	Placebo		
SARA total score				
Baseline^c^	13.11 (12.03 to 14.18)	13.35 (12.27 to 14.42)	−0.24 (−0.68 to 0.20)	.28
Week 2	13.13 (12.06 to 14.21)	12.94 (11.87 to 14.02)	0.19 (−0.25 to 0.63)	.39
Week 6	12.82 (11.74 to 13.90)	12.83 (11.75 to 13.91)	−0.01 (−0.47 to 0.44)	.95
Changes in SARA total score from baseline				
Week 2	0.03 (−0.41 to 0.46)	−0.40 (−0.84 to 0.03)	0.43 (−0.18 to 1.05)	.17
Week 6	−0.29 (−0.74 to 0.16)	−0.52 (−0.95 to −0.08)	0.23 (−0.40 to 0.85)	.48

Abbreviation: SARA, Scale for the Assessment and Rating of Ataxia.

^a^ Contrast of primary interest: difference is the effect of treatment (acetyl-DL-leucine versus placebo) on the efficacy outcome.

^b^ *P* value from the mixed model for repeated measures (fixed effects: factor variables for treatment [acetyl-DL-leucine vs placebo], visit and treatment period, and treatment-by-visit interaction; random effects: patient-specific random intercepts). Estimated marginal means (least-squares means) derived from the mixed model, averaged over the levels of period.

^c^ Baseline means pretreatment or period-dependent baseline.

### Secondary Outcomes

In SCAFI *z* score, no treatment benefit of acetyl-DL-leucine compared with placebo could be found. Likewise, we identified a significant period effect, with higher SCAFI *z* scores in the second period (main effect for mean improvement in index values was 0.12 points [95% CI, 0.06 to 0.17 points; *P* < .001] compared with the first period).

In addition, there was no evidence for a clinically relevant effect of acetyl-DL-leucine on the subjective health rating EQ visual analogue scale compared with placebo at week 6 with no evidence of a period effect. At week 6, the marginal mean treatment difference between acetyl-DL-leucine and placebo in the overall self-rated health status was −1.84 points (95% CI, −5.19 to 1.50 points; *P* = .28) (Table 3). There was also no significant difference concerning the self-perceived impairment on BDI-II and FSS between acetyl-DL-leucine and placebo after 6 weeks. For BDI-II, a similar slight improvement from period-dependent baseline to week 6 was detected on both treatments, although reflecting no clinically relevant change (Table 3). Furthermore, there was no difference between both treatments with respect to the primary efficacy outcome SARA total score for the 2 prespecified key subgroups hereditary and nonhereditary cerebellar ataxia.

**Table 3.** Key Secondary Outcome Results in the Full Analysis Set

	Marginal means in secondary outcome (95% CI)^a^		Acetyl-DL-leucine − placebo, mean difference (95% CI)^b^	*P* value^c^
	Acetyl-DL-leucine	Placebo		
SCAFI, total *z* score^d^				
Baseline	−0.07 (−0.25 to 0.12)	−0.07 (−0.25 to 0.11)	0.01 (−0.05 to 0.06)	.83
Week 2	−0.02 (−0.20 to 0.16)	−0.05 (−0.23 to 0.13)	0.03 (−0.02 to 0.08)	.26
Week 6	−0.04 (−0.22 to 0.14)	−0.02 (−0.20 to 0.16)	−0.02 (−0.07 to 0.04)	.52
EQ VAS				
Baseline	64.55 (60.75 to 68.35)	60.88 (57.09 to 64.70)	3.67 (0.42 to 6.92)	.03
Week 2	64.43 (60.61 to 68.25)	63.65 (59.83 to 67.47)	0.78 (−2.51 to 4.07)	.64
Week 6	61.01 (57.13 to 64.88)	62.85 (59.03 to 66.67)	−1.84 (−5.19 to 1.50)	.28
BDI-II, sum score^e^				
Baseline	10.32 (8.68 to 11.96)	10.67 (9.03 to 12.30)	−0.35 (−1.32 to 0.63)	.49
Week 2	1.00 (8.36 to 11.64)	9.51 (7.87 to 11.15)	0.49 (−0.50 to 1.48)	.33
Week 6	9.79 (8.14 to 11.45)	9.69 (8.05 to 11.33)	0.10 (−0.91 to 1.11)	.85
FSS, mean score^f^				
Baseline	4.14 (3.79 to 4.48)	4.12 (3.77 to 4.46)	0.02 (−0.21 to 0.25)	.87
Week 2	4.15 (3.80 to 4.49)	4.06 (3.72 to 4.41)	0.09 (−0.15 to 0.32)	.47
Week 6	4.23 (3.88 to 4.58)	4.17 (3.82 to 4.51)	0.06 (−0.17 to 0.30)	.61

Abbreviations: BDI-II, Beck Depression Inventory; EQ VAS, EuroQol visual analogue scale; SCAFI, Spinocerebellar Ataxia Functional Index.

^a^ Estimated marginal means derived from the mixed model for repeated measures, averaged over the levels of period. Marginal means in secondary outcomes for pretreatment or period-dependent baseline, week 2, and week 6 representing the time point of primary interest.

^b^ Contrast of primary interest: difference means the effect of treatment (mean difference on acetyl-DL-leucine versus placebo) on the efficacy outcome.

^c^ *P* value from the mixed model for repeated measures (descriptive comparisons).

^d^ The SCAFI is a quantitative composite performance measure and was generated as the arithmetic mean of all 3 *z* scores. The individual *z* scores can thus be expressed as SD higher (positive *z* score) or lower (negative *z* score) than the baseline mean of the population under study in each subtest. Increases in SCAFI reflect improvement.

^e^ Higher BDI scores (range 0 to 63) indicate greater impairment.

^f^ Higher FSS scores (range 1 to 7) indicate greater impairment.

### Adverse Events

A total of 246 AE (86 patients with at least 1 AE) occurred in similar numbers in both sequence groups (A-P: 42 patients; P-A: 45 patients) with a median (range) of 2 (0-10) AEs per patient throughout his or her individual observation period. Of these, 8 AEs (3.3%) were assessed as serious (6 on acetyl-DL-leucine, 2 on placebo), whereas 191 (77.6%) were of mild intensity and 48 (19.5%) were of moderate intensity. No deaths were reported. Only 12 (4.9%) of all reported AEs (3 on acetyl-DL-leucine; 9 on placebo) were considered probably or likely treatment-related by the investigator (eTable 5 in Supplement 2). The most commonly reported AEs were within the system organ class of gastrointestinal disorders (17.9% of AEs [44 AEs for 35 patients]); nervous system disorders (15.0% [37 AEs for 32 patients]); general disorders and administration site conditions (13.4% [33 AEs for 28 patients]); or injury, poisoning, and procedural complications (13.4% [33 AEs for 28 patients]).

## Discussion

In our randomized, double-blind, placebo-controlled, crossover study, a 6-week treatment with acetyl-DL-leucine was not effective in patients with cerebellar ataxia. The difference in mean SARA total change scores at week 6 compared with baseline was 0.23 points (95% CI, −0.40 to 0.85 points) for the active vs placebo treatment; clearly not reflecting a convincing beneficial effect, while even the lower confidence limit was below a clinically relevant threshold being meaningful for the patient. Apparently, large treatment effects are not likely given the present results. SARA score as an objective clinical measure remained rather stable over the 6 weeks. These findings were supported by the SCAFI and all prespecified secondary outcomes. So far, encouraging data on the efficacy of acetyl-DL-leucine in cerebellar ataxia were based on small, open-label case series with a short treatment exposure and little regard to the effect of confounders.^17,18^ Animal studies provided a solid rationale for the use of acetyl-DL-leucine in other types of ataxia, namely lysosomal storage diseases.^11,12,13^ The optimal dosage and treatment duration of acetyl-DL-leucine remained uncertain due to uncertainties concerning the pharmacological mode of action.^26^ Given the presumed mode of action of acetyl-DL-leucine,^12,27^ we hypothesized that we would observe a rapid onset of a symptomatic effect during the treatment period of 6 weeks on acetyl-DL-leucine based on the observational data. ALCAT reached the sample size of 108 patients after a recruitment period of only 13 months. The key inclusion criterion defined by a SARA total score of at least 3 points enabled the enrollment of a substantial number of patients with only mild ataxia. Due to the crossover design, all participants had the possibility to receive the active treatment. There was no standard medical treatment to be withheld.^10^ Eligible patients were allowed to continue regular physical or speech therapy with unchanged intensity minimizing the risk of withdrawal. ALCAT included adults with diagnosed hereditary or nonhereditary forms of cerebellar ataxia with more than 20 different etiologies, having symptoms for a median of 10 years, 14.8% of them for more than 20 years. Therefore, ALCAT was not adequately powered to detect treatment effects and elicit heterogeneity in terms of treatment response in different ataxia subgroups. This also may have diluted positive effects. With only 25 patients (23.1%) classified as having nonhereditary ataxia, our findings may not be fully applicable to the German target population, assuming about 50% of the cases being sporadic. Moreover, 70 participants (64.8%) were enrolled at specialized sites bundling excellent, nationwide expertise in cerebellar ataxia and attracting patients from all over Germany.

In general, acetyl-DL-leucine was safe and well tolerated; 95 of 105 patients in the FAS (90.5%) completed ALCAT without major protocol deviations, which reduced the risk of bias due to a diluted true treatment effect. Only few patients discontinued due to an AE. For both treatments, AEs were mainly mild and occurred in similar numbers reporting symptoms of the gastrointestinal tract most frequently. This indicates an acceptable safety profile together with the known low nocebo effect in placebo-controlled drug trials on cerebellar ataxia.^28^ A major strength of ALCAT was the excellent compliance while taking acetyl-DL-leucine. The flow of participants through the trial resulted in a low loss of patients with respect to the randomization, suggesting a considerably high treatment adherence. The proportion of missingness with respect to the primary outcome SARA was low due to the small number of dropouts (eFigure 2 in Supplement 2).

### Limitations

This study had several limitations. Primarily, the treatment duration with 6 weeks was rather short compared with other parallel-group trials on cerebellar ataxia.^6,8^ It cannot be ruled out that in some patients the disease progression was too advanced for a symptomatic drug treatment to provide clinically relevant effects. Considering the responsiveness of SARA, the slow clinical progression over time, and variability of treatment response, our results suggest that limited changes in SARA can be revealed during the observation period of ALCAT. Notwithstanding, the clinimetric properties of SARA and SCAFI to detect clinically relevant, but small treatment effects in clinical trials with a short treatment duration remain unclear. Furthermore, the ataxia ratings were done by investigators who were involved in the clinical assessment increasing the risk of bias.^29^ With a design based on rather optimistic assumptions, the current trial cannot ascertain whether a short-term treatment benefit less than 0.5 score points could be established. The slight decline over time, smaller than the predefined threshold of 1.5 score points, might have been caused by clinical fluctuations.

## Conclusions

To our knowledge, ALCAT is the largest multicenter, double-blind, randomized, placebo-controlled crossover trial on acetyl-DL-leucine among patients with cerebellar ataxia of different etiologies. Although the efficacy end points were not met, ALCAT yielded valuable information about the duration of treatment periods and the role of placebo response in progressive disorders. These findings suggest the need for further symptom-oriented trials evaluating the long-term effects of acetyl-DL-leucine for well-defined subgroups of cerebellar ataxia. Further lessons to be drawn from ALCAT include the urgent necessity of the development of novel patient-centered efficacy end points being sensitive to clinical changes. Therefore, these end points would be more suitable for interventional trials with short-term therapies aiming to improve functioning and symptoms in established core domains.

## Notes

Supplement 1.
Trial Protocol

Supplement 2. **eMethods.** Supplemental Methods **eFigure 1.** Trial Design: Schedule of Trial Activities and Crossover Intervention Scheme **eTable 1.** Full List of Eligibility Criteria as Stated in the Trial Protocol **eTable 2.** Results of the Primary Analysis (Full Analysis Set [FAS]): Fixed Effects and Variance Components, Estimates of the Principal Model (MMRM) **eAppendix 1. **Per Protocol (PP) Analysis for Primary Outcome **eAppendix 2.** Supplementary Analysis (FAS): Binary Outcome Measure for Treatment Response **eTable 3.** Individual Patient Data Listings: Errors in Stratification During Randomization (Hereditary vs. Non-Hereditary/Unknown Etiology) **eTable 4.** Additional Baseline Data for ITT Population **eTable 5.** Adverse Events (AEs) by Outcome, Grade, Attribution, and Seriousness in the Safety Set **eFigure 2.** Missingness Plot for SARA Total Score (FAS Population)
eReferences

Supplement 3. **Nonauthor Collaborators**. ALCAT Study Group

Supplement 4.
Data Sharing Statement
