# Supplementary material for: Safety and Efficacy of Acetyl-DL-Leucine in Certain Types of Cerebellar Ataxia: The ALCAT Randomized Clinical Crossover Trial
Source: JAMA Netw Open. 2021 Dec 14;4(12):e2135841. doi: 10.1001/jamanetworkopen.2021.35841 (PMC8672236; doi:10.1001/jamanetworkopen.2021.35841)
Supplement: Supplement 1. — Trial Protocol [file jamanetwopen-e2135841-s001.pdf]

Clinical Trial Protocol

Effects of Acetyl-DL-Leucine on cerebellar ataxia - a multinational, multicenter, randomized, double-blind, placebo-controlled, 2-way crossover phase III trial (ALCAT)

Version V1.0 – 09.09.2015

**Coordinating Investigator and Leiter der klinischen Prüfung (according to German Drug Law § 42)**	Michael Strupp, MD, FANA, FEAN<br>Professor of Neurology<br>Department of Neurology and German Center for Vertigo and Balance Disorders (DSGZ)<br>University Hospital Munich, Campus Großhadern<br>Marchioninistr. 15<br>81377 Munich, Germany<br>Phone: +49-89-4400-73678<br>Fax: +49-89-4400-76673<br>E-mail: michael.strupp@med.uni-muenchen.de
**Representative of Coordinating Investigator:**	Katharina Feil, MD<br>Department of Neurology and German Center for Vertigo and Balance Disorders (DSGZ)<br>Marchioninistraße 15<br>81377 Munich, Germany<br>E-mail: katharina.feil@med.uni-muenchen.de
**Sponsor / Sponsor Delegated Person:**	**Sponsor**:<br>Hospital of the University of Munich, represented by<br>Prof. Dr. Karl-Walter Jauch<br>University Hospital Munich, Campus Großhadern<br>Marchioninistr. 15<br>81377 Munich, Germany

	**Sponsor Delegated Person**:<br>Michael Strupp, MD, FANA, FEAN<br>Professor of Neurology<br>Department of Neurology and German Center for Vertigo and Balance Disorders (DSGZ)<br>University Hospital Munich, Campus Großhadern<br>Marchioninistr. 15<br>81377 Munich, Germany<br>Phone: +49-89-4400-73678<br>Fax: +49-89-4400-76673<br>E-mail: michael.strupp@med.uni-muenchen.de
**Trial Design:**	Multinational, multicenter, randomized, double-blind, placebo-controlled, 2-treatment 2-period crossover phase III efficacy of treatment trial
**Trial Code:**	ALCAT
**EudraCT No.:**	2015-000460-34
**Authors:**	Michael Strupp, Otmar Bayer, Ingrid Berger, Katharina Feil, Julian Teufel, Ulrich Mansmann, Christine Adrion

Confidential

The content of this document is strictly confidential and may not be copied or made accessible to third parties without prior written consent.

This document template was adapted by the clinical study center of the Deutsches Schwindel- und Gleichgewichtszentrum (DSGZ).

Protocol Approval Signatures

Sponsor/Sponsor Delegated Person (SDP):

Michael Strupp, MD, FANA, FEAN

Professor of Neurology

Department of Neurology and Deutsches Schwindel- und Gleichgewichtszentrum (DSGZ)

University Hospital Munich, Campus Großhadern

Marchioninistr. 15, 81377 Munich, Germany

16/9/15

Date

Signature

Coordinating Investigator (multi-center) and Leiter der klinischen Prüfung/LKP (in accordance with German Drug Law)

Michael Strupp, MD, FANA, FEAN

Professor of Neurology

Department of Neurology, and Deutsches Schwindel- und Gleichgewichtszentrum (DSGZ)
University Hospital Munich, Campus Großhadern

Marchioninistr. 15, 81377 Munich, Germany

16/9/15

Date

Signature

Responsible Biometrician:

Prof. Dr. rer. nat. Ulrich Mansmann

Institute for Medical Informatics, Biometry and Epidemiology (IBE)

LMU Munich, Campus Großhadern

Marchioninistr. 15

81377 Munich, Germany

15. 9. 2015

Date

Signature

Investigator Agreement Page

Place stamp of the investigational site here

By my signature below, I confirm that I have read, understood and agree to adhere to all conditions, instructions and restrictions as specified in this Clinical Trial Protocol.

I will discuss the Clinical Trial Protocol in detail with my colleagues and ensure that they are comprehensively informed about the trial compound and the execution of the clinical trial.

I confirm that I and my colleagues will conduct this clinical trial in compliance with the Declaration of Helsinki, the ICH-GCP guidelines, and that I will abide by the national laws and regulations.

Furthermore I and my colleagues commit ourselves not to commence subject enrollment before the authorization of the authorities, the acceptance by the relevant and responsible Ethics Committee and the legally valid conclusion of contract by the authorized representation of my institution concerning this clinical trial.

I recognize that any changes in the protocol must be approved by the Sponsor/Sponsor Delegated Person (SDP), the Ethics Committee and, if applicable, the respective authority before implementation except when necessary to eliminate hazards to the subjects or when changes involve only logistical or administrative aspects of the clinical trial.

Under my supervision I will allocate copies of this Clinical Trial Protocol and possible updates as well as access to all information regarding the carrying out of this clinical trial at the disposal of my colleagues; in particular I will promptly forward all information from the Sponsor/Sponsor Delegated Person (SDP) in relation to pharmaceutical safety (SUSAR) to my colleagues.

I agree to ensure that the confidential information contained in this document will not be used for any purpose other than the evaluation or conduct of the clinical investigation without prior written consent of the Sponsor/Sponsor Delegated Person (SDP).

The investigational medicinal products will be used only for the purpose of the clinical trial.

Investigator:

______________________________	______________________________
printed name	date, signature

Table of Contents

Protocol Approval Signatures ……………………………………………………………………………3

Investigator Agreement Page ……………………………………………………………………………4

Table of Contents 5

1 Clinical Trial Synopsis 10

2 Schedule of Activities and Assessments 18

3 Abbreviations 19

4 Trial Administration structure 21

5 Introduction 27

5.1 Background 27

5.2 Trial Rationale 30

5.3 Side effects and Risk Benefit Assessment 30

5.3.1 Side effects 30

5.3.2 Risk Benefit Assessment 32

6 Trial Objectives and Endpoints 33

6.1 Primary Objective 33

6.2 Primary Efficacy Endpoint 33

6.3 Secondary Objectives 33

6.4 Secondary Efficacy endpoint 33

6.5 Safety Variables 34

7 Trial Design 35

7.1 Trial Design 35

7.2 Intervention scheme 35

7.3 Number of centers 35

7.4 Number of patients 36

7.5 Time Schedule 36

8 Trial Population and Eligibility Criteria 37

8.1 Gender Distribution 37

8.2 Inclusion Criteria 37

8.3 Exclusion Criteria ... 37

8.4 Subject Information and Recruitment ... 39

8.5 Randomization and allocation concealment ... 39

9 Investigational Medicinal Product (IMP) ... 40

9.1 Specification of IMP ... 40

9.2 Packaging and Labelling of IMP ... 41

9.3 Transport of IMP ... 42

9.4 Storage requirements ... 42

9.5 Dosage, Mode of Application and Dose Schedule ... 42

9.6 Handling of IMP at the Site and Drug Accountability ... 43

9.7 Procedures for Monitoring the Subject Compliance ... 43

9.8 Return and Disposal of IMP ... 43

9.9 Blinding and Emergency Codes ... 44

9.10 Unblinding (Code break) ... 44

9.11 Prior and Concomitant care and intervention ... 45

9.11.1 Previous therapy / medication of trial specific illness ... 45

9.11.2 Previous therapy / medication of other indications than the trial specific illness ... 45

9.11.3 Prohibited concomitant therapy / medication for trial specific illness ... 45

9.11.4 Concomitant therapy / medication for other indications ... 46

10 Trial Procedures ... 47

10.1 Methods of Assessment ... 47

10.1.1 Scores for ataxia ... 47

10.1.2 Questionnaires for quality of life, depression and fatigue ... 48

10.1.3 Laboratory examinations / Biological Specimens ... 48

10.2 Time schedule of Measurements ... 50

10.2.1 Screening period ... 50

10.2.2 Treatment period 1 ... 50

10.2.3 Wash out ... 51

10.2.4 Treatment period 2 ... 52

10.2.5 Follow-up period ... 53

10.2.6 Premature termination of trial / drop-out visit ... 53

11 Safety Data Collection, Recording and Reporting ... 54

11.1 Definitions ... 54

11.2 Criteria to be evaluated by the investigator (1st assessment) ... 55

11.2.1 Assessment of Intensity ... 55

11.2.2 Assessment of Seriousness ... 56

11.2.3 Assessment of Causality ... 56

11.3 Criteria to be evaluated by the Sponsor Delegated Person (2nd assessment) ... 56

11.4 Documentation and Reporting of Adverse Events ... 56

11.5 Documentation and Reporting of Serious Adverse Events ... 57

11.5.1 Initial Reporting of SAEs ... 57

11.5.2 Reporting to the authorities and ethics committees ... 57

11.6 Pregnancy ... 58

11.6.1 Actions to be taken if pregnancy occurs to female subjects or partners of male subjects .. 58

12 Data Safety Monitoring Board (DSMB) ... 59

13 Statistical Methods ... 60

13.1 Planned Statistical Analyses ... 60

13.2 Interim Analysis ... 61

13.3 Sample Size Calculation ... 61

13.4 Populations included in the Analysis ... 61

13.5 Protocol Violations ... 61

13.6 Handling of Dropouts, Withdrawal, and Missing Data ... 62

14 Data Collection, Handling and Record Keeping ... 63

14.1 Data Management ... 63

14.2 Data Coding ... 63

14.3 Documentation of Trial Data ... 63

14.3.1 Documentation of Trial Data in the Medical Record ... 63

14.3.2 Case Report Form (CRF) ... 64

14.4 Trial site File ... 64

14.5 Archiving ... 65

14.5.1 Sponsor ... 65

14.5.2 Investigator ... 65

15 Reporting ... 66

15.1 Statistical Report ... 66

16 Definition of End of Trial ... 66

16.1 Regular End of the Trial ... 66

16.2 Termination of the Trial for Individual Subjects ... 66

16.2.1 Termination by the Subject ... 67

16.2.2 Termination by the Investigator ... 67

16.3 Termination of the Trial in Individual Sites ... 68

17 Monitoring, Audits and Inspections ... 69

17.1 Monitoring ... 69

17.2 Source Data Verification (SDV) ... 69

17.3 Audits and Inspections ... 70

18 Ethics and Good Clinical Practice ... 71

18.1 Responsibilities of the Sponsor ... 71

18.2 Responsibilities of the Investigator ... 71

18.3 Ethics Committee and Competent Authority(ies) ... 72

18.4 Compliance with the Protocol ... 72

18.5 Notification of General Amendments to the Protocol ... 73

18.6 Notification of the end of the trial ... 73

18.7 Annual Safety Report ... 74

18.8 Subject Information and Informed Consent ... 74

18.9 Subject Insurance ... 75

18.10 Data Protection and Subject Confidentality ... 76

18.11 Financing of the Trial ... 76

18.11.1 Trial Agreement / Investigator Compensation ... 76

18.11.2 Reimbursement of Subjects ... 77

19 Trial Reports ... 77

20 Publication ... 77

20.1 Publication Policy ... 77

21 References ... 78

22 Appendix ... 80

22.1 Ataxia score: SARA ... 80

22.2 Ataxia score: SCAFI ... 82

22.3 Questionnaire for quality of life: EQ-5D-5L ... 84

22.4 BDI-II ... 87

22.5 FSS ... 89

1 Clinical Trial Synopsis

**Clinical trial title**	Effects of Acetyl-DL-Leucine on cerebellar ataxia - a multicenter, randomized, double-blind, placebo-controlled, 2-way crossover phase III trial (ALCAT)
**Phase of trial**	III
**Trial Code**	ALCAT
**EudraCT No.**	2015-000460-34
**Investigational medicinal product, Dose and Mode of Application**	**TANGANIL®**<br>Substance: Acetyl-DL-Leucine<br>Manufacturer: Pierre Fabre GmbH<br>15-17 Avenue du Sidobre<br>81100 Castres, France<br>Strength: 500 mg<br>Mode of application: oral<br>Duration of treatment: 6 weeks<br>Dose schedule: Week 1 — 1.5 g per day — 1-1-1<br>Week 2 — 3 g per day — 2-2-2<br>Week 3-6 — 5 g per day — 3-3-4<br><br>**Corresponding Placebo**<br>Substance: NA<br>Manufacturer: Haupt Pharma Wuelfing GmbH<br>Bethelner Landstrasse 18<br>31028 Gronau, Germany<br>Strength: NA<br>Mode of Application: oral<br>Duration of treatment: 6 weeks

	Dose schedule	Week 1	per day	1-1-1
		Week 2	per day	2-2-2
		Week 3-6	per day	3-3-4
**Trial Population**	Adult male and female subjects with cerebellar ataxia (CA) of different etiologies (hereditary or non-hereditary)			
**Trial Design**	Multinational, multicenter, randomized, double-blind, placebo-controlled, 2- period 2-treatment crossover phase III superiority trial (efficacy of treatment trial)			
**Trial Objectives**	Primary objectives:<br>To demonstrate that Acetyl-DL-Leucine is efficacious in improving motor function measured by the Scale for the Assessment and Rating of Ataxia (SARA) total score<br><br>Secondary objectives:<br>To demonstrate that Acetyl-DL-Leucine is efficacious in<br>a) improving motor function measured by the Spinocerebellar Ataxia Functional Index (SCAFI) and SARA subscore items<br>b) improving quality of life (EQ-5D-5L) as well as depression (Beck Depression Inventory, BDI-II) and fatigue (Fatigue Severity Score, FSS)<br>To check for the occurrence of adverse effects of the trial drug			
**Trial Endpoints**	Primary efficacy endpoint:<br>SARA total score: absolute changes at the end of each treatment period compared to the period-level baseline defined as SARA score at visit 2 (treatment period 1) and at visit 5 (treatment period 2)<br><br>Key secondary endpoint(s):<br>SCAFI and subscores of SARA, QoL (EQ-5D-5L), depression (BDI-II), fatigue (FSS): absolute changes at the end of the 6-week treatment period compared to the period-level baseline defined as score at visit 2 (treatment period 1) and at visit 5 (treatment period 2).<br>Additionally, change scores at follow-up visit will be assessed.<br><br>Assessment of safety:<br>Documentation and reporting of side effects (AEs, SAEs, SUSARs)			

**Subject Number**	To be assessed for eligibility: n = 200 patients<br>To be allocated to trial: n = 108 patients<br>To be analysed: n = 86 patients (expected to complete both phases)
**Inclusion Criteria**	Subjects will only be included in the study if they meet all of the following criteria:<br>• Clinically confirmed cerebellar ataxia (CA) with a total SARA-Score ≥ 3 (range 0-40) of hereditary or non-hereditary degenerative type<br>• Patient did not receive any of the following prohibited medication within 4 weeks prior to randomization:<br>o Aminopyridines (including substained-release form)<br>o Acetyl-DL-Leucine<br>o Riluzole<br>o Gabapentin<br>o Varenicline<br>o Chlorzoxazone<br>• The ability to follow study instructions and likely to attend and complete all required visits<br>• Written informed consent of the subject prior to any study specific intervention<br>• Age ≥ 18 years
**Exclusion Criteria**	Subjects will not be included in the study if any of the following criteria applies:<br>• Subject is not able to give consent<br>• Onset of ataxia in association with stroke, encephalitis, sepsis, hyperthermia or heat stroke<br>• Toxic causes for ataxia of cerebellar type<br>• Rapid progression of ataxia (development of severe ataxia in less than 12 weeks)<br>• Subject suffers from any of the following:

	o chronic diarrhea<br>o unexplained visual loss<br>o malignancies<br>o insulin-dependent diabetes mellitus<br>• Ataxia due to multiple sclerosis, ischemia, hemorrhage or tumor of the posterior fossa as confirmed by imaging<br>• Ataxia due to clinical likely multisystem atrophy type C (MSA-C)<br>• Diagnosis of clinical likely Friedreich ataxia<br>• Known history of hypersensitivity to the investigational drug or derivates<br>• Liver failure defined as AST/ALT > 300 U/l<br>• Simultaneous participation in another clinical trial or participation in any clinical trial involving administration of an investigational medical product within 30 days prior to the beginning of the clinical trial<br>• Subjects with a physical or psychiatric condition which at the investigator's discretion may put the subject at risk, may confound the trial results, or may interfere with the subject's participation in this clinical trial<br>• Known or persistent abuse of medication, drugs or alcohol<br>• Females of childbearing potential, who are not using and not willing to use medically reliable methods of contraception for the entire study duration as listed in the patient informed consent form<br>• Current or planned pregnancy or nursing women<br>• Patients has received any of the following prohibited medication within 4 weeks prior to randomization<br>o Aminopyridines (including substained-release form)<br>o Acetyl-DL-Leucine<br>o Riluzole<br>o Gabapentin<br>o Varenicline

	o Chlorzoxazone<br><br>The indication specific exclusion criteria are chosen based on the available data on Acetyl-DL-Leucine of the French Agence nationale de sécurité du médicament et des produits de santé (http://agence-prd.ansm.sante.fr/php/ecodex/notice/N0126720.htm, visited at 24.04.2015, dated on 20.02.2007), in particular hypersensitivity to the agent.
**Trial Procedures**	Before screening visit/visit 1:<br>• Written informed consent<br><br>Treatment period 1: visit 2, 3 and 4<br>Treatment period 2: visit 5, 6 and 7<br>Wash out period of 4 weeks: between visit 4 and 5<br>Follow up/ close-out: visit 8<br><br>Screening visit/visit 1<br>• Check of inclusion/exclusion criteria<br>• Blood test (clinical chemistry: sodium, potassium, creatinine, serum bilirubin level, AST, ALT, urea, ALP, TSH; blood count: hemoglobin, erythrocytes, hematocrit, thrombocytes, leukocytes<br>• Pregnancy test for women of child bearing potential<br>• Documentation of adverse events<br>• Patient characteristics and medical history as well as medical history concerning the trial specific illness, including frequency of physiotherapy and speech therapy (documented in hours per week)<br>• Drug history including documentation of prior therapies/medications and concomitant medications for treatment of trial specific illness as well as drug history for other indications<br>• Documentation of concomitant medication<br>• Neurological examination<br>• Ataxia rating scale (SARA)

	<u>Visit 2 and 5 (start of treatment period)</u><br>• Randomization (at visit 2)<br>• Dispensing of trial drug<br>• Ataxia rating scales (SARA, SCAFI)<br>• Self-administered questionnaires (EQ-5D-5L, BDI-II, FSS)<br>• Documentation of adverse events<br>• Documentation of frequency of physiotherapy and speech therapy (hours per week)<br>• Documentation of concomitant medications<br>• Blood test (clinical chemistry: sodium, potassium, creatinine, serum bilirubin level, AST, ALT, urea, ALP, TSH; blood count: hemoglobin, erythrocytes, hematocrit, thrombocytes, leukocytes) (only at visit 5)<br>• Pregnancy test for women of child bearing potential (only at visit 5)<br><br><u>Visit 3, 4, 6, 7 and visit 8 (follow-up)</u><br>• Ataxia rating scales (SARA, SCAFI)<br>• Quality of life questionnaires (EQ-5D-5L, BDI-II, FSS)<br>• Documentation of adverse events<br>• Documentation of frequency of physiotherapy and speech therapy (hours per week)<br>• Documentation of concomitant medication<br>• Blood test (<u>clinical chemistry</u>: sodium, potassium, creatinine, serum bilirubin level, AST, ALT, urea, ALP, TSH; <u>blood count</u>: hemoglobin, erythrocytes, hematocrit, thrombocytes, leukocytes) (at visit 4 and 7)<br>• Pregnancy test for women of child bearing potential (visit 4 and 7)<br>• Return of trial drug at visit 4 and 7<br>• Compliance check at visit 3, 4, 6 and 7

**Trial Specific Measurements**	• Ataxia scores (SARA, SCAFI)<br>• Self-administered questionnaires (FSS, BDI-II, EQ-5D-5L)<br>• Blood tests including pregnancy tests
**Investigational Trial Sites**	This is a multi-center, international trial with 7 investigational trial sites in Europe planned at the time of finalization of the protocol.
**Statistical Rationale**	Efficacy:<br>The efficacy of the experimental intervention will be determined by testing and estimating differences regarding the absolute change in SARA total score measured at the 2-week visit and at the end of the verum/ placebo period compared to period-level baseline at visit 2 (treatment period 1) and at visit 5 (treatment period 2)<br><br>Description of the primary efficacy and population:<br>To analyze the differences in SARA total score between both treatments, a linear mixed effects model for DeltaSARA for the absolute change in SARA total score will be performed (fixed effects: factors for treatment visit, treatment-by-visit interaction, mean of both period-level baseline SARA total scores as covariate; patient-specific random intercepts).<br>The primary efficacy analysis will be performed according to the intention-to-treat (ITT) principle. Sensitivity analyses to assess robustness of trial results include multiple imputation techniques for the missing primary outcome. The significance level is alpha = 5%.<br><br>Safety:<br>Data about adverse events will be descriptively analyzed by verum and placebo phase.<br><br>Secondary endpoints:<br>The confirmatory analysis extends to the key secondary endpoint EQ-5D-5L if a benefit in the primary analysis could be demonstrated. Then this analysis will also be based on the ITT principle. Further secondary endpoints are the subscores of SARA and SCAFI, BDI-II, and FSS, which are not considered in the hierarchical multiple testing procedures and therefore will be analyzed descriptively (mainly by the Wilcoxon signed rank test where considered appropriate). The corresponding confidence intervals for treatment group effects will be reported. Change scores will be assessed at the 6-week visit at the end of the verum/ placebo

	treatment period, and at the follow-up visit 8.
**Time Schedule**	Per Subject:<br><br>Twenty weeks in total, including 2 x 6 weeks of verum/placebo treatment, 4 weeks wash-out between both treatment periods and a follow-up visit 4 weeks after last IMP intake. Patients receiving prohibited medication (see exclusion criteria) at visit 1 have to perform 4 weeks of wash-out prior to randomization.<br><br>Trial duration:<br><br>• Recruitment period (months): 16<br>• Planned Start Date (FPFV): 01.12.2015<br>• Planned End Date (LPLV): 01.08.2017<br>• First patient in to last patient out (months): 20<br><br>Duration of the entire trial (months): 32

Date and version identifier

Revision Chronology:

04.09.2015	Original Version V1.0

2 Schedule of Activities and Assessments

A delay of -3 and +5 days is acceptable for visit 2 and 5, for all other visits a delay of ± 5 days is acceptable.

	Enrollment		Treatment period 1				Treatment period 2			Close-out
	Before	Screening Visit 1♦	Visit 2	Visit 3 2 weeks after V2	Visit 4 6 weeks after V2	Wash-out: 4 weeks	Visit 5	Visit 6 2 weeks after V5	Visit 7 6 weeks after V5	Follow-up Visit 8 4 weeks after V7
Timeline (days)		*0/-28*	*0*	*14*	*42*	*70*	*70*	*84*	*112*	*140*
Informed consent[1]	X									
Inclusion / exclusion criteria		X								
Patient history, demographics		X								
Documentation of physiotherapy/speech therapy		X	X[2]	X	X		X	X	X	X
Neurological examination		X								
Blood tests		X*			X*		X*		X*	
Randomization			X							
Dispensing of trial drug			X				X			
Return of trial drug					X				X	
Compliance check				X	X			X	X	
Patient questionnaires (EQ-5D-5L, BDI-II, FSS)			X	X	X		X	X	X	X
Ataxia rating scale: SARA		X	X[2]	X	X		X	X	X	X
Ataxia rating scale: SCAFI			X	X	X		X	X	X	X
Documentation of (S)AE		X	X[2]	X	X		X	X	X	X
Documentation of concomitant medication (drug history)			X	X	X		X	X	X	X

[1] prior to first study specific intervention

[2] if visit 1 and visit 2 take place at the same time, ataxia rating scale (SARA), documentation of physiotherapy/speech therapy and documentation of (S)AE are assessed only once

♦ if patients are on medication due to cerebellar symptoms at visit 1, there is a planned 4-week-wash-out between visit 1 and 2 prior to randomization. Otherwise visit 1 and visit 2 should take place at the same time.

* incl. negative pregnancy test for women of child bearing potential

3 Abbreviations

**8MW**	8 meter walk, subscore of SCAFI
**9HPT**	9-hole peg test, subscore of SCAFI
**AR**	Adverse (Drug) Reaction
**AE**	Adverse Event
**AMG**	Arzneimittelgesetz
**BOB**	Bundesoberbehörde
**BDI-II**	Beck-Depressions-Inventar
**BfArM**	Bundesinstitut für Arzneimittel und Medizinprodukte
**CRA**	Clinical Research Associate
**CRF**	Case Report Form
**CRO**	Contract Research Organization
**DSGZ**	Deutsches Schwindel- und Gleichgewichtszentrum
**DSMB**	Data Safety Monitoring Board
**DRKS**	Deutsches Register Klinischer Studien
**EC**	Ethics Committee
**EQ-5D-5L**	EuroQuol-5D-5L
**FPFV**	First Patient First Visit
**FSS**	Fatigue Severity Scale
**GCP**	Good Clinical Practice
**IBE**	Institute for Medical Informatics, Biometry and Epidemiology
**ICH**	International Conference on Harmonization
**IMP**	Investigational Medicinal Product
**ISF**	Trial site File
**LPLV**	Last Patient Last Visit
**PEI**	Paul Ehrlich Institute
**PATA**	subscore of SCAFI
**SAR**	Serious Adverse Reaction
**SAE**	Serious Adverse Event

**SARA**	Scale for the assessment and rating of ataxia
**SCAFI**	Spinocerebellar ataxia functional index
**SDP**	Sponsor Delegated Person
**SmPC**	Summary of Product Characteristics
**SUSAR**	Suspected Unexpected Serious Adverse Reaction
**UAR**	Unexpected Adverse Reaction
**VAS**	Visual Analogue Scale

4 Trial Administration structure

**Coordinating Investigator and Leiter der klinischen Prüfung (according to German Drug Law)**	Prof. Dr. Dr. h.c. Michael Strupp, MD, FANA, FEAN<br>Professor of Neurology<br>Department of Neurology and Deutsches Schwindel- und Gleichgewichtszentrum (DSGZ)<br>University Hospital Munich, Campus Großhadern<br>Marchioninistr. 15<br>81377 Munich, Germany<br>Phone: +49-89-4400-73678<br>Fax: +49-89-4400-76673<br>E-mail: michael.strupp@med.uni-muenchen.de
**Sponsor**	Hospital of the University of Munich, represented by<br>Prof. Dr. Karl-Walter Jauch<br>Marchioninistr. 15<br>81377 Munich, Germany<br>Phone: +49-89-4400-72101<br>Fax: +49-89-4400-72102<br>E-mail: karl-walter.jauch@med.uni-muenchen.de
**Coordinating research center**	Dr. Otmar Bayer, MD, MPH<br>Clinical Study Center within the German Center for Vertigo and Balance Disorders (DSGZ), LMU Munich<br>University Hospital Munich, Campus Großhadern<br>Marchioninistr. 15<br>81377 Munich, Germany<br>Phone: +49-89-4400-76986<br>Fax: +49-89-4400-78795<br>E-Mail: otmar.bayer@med.uni-muenchen.de
**Participating trial sites:**	This clinical trial is planned as a multicenter double-blind randomized trial at 7 trial sites in Europe. If necessary, further qualified trial sites may be recruited.

	The trial sites with names of the principal investigators given here is as of finalization of the study protocol. The listing of trial sites, principal investigators, sub-investigators, and further trial staff, will be kept and continuously updated in a separate list. The final version of this list will be attached to the final report of the clinical trial.
	**Prof. Dr. Dr. h.c. Michael Strupp, MD, FANA, FEAN**<br>Professor of Neurology<br>Department of Neurology and Deutsches Schwindel- und Gleichgewichtszentrum (DSGZ)<br>University Hospital Munich, Campus Großhadern<br>Marchioninistr. 15<br>81377 Munich, Germany<br>Phone: +49-89-4400-73678<br>Fax: +49-89-4400-76673<br>E-mail: michael.strupp@med.uni-muenchen.de
	**Dr. med. Heike Jacobi**<br>German Center for Neurodegenerative Diseases (DZNE)<br>Center for clinical research/Zentrum für klinische Forschung (ZKF)<br>Ernst-Robert-Curtius-Str. 12<br>53117 Bonn, Germany<br>Phone: +49(0)228-43302-833<br>Fax: +49(0)228-43302-812<br>E-Mail: heike.jacobi@dzne.de
	**Prof. Dr. med. Hans-Christoph Diener**<br>Director of Neurology<br>University Clinic Essen<br>Hufelandstr. 55<br>45147 Essen, Germany<br>Phone: +49(0)201-723--2461

	Fax: +49(0)201-723-5901<br>E-Mail: hans.diener@uk-essen.de
	**Prof. Dr. med. Ludger Schöls**<br>Professor of Neurology<br>Department of Neurology, University Tübingen<br>Hoppe-Seyler-Str. 3<br>72076 Tübingen, Germany<br>Phone: +49(0)7071-29-82057<br>Fax: +49(0)7071-29-4254<br>E-mail: Ludger.Schoels@uni-tuebingen.de
	**Prof. Dr. med. Thomas Klopstock**<br>Professor of Neurology<br>Department of Neurology, Friedrich-Baur-Institute<br>University Hospital Munich<br>Ziemssenstr. 1a<br>80336 Munich, Germany<br>Phone: +49(0)89-4400-57400<br>Fax: +49(0)89-4400-57402<br>E-mail: thomas.klopstock@med.uni-muenchen.de
	**PD Dr. med. Sylvia Bösch**<br>Department of Neurology, University Innsbruck (Austria)<br>Anichstraße 35<br>A-6020 Innsbruck<br>Phone: +43(0)512-504-81815<br>Fax: +43(0)512-504-26286<br>E-Mail: sylvia.boesch@i-med.ac.at
	**Dr. B. van de Warrenburg**<br>Department of Neurology

	Radboud University Nijmegen Medical Centre (Netherlands)<br>P.O. Box 9101<br>6500 HB Nijmegen<br>The Netherlands<br>Phone: +31-24-3613396<br>Fax: +31-24-3618837<br>E-mail: B.vandeWarrenburg@neuro.umcn.nl

**Statistician / Biometrician:**	Prof. Dr. rer. nat. Ulrich Mansmann<br>Institute for Medical Informatics, Biometry and Epidemiology (IBE), LMU Munich, Campus Großhadern<br>Marchioninistr. 15<br>81377 Munich, Germany<br>Phone: +49-89-4400-74491<br>Fax: +49-89-4400-77491<br>E-Mail: mansmann@ibe.med.uni-muenchen.de
**Data Management:**	Institute for Medical Informatics, Biometry and Epidemiology (IBE), LMU Munich, Campus Großhadern<br>Marchioninistr. 15<br>81377 Munich, Germany<br>Phone: +49-89-4400-74491<br>Fax: +49-89-4400-77491
**Monitoring:**	Winicker Norimed GmbH Medizinische Forschung<br>Deutschherrnstraße 15-19<br>90429 Nürnberg, Germany<br>Dr. Markus Hillgärtner<br>Team Leader Clinical Trials<br>Phone: +49 911 92680 – 8767<br>Mobile: +49 173 – 6609626

	Fax: +49 911 92680 – 8840<br>E-Mail: markus.hillgaertner@winicker-norimed.com
**Drug safety / Pharmacovigilance/ SAE Management:**	Winicker Norimed GmbH Medizinische Forschung<br>Deutschherrnstraße 15-19<br>90429 Nürnberg, Germany<br>Dr. Markus Hillgärtner<br>Team Leader Clinical Trials<br>Phone: +49 911 92680 – 8767<br>Mobile: +49 173 – 6609626<br>Fax: +49 911 92680 – 8840<br>E-Mail: markus.hillgaertner@winicker-norimed.com
**Randomization**	Randoulette team of the<br>Institute for Medical Informatics, Biometry and Epidemiology (IBE), LMU Munich<br>Marchioninistr. 15<br>81377 Munich, Germany<br>Phone: +49(0)89-4400-74490/-77484<br>Fax: +49(0)89-4400-77491<br>E-Mail: randoulette@ibe.med.uni-muenchen.de
**Placebo manufacturing:**	Haupt Pharma Wuelfing GmbH<br>Bethelner Landstrasse 18<br>31028 Gronau, Germany<br>Tel: +49/05282-5850<br>Fax: +49/05182-585222<br>Heike Tietze<br>E-Mail: heike.tietze@haupt-pharma.de
**IMP packaging and labeling:**	InPha Sol – Intellignet Pharmaceutical Solutions<br>Pharmacy of Heidelberg University<br>Im Neuenheimer Feld 670

	D-69120 Heidelberg<br>Tel: +49/06221 - 56 6761<br>Fax: +49/06221 - 56 33570<br>Dr. Lenka Taylor<br>E-Mail: taylor.lenka@med.uni-heidelberg.de

5 Introduction

5.1 Background

Cerebellar ataxia is a frequent and often-disabling syndrome severely impairing motor functioning and quality of life.[1] A Japanese survey found a prevalence of all types of cerebellar ataxia of 18.5:100,000.[2] In southeast Norway, the prevalence of hereditary ataxias was 6.5:100,000.[3] A population-based study performed in south east Wales found a prevalence of late onset, non-hereditary ataxia of 8.4:100,000.[4] The studies suggest that the overall prevalence of cerebellar ataxia in Europe is similar to that in Japan and may approach 20:100,000.

According to a recent CONSENSUS paper by some of the leading experts on cerebellar ataxia, no medication has yet been proven effective for the symptomatic or even causative treatment of degenerative cerebellar ataxia.[5] Effects of Varenicline and Riluzole have been reported. However, according to the CONSENSUS paper, the experience of many ataxia clinics is less promising and the findings need to be confirmed in further placebo-controlled trials.[5] Even more crucial to mention is that the trial on Varenicline had an overall dropout rate of 40%, which is most likely due to the considerable side effects of Varenicline. Ultimately, the pre-planned crossover design was therefore not performed. There is unanimous assent that 4-aminopyridine is effective in episodic ataxia type 2 and in downbeat nystagmus syndrome.[6-8] Therefore, the only treatment recommendation is physiotherapy [9] and new therapeutic options are needed.

We reported in a case series on 13 patients with different types of cerebellar ataxia that the modified amino-acid Acetyl-DL-Leucine (5 g per day for one week) significantly improved the symptoms, measured by the Scale for the Assessment and Rating of Ataxia (SARA), the Spinocerebellar Ataxia Functional Index (SCAFI) and EuroQol-5D-5L; the agent was very well tolerated [10]. Mean total SARA decreased remarkably ($p = 0.002$) from a baseline of 16.1 ± 7.1 to 12.8 ± 6.8 (mean ± SD) on medication. There were also significant improvements in sub-scores for gait ($p = 0.022$), speech ($p = 0.007$), finger-chase ($p = 0.042$), nose-finger-test ($p = 0.035$), rapid-alternating-movements ($p = 0.002$) and heel-to-shin-test ($p = 0.018$). Furthermore, patients showed better performance in the SCAFI consisting of the 8-m-walking-time (8 MW, $p = 0.003$), 9-Hole-Peg-Test of the dominant hand (9HPTD, $p = 0.011$) and the PATA rate ($p = 0.005$). Quality of life increased during treatment ($p = 0.003$). No side effects were reported. In conclusion, Acetyl-DL-Leucine significantly improved ataxic symptoms without side effects and therefore showed a good risk-benefit profile. Videos documenting the effects can be found on the webpage of the German Neurological Society (www.dgn.org). The daily dosage of 5 g per day used in this study was in the upper range of the recommended dosages and, as expected from the safety-profile of Acetyl-DL-Leucine, well tolerated.

Although used for more than 50 years, the therapeutic mode of action of Acetyl-DL-Leucine has so far not been very well examined. It may act due to its direct effect on neurons as was shown in the vestibular nuclei [11]. Due to the phylogenetical and electrophysiological similarities and close interactions between vestibular and deep cerebellar neurons [12], we had hypothesized that there may also be a positive effect on ataxic symptoms in cerebellar disorders.

In unilateral labyrinthectomy of guinea pigs, Acetyl-DL-Leucine restores the membrane potential of hyperpolarized/depolarized vestibular neurons [13]. This mechanism is most likely mediated by its direct interactions with membrane phospholipids such as phosphatidylinositol 4,5-bisphosphate, which influences ion channel activity [14]. Thereby, Acetyl-DL-Leucine can stabilize the membrane potential. The input from cerebellar Purkinje cells and mossy/climbing fiber collaterals controls the action potential of the vestibular and the cerebellar nuclei [15], which in turn project to the brainstem, thalamus and spinal cord [12]. Therefore, Acetyl-DL-Leucine may act through afferent and efferent projections on upstream and downstream structures, thus influencing movement control.

Back-translational evidence for the mechanism of action of Acetyl-DL-Leucine

In a previous study by our group, the effect of N-Acetyl-DL-Leucine (TANGANIL®), N-acetyl-L-leucine and N-acetyl-D-leucine on central vestibular compensation following unilateral labyinthectomy (UL) was investigated using behavioural testing and serial [^{18}F]-Fluoro-desoxyglucose ([^{18}F]-FDG)-µPET. A significant reduction of postural imbalance scores was identified on day 7 post UL in the N-Acetyl-DL-Leucine ($p < 0.03$) and the N-acetyl-L-leucine groups ($p < 0.01$), compared to the control group, but not in the N-acetyl-D-leucine group (60 mg/kg body weight, in each group). Measurements of the regional cerebral metabolic rate for glucose (rCGMglc) by means of µPET revealed that N-acetyl-L-leucine but not N-acetyl-D-leucine caused a significant increase of rCGMglc in the vestibulocerebellum and a decrease in the posterolateral thalamus and subthalamic region on days 3 and 7 (Figure 2).[16]

The major findings of this study were the following: 1) N-Acetyl-DL-Leucine accelerates the postural compensation after unilateral vestibular damage; 2) N-acetyl-L-leucine is the pharmacologically active enantiomer that induces this effect; and 3) the potential mechanism of N-acetyl-L-leucine action for improving vestibular compensation consists of an activation of the vestibulocerebellum and a deactivation of the posterolateral thalamus.[16]

Based on these results, further studies are currently being performed to reveal the putative mechanisms of N-Acetyl-DL-Leucine action. So far, it was hypothesized that N-Acetyl-DL-Leucine restores the membrane potential of both hyperpolarized and depolarized vestibular neurons [13]. Our data show an rCMRglc increase in the vestibulocerebellum on days 3 and 7 following N-acetyl-L-leucine application. This may be explained by the close phylogenetic and anatomic similarities and interactions between the vestibular and vestibulocerebellar neurons. N-acetyl-L-leucine, which is a branched-chain amino acid, may modulate glutamate neurotransmission in the cerebellum via the branched-chain amino acid transferases [17-19]. Activation of metabotropic glutamate receptors (mGluR) is required for cerebellar plasticity [20-22]. Currently, the extracellular cerebellar glutamate levels are being measured by in vivo microdialysis after administration of N-Acetyl-DL-Leucine and N-acetyl-L-leucine in the rat model of UL. Furthermore, the interaction of Gabapentin, which is an inhibitor of branched-chain amino acid transferases, and N-Acetyl-DL-Leucine is being investigated. These experiments aim to show that N-Acetyl-DL-Leucine can augment cerebellar plasticity via modulation of glutamatergic mechanisms of motor learning.

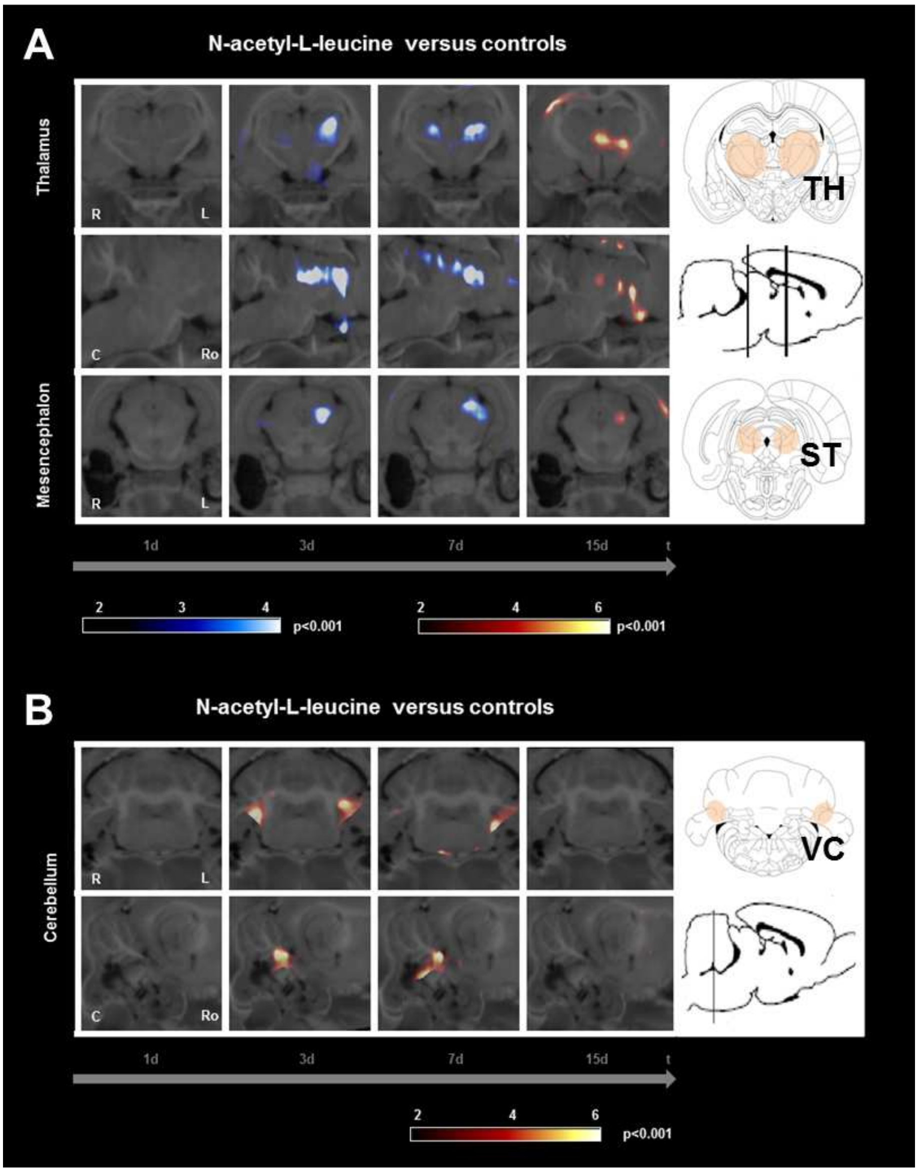

Figure 2: Comparison of µPET images between the N-acetyl-L-leucine group and controls in an animal model of acute unilateral vestibulopathy: A) Compared to controls, the N-acetyl-L-leucine group showed on day 3 after unilateral labyrinthectomy a significant decrease of rCMRglc in the ipsilesional, on day 7 in the ipsilesional > contralesional posterolateral thalamus and subthalamic region. On day 15 an increase of rCMRglc was found in the centromedian thalamus and subthalamic region**. B)** On day 3 rCMRglc was significantly increased in the bilateral, on day 7 in the ipsilesional paraflocculus/ flocculus (vestibulocerebellum). R right, L left, C caudal, Ro rostral, TH posterolateral thalamus, ST subthalamic region, VC vestibulocerebellum

5.2 Trial Rationale

Cerebellar ataxias are most often caused by neurodegenerative disorders of the cerebellum, either hereditary or sporadic. The overall prevalence of cerebellar ataxia is about 20/100,000, i.e., 15,000 patients in Germany (www.ataxie.de).[23] The prevalence of sporadic cerebellar ataxia is 2.2 to 8.4 per 100,000 and that of late onset cerebellar ataxia 10.2 per 100,000 with an annual incidence for the latter of 0.3/100,000[23]; all in all, about 50% of the cases are sporadic.. The symptoms often begin in childhood or adolescence and are generally progressive.[1] Due to the various etiologies and different courses of the diseases, there are no robust epidemiological data on cerebellar ataxia-related mortality.

The leading clinical symptoms of cerebellar ataxia are disturbances of stance/gait (> 85%) with recurrent falls, limb ataxia with severe functional impairment of arm and hand movements, dysarthrophonia with impaired oral communication abilities and ocular motor disturbances with impaired vision.[1] In a European multi-center study patient-reported health status was severely compromised (mean EQ-5D visual analogue scale 61.5). Specifically, problems were reported in the dimensions of mobility (86.9% of patients), usual activities (68%), pain/discomfort (49.4%), depression/anxiety (46.4%), and self-care (38.2%); multivariate analysis revealed 3 independent predictors of subjective health status: ataxia severity, extent of noncerebellar involvement, and the presence of depressive syndrome.[24] Further, most types of cerebellar ataxia are progressive and therefore become more disabling in the course of the disease, severely impairing quality of life and functioning.[1] In addition to functional impairment, cerebellar ataxia also affects cognitive and psychosocial abilities and limits the ability to perform tasks of daily life. It is thus a severely disabling condition with a major impact on quality of life, progressively restricting autonomy and social participation. Required caregiving activities are frequently provided by the family, thus further increasing the socioeconomic burden of disease.[25] Major cost components relevant for patients with cerebellar ataxia are informal care, early retirement because of permanent disability, drugs, orthopaedic devices, and rehabilitation. With progressing disease QoL decreases and utilization of health resources increases.[26]

No medication has yet been proven effective for the symptomatic or even causative treatment of degenerative cerebellar ataxia.[5] So far, the only treatment recommendation is physiotherapy [9] and new therapeutic options are needed. The goal of this study is to demonstrate that Acetyl-DL-Leucine is efficacious in improving motor function and quality of life in patients with cerebellar ataxia offering a good benefit-risk profile.

5.3 Side effects and Risk Benefit Assessment

5.3.1 Side effects

Acetyl-DL-Leucine (TANGANIL®) benefits from a large usage (for more than 40 years) for adult patients without any major side effect or dependence phenomenon in different causes of vertigo and/or dizziness.

All available data concern the racemate, Acetyl-DL-Leucine, the active substance of TANGANIL®.

From the post-marketing experience, the safety of TANGANIL® has been reviewed in a number of Periodic Updated Safety Reports (PSUR), the most recent of which covers the period from January 1st 2001 to December 31st 2005.

In this PSUR, patient exposure was estimated to be equal to 6 390 461 patients treated with TANGANIL® 500 mg tablet and 812 767 patients treated with TANGANIL® 500 mg/5 mL ampoule for IV injection, with 506 124 570 tablets and 5 981 970 ampoules distributed during this period. Overall, 81 French spontaneous case reports were received, with 52 considered as non-serious. All of the 81 case reports were unexpected according to the current SmPC.

The relation with TANGANIL® treatment (according to the French imputability method) has been evaluated as follows:

1 case is attributed as highly probable: arthralgia with muscle weakness (1 non serious),

7 cases are attributed as probable: mainly erythema, exanthema, rash, pruritus, urticaria, pain in the injection site when using TANGANIL® intravenous (2 serious, 5 non serious),

7 cases are attributed as possible (3 serious, 4 non serious),

47 cases are attributed as doubtful (24 serious, 23 non serious),

19 cases with unknown imputability or not applicable (in which 13 cases concern a TANGANIL® exposure during pregnancy) (19 non serious).

Considering the first reported effects, the organ systems the most frequently concerned are:

10 cases: gastrointestinal system disorders: (miscellaneous with no systematic character)

13 cases: central and peripheral nervous system disorders

11 cases: skin and appendage disorders

Skin reactions ranging from pruritus to AGEP (acute generalised exematous pustulosis) were reported. In two cases of erythema with pruritus, the causality assessment was considered as probable. In their majority, reported events were doubtfully related to product and occurred in a context of concomitant treatment with known skin reaction.

7 cases: overall conditions.

No overdosage, or abuse or drug dependence induced by TANGANIL® exposure has been reported.

The analysis of the observations reported does not show any new adverse effect related to the TANGANIL® exposure except possible cutaneous reactions that are heterogeneous and non-specific and reaction on the injection sites for the intravenous formulation.

To sum up, during the period covered by the PSUR, no increase in incidence or intensity of reported reactions was observed. However, relevant safety findings were identified and a change to the SmPC was proposed.

This variation to the SmPC has been approved by Health Authorities in February 2007 with the main following additions:

A contraindication for known hypersensitivity to Acetyl-DL-Leucine or one of the excipients.

In very rare cases, skin rash (sometimes associated with pruritus), urticaria.

In conclusion, the safety assessment allows maintaining the favourable risk/benefit ratio for TANGANIL®.

<u>Very frequent (> 10%) side effects:</u>

Not known

<u>Frequent (1-10%) side effects:</u>

Not known

<u>Occasionally (0.1 – 1%) side effects:</u>

Not known

<u>Rare side effects (0.01 – 0.1%):</u>

- Skin rash (sometimes associated with pruritus)
- Gastrointestinal system disorders: (miscellaneous with no systematic character)

5.3.2 Risk Benefit Assessment

Acetyl-DL-Leucine is currently not approved in Germany. However, since it has been an over the counter drug in France for over 50 years without serious side effects, the risk for patients is considered low. Known contraindications for Acetyl-DL-Leucine also apply for this study. Except for the blood tests, all tests and assessments applied are non-invasive, and also performed in clinical routine. On the other hand, the potential benefit may be substantial for cerebellar ataxia patients.

Acetyl-DL-Leucine could be the first medical symptomatic treatment option, possibly resulting in enhanced motor functioning.

Patients will be included in the study after detailed informed consent. Only standard diagnostic examinations will be used during the trial; no experimental examinations are planned. To minimize risks, patients will receive the best clinical treatment with continuous monitoring of adverse events and side effects including blood tests. The patients' confidentiality will be protected by pseudonymization.

6 Trial Objectives and Endpoints

The study is designed as a multicenter, randomized, double-blind, placebo-controlled, 2-treatment 2-period crossover phase III trial. The overall goal is to evaluate whether Acetyl-DL-Leucine treatment improves clinical ataxia scores, as well as a quality of life, and the two common comorbidities depression and fatigue in participants with cerebellar ataxia. The study aims to describe an efficacy and safety profile for experimental treatment with Acetyl-DL-Leucine compared to placebo intervention.

6.1 Primary Objective

The primary aim of the ALCAT trial is to determine the effect of Acetyl-DL-Leucine compared to placebo intervention on improving motor function measured by the total score of the Scale for the Assessment and Rating of Ataxia (SARA).

6.2 Primary Efficacy Endpoint

The primary efficacy endpoint is defined as the absolute changes in SARA total score from period-level baseline to the end of the 6-weeks treatment period (verum/ placebo), i.e. the difference between post-treatment values and the corresponding period-level baseline values:

Delta (SARAtotal) = SARAtotal (post-treatment) – SARAtotal (period baseline)

6.3 Secondary Objectives

To assess the efficacy of Acetyl-DL-Leucine compared to placebo as regards

a) improving motor function measured by the Spinocerebellar Ataxia Functional Index (SCAFI) and SARA subscore items,

b) quality of life (QoL, EQ-5D-5L) as key secondary objective, and depression (BDI-II) and fatique (FSS) as further secondary objectives.

To check for the occurrence of the adverse effects reported in the summary of the medical product characteristics (SmPC) of the drug.

6.4 Secondary Efficacy endpoint

Absolute changes in the

- subscores of SARA,
- SCAFI total score and subscores of SCAFI

as well as patient-reported outcomes

- EQ-5D-5L (visual analogue scale; descriptive system),
- Beck's Depression Inventory (BDI-II),
- and the Fatigue Severity Scale (FSS)

from (period-level) baseline to the 6-week visit at the end of the verum/ placebo treatment period. Additionally, change scores will be assessed at the follow-up visit 8.

6.5 Safety Variables

To investigate the occurrence of side effects (AE, SAE, SUSAR) within both treatment periods and at the end of the post-treatment follow-up period.

7 Trial Design

7.1 Trial Design

The study is designed as a multinational, multicenter, prospective, randomized, double-blind, placebo-controlled, 2- period 2-treatment crossover phase III superiority trial with a fixed sample design.

7.2 Intervention scheme

Study participants will be randomly assigned to one of two different treatment sequences, either Acetyl-DL-Leucine followed by placebo or vice versa.

Each sequence consists of a first 6 weeks (42 days) treatment period, a 4 weeks (28 days) wash-out period, a second 6 weeks (42 days) treatment period.

Finally, a post-treatment follow-up is scheduled 4 weeks (28 days) after the second treatment period (see Schedule of activities 2).

Figure 1 ALCAT trial: intervention scheme with study visits

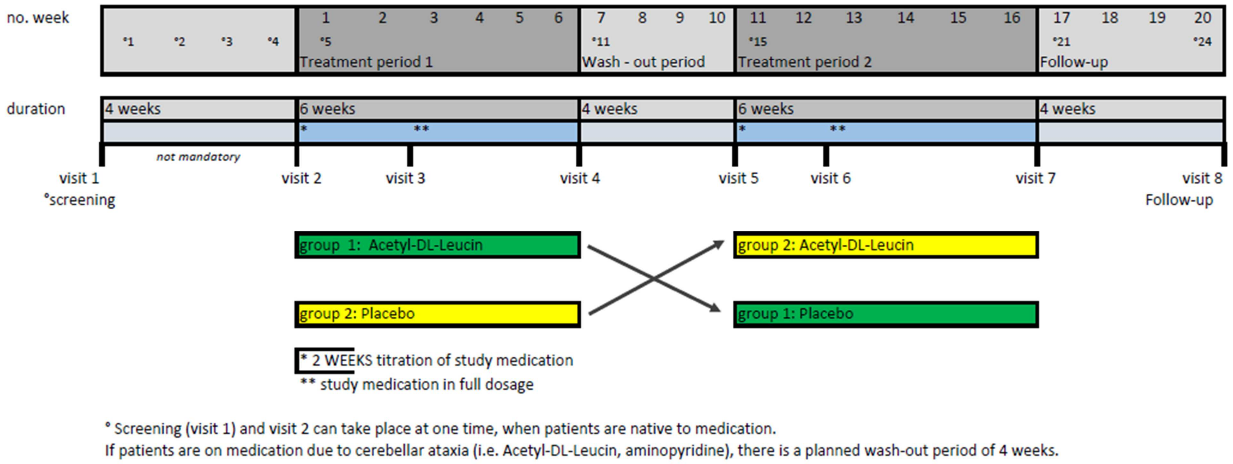

For the trial schedule please refer to *Schedule of Activities* (see section 2)

7.3 Number of centers

The trial will be conducted in approximately 7 centers in Germany and other EU countries, which must meet the structural and personnel requirements for performing the planned regular trial-related investigations.

If necessary, additional centers may be included in the trial.

7.4 Number of patients

To be randomized: n = 108 patients

To be analyzed: n = 86 patients (expected to complete both phases)

7.5 Time Schedule

Per patient:

- duration of treatment: 2 x 6 weeks
- duration of wash-out: 4 weeks
- duration of follow-up: 4 weeks
- total individual study duration: 20 weeks (+4 weeks if a wash-out prior to randomization is necessary)

Trial duration:

- Recruitment period (FPFV to LPFV) (months): 16
- Planned Start Date (FPFV): 01.12.2015
- Planned End Date (LPLV): 01.08.2017
- First patient in to last patient out (months): 20
- Duration of the entire trial (months): 32

The end of the clinical trial is defined by the last individual trial-specific examination during the last visit of the last subject.

8 Trial Population and Eligibility Criteria

This trial can fulfill its objectives only, if appropriate subjects are enrolled. The following eligibility criteria are designed to select subjects for whom protocol treatment is considered appropriate. All relevant medical and non-medical conditions should be taken into consideration when deciding whether this protocol is suitable for a particular subject.

8.1 Gender Distribution

No gender ratio has been stipulated in this trial as the results of preclinical and / or clinical studies or medical literature did not indicate any difference in the effect of the trial treatment in terms of efficacy and safety.

8.2 Inclusion Criteria

Subjects will only be included in the study if they meet all of the following inclusion criteria so that they are eligible for enrollment into the trial:

- Clinically confirmed cerebellar ataxia (CA) with a total SARA-Score ≥ 3 (range 0-40) of hereditary or non-hereditary degenerative type
- Patient did not receive any of the following prohibited medication within 4 weeks prior to randomization:
    - Aminopyridines
    - Acetyl-DL-Leucine
    - Riluzole
    - Gabapentin
    - Varenicline
    - Chlorzoxazone
- The ability to follow study instructions and likely to attend and complete all required visits Written informed consent of the subject prior to any study specific intervention
- Age ≥ 18 years

8.3 Exclusion Criteria

Subjects will not be included in the study if any of the following criteria applies:

- Subjects is not able to give consent
- Onset of ataxia in association with stroke, encephalitis, sepsis, hyperthermia or heat stroke

- Toxic causes for ataxia of cerebellar type
- Rapid progression of ataxia (development of severe ataxia in less than 12 weeks)
- Subject suffers from any of the following:
    - chronic diarrhea
    - unexplained visual loss
    - malignancies
    - insulin-dependent diabetes mellitus
- Ataxia due to multiple sclerosis, ischemia, hemorrhage or tumor of the posterior fossa as confirmed by imaging
- Ataxia due to clinical likely multisystem atrophy type C (MSA-C)
- Diagnosis of clinical likely Friedreich ataxia
- Known history of hypersensitivity to the investigational drug or derivates
- Liver failure defined as AST/ALT > 300 U/l
- Simultaneous participation in another clinical trial or participation in any clinical trial involving administration of an investigational medical product within 30 days prior to the beginning of the clinical trial
- Subjects with a physical or psychiatric condition which at the investigator’s discretion may put the subject at risk, may confound the trial results, or may interfere with the subject’s participation in this clinical trial
- Known or persistent abuse of medication, drugs or alcohol
- Females of childbearing potential, who are not using and not willing to use medically reliable methods of contraception for the entire study duration as listed in the patient informed consent form
- Current or planned pregnancy or nursing women
- Patient has received any of the following prohibited medication within 4 weeks prior to randomization
    - Aminopyridines (including substained-release form)
    - Acetyl-DL-Leucine
    - Riluzole
    - Gabapentin
    - Varenicline
    - Chlorzoxazone

The indication specific exclusion criteria are chosen based on the available data on Acetyl-DL-Leucine of the French Agence nationale de sécurité du médicament et des produits de santé (http://agence-prd.ansm.sante.fr/php/ecodex/notice/N0126720.htm, visited at 24.04.2015, dated on 20.02.2007), in particular hypersensitivity to the agent.

8.4 Subject Information and Recruitment

If a subject appears to be eligible for the trial, the investigator will inform the subject about the trial and ask the subject or in case of subjects without legal capacity the legal representative/authorized agent for his/her written consent.

It is a requirement that written consent is obtained prior to any trial-specific procedure.

8.5 Randomization and allocation concealment

All patients who give consent for participation and who fulfill the inclusion criteria will be randomized.

Randomization is requested by the staff members responsible for recruitment at the trial centers.

The randomization number consists of a consecutive three-digit tracking number (so called package number). The patient identification number results from a combination of the trial center number and the package number.

Concealed randomization to both treatment sequences (Verum->Placebo; Placebo->Verum) will be performed with a 1:1 allocation ratio. The allocation sequence will be generated by an independent person of the Institute for Medical Informatics, Biometry and Epidemiology (IBE) of the University of Munich not involved in assessing the outcomes of the study. Neither the investigators nor the patients will be informed about the treatment sequences to which a patient is allocated, and neither can get access to the randomization list. The randomization is based on a permuted balanced block design with random block length. The procedure considers stratification by center and by 'genetically' vs. 'sporadic' cerebellar ataxia.

According to this pre-specified randomization list provided by the IBE the trial medication kits will be signed out with (consecutive) identification numbers. An opaque sealed envelope containing the corresponding treatment sequence for unblinding in the case of an emergency will be attached. Hence, the investigator can unblind a single patient at any time. All envelopes have to be sent back to the DSGZ/sponsor-delegated person at the end of the trial. Each trial center receives a pool of trial kits. The package numbers for the kits stored at each center will be registered at the IBE. The IBE will provide the internet-based randomization tool "Randoulette" (https://wwwapp.ibe.med.uni-muenchen.de/randoulette/) which assigns one of the trial kits stored at the respective center when a new patient fulfils the inclusion criteria. In this way, an immediate registration and randomization of enrolled participants is guaranteed. Furthermore, the amount of trial kits stored at the centers can be checked continuously. Replacement of the trial kit is only possible after contact and organization with the Study Center of the DSGZ and the Randoulette team.

9 Investigational Medicinal Product (IMP)

9.1 Specification of IMP

IMP 1*: Acetyl-DL-Leucine (TANGANIL®)*

**Drug name**	TANGANIL®
**Name of manufacturer**	Pierre Fabre, Castres, France
**Substance name**	Acetyl-DL-Leucine
**Dose of active ingredient per unit**	500 mg
**Other ingredients**	Wheat starch, pregelatinized corn starch, calcium carbonate, magnesium stearate
**Pharmaceutical form**	Tablets
**Mode of administration**	Oral
**Storage conditions**	Do not store > 25℃.<br>The drug tablets will be reblistered from original blisters and packaging boxes and relabeled. All procedures will be performed under sterile conditions. All procedures will be performed the pharmacy of the University of Heidelberg.

IMP 1 is already used as a marketed product (TANGANIL®) in France, Congo, Tansania and Vietnam. The IMP 1 will be purchased from Pierre-Fabre and then delivered and supplied to the trial centers by the Pharmacy of Heidelberg University – together with the corresponding placebo. The TANGANIL® tablets will be refilled from the original packaging to blister under sterile conditions and study specific labels applied, these blisters are in a pack. These procedures will be performed by the Pharmacy of Heidelberg University.

IMP 2*: Corresponding Placebo*

**Drug name**	Placebo
**Name of manufacturer**	Haupt Pharma Wuelfing GmbH
**Substance name**	NA
**Dose of active ingredient per unit**	NA
**Other ingredients**	Wheat starch, pregelatinized corn starch, calcium carbonate, magnesium stearate
**Pharmaceutical form**	Tablets
**Mode of administration**	Oral
**Storage conditions**	Do not store > 25℃.

Placebo will be an identically appearing tablet and visually indistinguishable from TANGANIL®. Placebo bulkware will be manufactured and released by Haupt Pharma Wuelfing GmbH and send to the Pharmacy of Heidelberg University for study specific primary and secondary packaging.

9.2 Packaging and Labelling of IMP

The carton box with the IMP will be labeled as follows (according to Annex 13, GMP):

- Name, address and telephone number of sponsor
- Name, address and telephone number of manufacturing / packaging pharmacy
- Name of the drug: Acetyl-DL-Leucine/Placebo
- Other Ingredients
- mode of application, dosage form, quantity of dosage units, strength
- Charge identification (Ch.-B.)
- Package number
- Treatment phase
- Protocol Code ALCAT
- EudraCT No. 2015-000460-34
- Expiry date
- Instructions for storage: do not store > 25 °C
- Note: For clinical trial use only
- Note: keep out of reach of children

The blister will be labeled with the minimum particulars according to Annex 13, GMP. The labeling will be in German and in Dutch.

9.3 Transport of IMP

Distribution and shipment of Acetyl-DL-Leucine and the corresponding placebo to the trial centers will be organized by the Pharmacy of Heidelberg University. A temperature control during transport is not necessary.

9.4 Storage requirements

The following storage conditions have to be kept for the IMP(s):

Acetyl-DL-Leucine (TANGANIL®) and Placebo: Do not store > 25℃

The investigator is responsible for ensuring adequate storage and sufficient stock supply of the IMP(s) at the center in a secure location at the site, preferably in a lockable cabinet with restricted access to the investigator(s) and authorized site staff. Personnel who have access to the trial drug need to be listed (name and responsibilities) on the Team Delegation Log in the trial specific Investigator Site File (ISF).

The investigator should ensure that the IMP is only used according to the protocol.

Temperature has to be recorded continuously using a Min-Max-Thermometer. These recordings have to be controlled weekly, the results have to be documented in a temperature log by an authorized person.

9.5 Dosage, Mode of Application and Dose Schedule

• Strength:	500 mg Acetyl-DL-Leucine/ Placebo		
• Mode of application:	oral intake		
• Duration of treatment:	6 weeks		
• Dose schedule	Week 1	1.5 g per day	1-1-1
	Week 2	3 g per day	2-2-2
	Week 3-6	5 g per day	3-3-4

The primary packing of the study medication are blisters, the secondary packaging a carton box – one for each treatment phase.

<u>The medication has to be taken at least 30 minutes before and at least 2 hours after a meal.</u>

9.6 Handling of IMP at the Site and Drug Accountability

The packaged IMP(s) provided by the Pharmacy of Heidelberg University has to be stored in a secure location at the site, preferably in a lockable cabinet with restricted access to the investigator(s) and authorized site staff. Personnel who have access to the trial drug need to be listed (name and responsibilities) on the Team Delegation Log in the ISF. In accordance with all applicable regulation requirements, the investigator is responsible for the IMP / randomized therapy accountability, reconciliation, and record maintenance on appropriate forms. The investigator is responsible that the complete and intact receipt of the total quantity of the IMP is ensured and documented as well as the correct storage of the IMP (see also section 9.4).

The investigator should ensure that the IMP is only used according to the protocol, and may only dispense the IMP to subjects who have signed the informed consent and who have been randomized in the trial. The dispensing of the investigational medicinal product to subjects outside of this clinical subject is not permitted.

The monitor will check the complete documentation during the regular monitoring visits and will clarify potential discrepancies between usage and dispensing of the IMP(s). The investigator or the monitor has to inform the sponsor-delegated person or the coordinating investigator in case of deficiency regarding e.g. storage or accountability of the IMP.

9.7 Procedures for Monitoring the Subject Compliance

Subjects are asked to take their trial drugs (used/empty and unused blister) with them at visit 3, 4 (first treatment period) and at visit 6, 7 (second treatment period). Unused medication has to be returned at visit 4 and visit 7. By counting the remaining tablets, treatment compliance with can be assessed. IMP dispense and return will be documented on the Drug Accountability Log to be filed in the ISF (copies should be send to the DSGZ in regular intervals). In the case of treatment discontinuation, the date of last intake and - if applicable - time periods without treatment will be recorded in the CRF.

9.8 Return and Disposal of IMP

The subject has to return the IMP at Visit 4 and Visit 7. All unused IMP is going to be destroyed on site. Destruction of IMP has to be documented on the Drug Accountability Log or the Final Site Drug Return and Destruction Log.

Copies of the forms completed at the trial site will be returned to the sponsor delegated person or the coordinating investigator at the end of the trial, and will be collected by the monitor during the close-out visit or sent to the sponsor-delegated person or the coordinating investigator on request. The original will remain on site.

9.9 Blinding and Emergency Codes

The trial will be subject and investigator blinded. Study personnel involved in patient recruitment and trial activities will have no access to the randomization list provided by the IBE. The statistician responsible for the final analysis will also have no access to the list.

9.10 Unblinding (Code break)

At the initiation of the trial, the trial site will be instructed on the method for breaking the blind (see 8.5). The actual allocation must not be disclosed to the patient and/or other study personnel including other site personnel, monitors, corporate sponsors or project office staff; nor should there be any written or verbal disclosure of the code in any of the corresponding patient documents.

Early/ premature unblinding:

In general, breaking the blind should be considered only when knowledge of the treatment assignment is deemed essential for the study patient’s care by the participant’s physician or regulatory body. Unblinding should be restricted to emergency situations for reasons of subject safety, and is expected to be extremely rare in this trial. Unblinding should not necessarily be a reason for study discontinuation.

Where possible, it is important that all other members of the study team (e.g. other investigators, statisticians) remain blinded.

When the blinding code is broken the reasons must be recorded and documented on the case report form. Any unblinding will be documented in the study report.

Any of the following situations may be reasons for premature unblinding:

- In emergency situations, if it is necessary for the subject's safety, i.e. if the further treatment depends on the knowledge of the investigational medicinal product.
- In the event of accidental administration of the investigational medicinal product to a person who is not a subject.
- In the event of the death of a subject, if a causal relationship between the treatment with the investigational medicinal product and death is suspected.
- In the event of SAEs / SUSARs under certain conditions (causal relationship with the investigational medicinal product).

The decision whether unblinding is necessary lies with the investigator.

Regular unblinding:

As a matter of principle, unblinding happens regularly when the trial database is closed, after blinded data review following resolution of queries.

9.11 Prior and Concomitant care and intervention

9.11.1 Previous therapy / medication of trial specific illness

All previous therapies / medications applied over a period of 12 months before visit 1 will be documented in the subject's medical record and in the appropriate CRF.

9.11.2 Previous therapy / medication of other indications than the trial specific illness

All previous treatments and medications for other indications than the trial specific illness occurred within the last 3 months before visit 1 of each patient will be documented in the CRF.

9.11.3 Prohibited concomitant therapy / medication for trial specific illness

All other medication, which has been previously described to have a potential positive effect on cerebellar ataxia, in particular 4-aminopyridine, Riluzole, Varenicline, Gabapentin or Chlorzoxazone as mentioned in the exclusion criteria are not allowed to be applied during the trial.

Every effort should be made to avoid the use of any of the above listed prohibited therapies/medications during the entire duration of the trial and 4 weeks prior to treatment start.

If there is a single administration of any of the drugs mentioned above, the subject is still eligible for the main analysis. Regular intake of these drugs is considered a protocol violation (see protocol violations 13.5).

Role of physiotherapy and speech therapy

Physiotherapy as well as speech therapy should not be started new while the subject is enrolled in the trial. If a patient is on physiotherapy and/or speech therapy 6 months prior to randomization, the amount (therapy hours per week) needs to be documented in the subjects' medical record and in the CRF and has to remain unchanged during the individual trial duration.

9.11.4 Concomitant therapy / medication for other indications

All concomitant therapies/medications other than the trial therapy/investigational medicinal product applied during the trial at the discretion of the investigator will be documented in the patient's records and will be documented in the CRF.

The doses of concomitant medications for e.g. chronic diseases should be kept as constant as possible throughout the trial. Concomitant medication will be recorded during whole individual study duration.

10 Trial Procedures

10.1 Methods of Assessment

The following section will give an overview and adequate explanations to the examinations and procedures to be performed in this trial. Source documents, including radiological imaging, if applicable, must be stored and be available for subsequent review. The respective printouts will be stored in the subject medical file.

10.1.1 Scores for ataxia

The clinical severity of ataxia is assessed by different clinical scores. Specifically, the following two validated scores will be used:

- Scale for the assessment and rating of ataxia (SARA) [27]
- Spinocerebellar ataxia functional index (SCAFI) [28, 29]

Both scores assess ataxia of stance and gait, including gait speed as well as limb ataxia and dysarthria. Both scales are widely used in clinical practice [29] and treatment trials on cerebellar ataxia.[30, 31] Both scores cover the whole range of impaired motor function in ataxic patients.

The SARA serves as a key inclusion criterion for the severity of ataxia at the beginning of the study prior to enrollment. The SARA total score consists of 8 items reflecting neurologic manifestations of cerebellar ataxia. Each item represents a vital movement feature in cerebellar ataxia rated by an experimenter (gait, stance, sitting, speech disturbance, finger chase, nose-finger test, fast alternating hand movements, heel-shin slide) with a range of 4 – 8 points on each item resulting in a total score of 0 – 40 points. It is a reliable and valid clinical scale with a high internal consistency that measures the severity of ataxia and increases with ataxia disease stage.

Another clinical ataxia score, the SCAFI uses a timed approach evaluating the eight-meter-walking-time (8MW), the nine-hole-peg-test (9HPT) testing limb ataxia and the PATA-test for speech.

Equivalent to the SARA, the SCAFI also represents vital movement features (8 meter walking test; 9-hole-peg-test for evaluation of the coordination of the upper extremities; PATA-test for evaluation of speech). In contrast to the SARA, the SCAFI uses a timed approach rated by an experimenter. After the assessment, raw scores are transformed into reciprocals and converted into subtest Z-scores. The final SCAFI is then generated as the arithmetic mean of all three Z-scores. The scores will be measured at each visit.

The scores will be assessed by the investigators (see also Appendix section 22.1 and section 22.2).

10.1.2 Questionnaires for quality of life, depression and fatigue

Furthermore, the following self-administered questionnaire for evaluating the quality of life (QoL) will be used:

- EuroQuol-5D-DL [32]

(multiple choice questionnaire and a visual analogue scale) and takes only few minutes to complete. The EQ-5D-5L will be assessed at every visit.

The EQ-5D-5L is a standardized measure of health status and in order to provide a simple, generic measure of health for clinical and economic appraisal and consists of 2 parts - the *EQ-5D descriptive system* and the *EQ visual analogue scale (EQ VAS)*. The EQ-5D-5L descriptive system comprises the following 5 dimensions: mobility, self-care, usual activities, pain/discomfort and anxiety/depression.

Each dimension has 5 levels: no problems, slight problems, moderate problems, severe problems, and extreme problems.

As depression and fatigue are known common comorbidities in patients with cerebellar ataxia, two additional questionnaires will be used:

- Beck depression inventory (BDI-II) [24, 33, 34]

  The Beck's Depression Inventory (BDI-II) is a multiple choice self-report inventory for measuring the severity of depression and is composed of items relating to symptoms of depression such as hopelessness and irritability, emotions such as guilt or feelings of being punished, as well as physical symptoms such as fatigue, weight loss and lack of interest in sex.

- Fatigue severity score (FSS) [34]

  The Fatigue Severity Scale (FSS) captures the patient's experience of mental or psychological fatigue and how it interferes with performing certain activities (exercise, work and family life). It is a self-reporting scale using 9 items. The mean of all answered items represents the fatigue severity..

These questionnaires will be completed by the patients themselves. See also Appendix 22.3, 22.4 and 22.5.

10.1.3 Laboratory examinations / Biological Specimens

A routine blood-sample will be taken to exclude liver or kidney failure and a pregnancy test for women of childbearing potential. A pregnancy test is not required for postmenopausal (amenorrhea > 12 months), surgically sterilized or hysterectomized women. If a routine blood sample examination not older than 7 days prior to visit 1 is available, no renewed blood testing needs to be done. Blood sample examinations older than 7 days but not older than 30 days prior to visit 1 are considered a minor protocol violation.

The following parameters will be measured according to the time schedule given in section 2 (schedule of activities):

- Sodium
- Potassium
- Creatinine
- Serum bilirubin level
- AST
- ALT
- Urea
- ALP
- TSH
- Hemoglobin
- Erythrocytes
- Hematocrit
- Thrombocytes
- Leukocytes
- Pregnancy test for women of child bearing potential

The total amount of blood taken per subject during the entire trial will be approximately 20 ml. At each study center, the blood testing will be done by the in-house laboratory.

The results from the blood test will be reviewed and evaluated by one of the investigators of each study center in a timely manner. Clinically significant findings at screening visit 1 which describe the baseline status of the subjects will be documented as concomitant disease under medical history. In case of meeting any exclusion criteria, the patient cannot be included in the study.

10.2 Time schedule of Measurements

See Schedules of activities and assessments (section 2).

10.2.1 Screening period

Written informed consent will be obtained from all subjects before performing the first (study specific) screening procedure.

All subjects will be screened for inclusion and exclusion criteria before randomization. Only subjects meeting the inclusion and none of the exclusion criteria will be allocated to the trial.

Screening visit/ visit 1

- Check of inclusion/exclusion criteria
- Blood test including pregnancy test for women of child bearing potential
- Patient characteristics and medical history as well as medical history concerning the trial specific illness, including frequency of physiotherapy and speech therapy (documented in hours per week)
- Drug history including documentation of prior therapies/medications and concomitant medications for treatment of trial specific illness as well as drug history for other indications
- Documentation of concomitant medication
- Neurological examination
- Ataxia rating scale (SARA)

Patients on prohibited medication (i.e. medication for cerebellar ataxia like 4-Aminopyridine retarded, Acetyl-DL-Leucine etc.) at screening visit/ visit 1 have to wash-out these medications for 4 weeks and then continue with visit 2 (randomization and start of trial medication), resulting in a total study duration of 24 weeks.

10.2.2 Treatment period 1

The treatment period 1 will include 3 visits from week one until week 6.

A delay of -3 and +5 days is acceptable for visit 2, for all other visits a delay of ± 5 days is acceptable.

All visits will be performed according to the Schedule of Activities and Assessments (see Schedule of Activities and Assessments 2).

<u>Visit 2</u>

- Randomization
- Dispensing of trial drug
- Ataxia rating scales (SARA, SCAFI)
- Quality of life questionnaires (EQ-5D-5L, BDI-II, FSS)
- Documentation of adverse events
- Documentation of frequency of physiotherapy and speech therapy (hours per week)
- Documentation of concomitant medication

If patients aren't on any medication due to cerebellar ataxia, visit 2 and visit 1 can take place together. If visit 1 and visit 2 take place at the same date, ataxia rating scale (SARA) as well as documentation of adverse events and documentation of frequency of physiotherapy and speech therapy are assessed only once. Baseline level is defined as measurements taken at visit 2.

<u>Visit 3 and visit 4</u>

- Ataxia rating scales (SARA, SCAFI)
- Self-administered questionnaires (EQ-5D-5L, BDI-II, FSS)
- Documentation of adverse events
- Documentation of frequency of physiotherapy and speech therapy (hours per week)
- Documentation of concomitant medication
- Blood test including pregnancy test for women of child bearing potential (at visit 4only)
- Return of trial drug at visit 4
- Compliance check

Visit 3 takes places after two weeks on medication, Visit 4 after six weeks on medication.

10.2.3 Wash out

A wash out period of 4 weeks will be performed between the two treatment periods (between visit 4 and 5)

10.2.4 Treatment period 2

The treatment period 2 will start after a wash out period of 4 weeks (between visit 4 and visit 5) and will include 3 visits from week 10 to week 16.

A delay of -3 and +5 days is acceptable for visit 5, for all other visits a delay of ± 5 days is acceptable.

All visits will be performed according to the Schedule of Activities and Assessments (see 2).

<u>Visit 5</u>

- Dispensing of trial drug
- Ataxia rating scales (SARA, SCAFI)
- Self-administered questionnaires (EQ-5D-5L, BDI-II, FSS)
- Documentation of adverse events
- Documentation of frequency of physiotherapy and speech therapy (hours per week)
- Documentation of concomitant medication
- Blood test including pregnancy test for women of child bearing potential pregnancy test for women of child bearing potential

Visit 5 takes place after the wash-out period of 4 weeks.

<u>Visit 6 and visit 7</u>

- Ataxia rating scales (SARA, SCAFI)
- Self-administered questionnaires (EQ-5D-5L, BDI-II, FSS)
- Documentation of adverse events
- Documentation of frequency of physiotherapy and speech therapy (hours per week)
- Documentation of concomitant medication
- Blood test including pregnancy test for women of child bearing potential (only at visit 7)
- Documentation of concomitant medication
- Return of trial drug at visit 7
- Compliance check

Visit 6 takes place after two weeks on medication, visit 7 after 6 weeks on medication.

10.2.5 Follow-up period

The follow-up period takes place after treatment period 2 and lasts for 4 weeks. The follow up ends with visit 8 which is the final visit of the study. A delay of ± 5 days is acceptable.

<u>Final Visit 8 (Close-out visit)</u>

- Ataxia rating scales (SARA, SCAFI)
- Documentation of frequency of physiotherapy and speech therapy (hours per week)
- Self-administered questionnaires (EQ-5D-5L, BDI-II, FSS)
- Documentation of adverse events
- Documentation of concomitant medication

10.2.6 Premature termination of trial / drop-out visit

The following procedures will be performed for subjects who withdraw from the trial and will terminate the trial prematurely. The premature termination visit (“drop-out visit”) is scheduled 4 weeks after taking the last dose of study medication.

If a patient prematurely terminates treatment period 1, an immediate start of treatment period 2 after a wash-out period of 4 weeks is possible. In this case no premature termination visit should be conducted. The trial will be continued with treatment period 2 at visit 5.
If a patient prematurely terminates treatment period 2, the premature termination visit has to take place.

<u>Drop-out visit:</u>

- Ataxia rating scales (SARA, SCAFI)
- Self-administered questionnaires (EQ-5D-5L, BDI-II, FSS)
- Documentation of adverse events
- Documentation of frequency of physiotherapy and speech therapy (hours per week)
- Documentation of concomitant medication
- Blood test including pregnancy test for women of child bearing potential

11 Safety Data Collection, Recording and Reporting

11.1 Definitions

Adverse Event (AE)

An Adverse Event/Experience (AE) is any untoward medical occurrence in a patient or in a clinical investigation subject administered a pharmaceutical product and which does not necessarily have a causal relationship with this treatment.

An AE can therefore be any unfavorable and unintended sign (including an abnormal laboratory finding), symptom or disease temporally associated with the use of a medicinal product, whether or not related to the treatment.

Adverse (Drug) Reaction (AR)

In the pre-approval clinical experience with a new medicinal product or its new usages, particularly as the therapeutic dose(s) may not be established, all noxious and unintended responses to a medicinal product related to any dose should be considered adverse drug reactions. The phrase responses to a medicinal product means that a causal relationship between a medicinal product and an adverse event is at least a reasonable possibility, i.e. the relationship cannot be ruled out.

Regarding marketed medicinal products: a response to a drug which is noxious and unintended and which occurs at doses normally used in man for prophylaxis, diagnosis, or therapy of diseases or for modification of physiological function

Unexpected Adverse (Drug) Reaction (UAR)

An adverse reaction, the nature or severity of which is not consistent with the applicable product information (e.g., Investigator's Brochure for an unapproved investigational product or package insert/Summary of Product Characteristics for an approved product)

Serious Adverse Event (SAE)

Any untoward medical occurrence that at any dose:

- results in death
- is life-threatening
- requires inpatient hospitalization or prolongation of existing hospitalization
- results in persistent or significant disability/incapacity or
- is a congenital anomaly/birth defect
- is qualified as another medically significant event or condition

Serious Adverse (Drug) Reaction (SAR)

This is defined as an adverse drug reaction that is serious (see SAE criteria above) and at least possibly related to the IMP.

Suspected Unexpected Serious Adverse (Drug) Reaction (SUSAR)

A SUSAR is an adverse reaction, which is suspected, serious and unexpected because the nature or severity of this event is not consistent with the applicable product information (e.g. Summary of Product Characteristics for an authorized product or Investigator’s Brochures for an unauthorized investigational medicinal product).

See also section Assessment of Seriousness (11.2.2).

11.2 Criteria to be evaluated by the investigator (1st assessment)

Special attention is to be paid to the occurrence of adverse events (AE) throughout every stage of the clinical trial. The investigator should evaluate all adverse events according to the criteria and steps mentioned below.

11.2.1 Assessment of Intensity

Any adverse event has to be graded regarding its intensity.	
**MILD**	Does not interfere with subject's usual function, easily tolerated.
**MODERATE**	Interferes to some extent with subject's usual function.
**SEVERE**	Interferes significantly with subject's usual function, incapacitating with inability to work or carry out usual activity.

11.2.2 Assessment of Seriousness

Determination of the seriousness of the adverse event according to the definitions for a serious adverse event (SAE) as given in section Definitions (see 11.1).

11.2.3 Assessment of Causality

Determination of the relationship of the adverse events to the medicinal product(s) being studied after having evaluated all accessible data according to the following classification:

Suspected:

The temporal relationship between the event and the administration of the IMP makes a **causal relationship possible, probable, or definite,** or other drugs, therapeutic interventions or underlying conditions do not provide a sufficient explanation for the observed event.

Not suspected:

The temporal relationship between the event and the administration of the IMP makes a **causal relationship unlikely or impossible (i.e. not related),** or other drugs, therapeutic interventions or underlying conditions provide a sufficient explanation for the observed event.

When the final causality assessment is unknown and it is **uncertain** whether or not the investigational product caused the event, then the event should be handled as an SAE **related** (suspected) to the investigational product for reporting purposes.

11.3 Criteria to be evaluated by the Sponsor Delegated Person (2nd assessment)

In addition to the first evaluation of an adverse event that is performed by the investigator, a second evaluation with respect to seriousness, causality and expectedness and a risk-benefit assessment is performed by the Sponsor Delegated Person or an authorized representative to process safety evaluation according to a four-eyes principle.

11.4 Documentation and Reporting of Adverse Events

Any AE has to be documented in the CRF on the respective Adverse Event Report Form.

Documentation and evaluation of each AE occurring between:

- starting with first study specific intervention up to 30 days after the subject has received the last dose of trial drug

11.5 Documentation and Reporting of Serious Adverse Events

Any SAE has to be reported immediately to Winicker Norimed.

Documentation and evaluation of each SAE occurring:

- starting with first study specific intervention up to 30 days after the subject has received the last dose of trial drug.

11.5.1 Initial Reporting of SAEs

Any SAE has to be documented on the respective Serious Adverse Event Report Form, provided by Winicker Norimed to all investigators. The investigator has to

Report the SAE to WINICKER NORIMED GmbH

Deutschherrnstraße 15-19

90429 Nürnberg

Deutschland

IMMEDIATELY

after becoming aware of this event

(latest within 24 hours)

Fax: +49(0)911 – 926 80 4444

11.5.2 Reporting to the authorities and ethics committees

The SAEs of a clinical trial have to be notified to the competent authority once a year or on request.

Depending on the sort of the SUSAR the SDP has to consider special timelines for reporting to the competent authorities and ethics committees. The following timelines have to be considered for reporting of SUSARs to CA and EC:

Fatal or life-threatening SUSARs:

- as soon as possible, at the latest within 7 calendar days after first knowledge of the minimum criteria

Non-fatal and non-life-threatening SUSARs:

- as soon as possible, at the latest within 15 calendar days after first knowledge of the minimum criteria.

In multicenter trials the principal investigators of all other investigator sites will be informed about each SUSAR. All these tasks are delegated to Winicker Norimed by the SDP.

11.6 Pregnancy

Women of childbearing potential are required to have a negative pregnancy test to exclude a pregnancy before being randomized in the clinical trial.

Pregnancy testing will be conducted at the time points specified in the schedule of activities (see 2).

11.6.1 Actions to be taken if pregnancy occurs to female subjects or partners of male subjects

If a female subject becomes pregnant or is suspected to be pregnant (including a positive pregnancy test regardless of age or disease state) while participating in this trial and taking the study drug, or within 30 days of the last dose of the study drug, the investigator has to be informed immediately about this event. The pregnant subject has to discontinue the treatment with the IMP permanently, has to be excluded from the trial and has to be instructed to return any unused portion of the study drug to the investigator, if applicable. Likewise, if the partner of a male trial subject becomes pregnant or is suspected to be pregnant while the subject participates in this trial, the investigator has to be informed immediately about this suspected or confirmed pregnancy. The investigator will then provide this information to the sponsor /sponsor-delegated person for follow-up as necessary.

To ensure the safety of female subjects or female partners of male subjects, each pregnancy that becomes known to the investigator during the trial must be reported as an event. Therefore the investigator will record and report pregnancy information on the appropriate report form as an initial report and fax it immediately (at the latest within 24h) to WINICKER NORIMED GmbH

Fax: +49(0)911 – 926 80 4444)

The pregnancy itself is not considered to be an AE or SAE but must be followed up until delivery or until pregnancy termination and the outcome of pregnancy should be notified to the sponsor-delegated person to determine the outcome of the pregnancy regarding maternal or newborn complications. The investigator will seek and provide this follow-up information after the planned date of delivery. This information will be

forwarded to the sponsor-delegated person. For this purpose the pregnancy report form will be used as follow-up report. The timeframe for following-up the details of birth will be no longer than 28 days after the delivery date.

If the outcome of the pregnancy includes

- a spontaneous, therapeutic abortion or voluntary termination
- stillbirth
- neonatal death
- presence of birth defects or
- congenital anomaly (including that in an aborted fetus, stillbirth or neonatal death)

the investigator should report this outcome as an SAE.

All neonatal deaths that occur within 28 days of birth should be reported as SAEs without regard to causality. In addition, any infant death after 28 days that the investigator suspects is related to the in utero exposure to the study drug should be reported. Furthermore, any SAE occurring as a result of a post-trial pregnancy and considered reasonably related to the investigational medicinal product by the investigator will be reported as described in this section. The investigator is not obliged to actively seek this information in former trial participants, but has to meet the reporting obligations as soon as the investigator is aware of this event through spontaneous reporting by the person concerned.

12 Data Safety Monitoring Board (DSMB)

A Data Safety Monitoring Board (DSMB) will be established to review accumulating safety data during the course of the trial. The DSMB may recommend discontinuation of the trial or modification of the protocol for safety reasons at any time during the trial. The decision to terminate is taken by the sponsor delegated person together with the coordinating investigator. The members will be chosen from different fields. The members will not be involved in the design of the study, its conduct other than through their role on the DSMB and will have no financial interest in the outcome of the study. The DSMB will have written operating procedures.

13 Statistical Methods

13.1 Planned Statistical Analyses

Analysis of primary endpoint:

For the primary outcome change in SARA total score (DeltaSARA), the null hypothesis will be tested that the mean change is the same on placebo and on acetyl-DL-leucine. The significance level alpha is set to 5%.

The primary efficacy analysis will be performed according to the intention-to-treat (ITT) principle. Absolute change calculated as the difference between the period-level baseline score and the score measured at the 2-week visit and at the 6-week visit of the verum/ placebo period will be considered for a model-based primary efficacy analysis. If not revised in the *statistical analysis plan* (SAP), a linear mixed effects model for DeltaSARA will be performed (fixed effects: factors for treatment and visit, treatment-by-visit interaction, mean of both period-level baseline SARA total scores as covariate; normally distributed patient-specific random intercepts) in order to deal with repeated measurements within periods and measurements not made at equivalent times on each subject (due to unscheduled or missed clinic visits). This subject-specific modelling approach allows individual change scores over time to be calculated. To analyse the differences between both treatments at the end of the 6-week treatment period, 95% confidence intervals will be provided to quantitatively describe treatment effects.
Sensitivity analyses will be performed on a per protocol approach which accounts for compliance with medication according to the protocol. Additional efficacy analyses adjusting for genetically vs. sporadic CA, gender, age, or trial site, will be performed. Besides, the robustness of the overall result will be investigated by sensitivity analyses adjusting for speech-/physiotherapy (as considered appropriate during the blinded data review depending on the data quality). Further methodological details will be provided in the SAP.

Analysis of key secondary endpoints:

In case of a statistically significant primary efficacy result, confirmatory testing will be extended to the EQ-5D-5L (VAS and descriptive scale). Two-sided tests for detecting treatment differences will be carried out.

SCAFI (total score and 3 subscores), SARA subscale items, as well as patient-reported outcomes BDI-II and FSS are not considered in the hierarchical multiple testing procedures. Descriptive comparisons between treatment groups at the end of the 6-week verum/ placebo period will be performed by use of a two-sided Wilcoxon signed rank test, and additionally, Hodges-Lehmann estimation of the location shift between the two groups will be provided, as considered appropriate based on the blinded data. The same non-parametrical tests will be employed to estimate treatment differences at the follow-up visit 8 (close-out visit).

Safety analysis:

The frequency of adverse events and serious advents and the relationship to the study drug will be analyzed descriptively guided by MedDRA classification. Gender- as well as age-specific aspects will be given consideration by means of additional analyses if considered appropriate.

13.2 Interim Analysis

A fixed sample design is planned without confirmatory statistical testing for early decision making. There is no pre-planned efficacy interim analysis.

13.3 Sample Size Calculation

The sample size was calculated on the basis of the primary hypothesis, using own preliminary case series data (n=13 patients; [10]) and a similar placebo-controlled trial (N=20 patients; [31]) evaluating the efficacy of varenicline on the improvement in SARA total score in patients with SCA3.

Hence, assuming a minimum clinically relevant improvement in the SARA total score of 1.5 points [28] (the absolute change under verum is by 1.5 score points better as the change under placebo) and a standard deviation of the individual SARA change of 3.0 (which results in a standard deviation of 4.2 assuming an intra-patient correlation of 0.6), a **sample size of 86** (85 is calculated but 2 sequences are needed) **in total** is needed to have 90% power to detect a difference in means of 1.5, using a paired t-test with a 0.05 two-sided significance level [Software used: nQuery Advisor 7.0].

Assuming a dropout rate of about 20%, **a total of 108 patients have to be allocated to the trial**. With a proportion of about 50-55% recruited patients out of the number to be screened, 200 patients have to be assessed for eligibility.

13.4 Populations included in the Analysis

The primary endpoint of the clinical subject will be analysed primarily according to the intention-to-treat (ITT) principle. Amongst others, this means that, whenever possible, the subjects will be analysed according to the treatment sequence to which they were randomised, irrespective of whether they refused or discontinued the treatment or whether other protocol violations are revealed.

An analysis per-protocol (PP) for a specific endpoint excluded or censored endpoint information considering major protocol deviations potentially effecting subjects' specific endpoint value, e.g. in the case of major violation of eligibility criteria, lack of sufficient treatment per protocol, major use of prohibited concomitant therapy, or unsatisfactory examinations or evaluations for endpoint assessment. Exclusions or censoring with respect to endpoints confirmatory analysed after unblinding of the statistician will be listed and accounted for (e.g. incidentally finding of major violation of eligibility criteria during secondary analyses). The PP analyses will be performed for the purpose of a sensitivity analysis and investigating robustness of results.

13.5 Protocol Violations

Protocol violations are major deviations from the procedures outlined in this document, and may include

- the intake of concomitant medications not permitted during the trial
- missed evaluations
- non-compliance with the IMP (e.g. intake of trial drug < 1.5 g daily during treatment period (week 3-6), depending on the duration of intake per treatment period and drug counting)
- any non-adherence to the protocol that would have an impact to the subject's rights, safety or welfare.

Minor protocol deviations may include, but are not limited to, e.g.:

- intake of trial drug < 5 g daily and > 1.5 g daily during the treatment period (week 3-6), depending on the duration of intake per treatment period
- unscheduled visits

After a subject has been enrolled, it is the investigator's responsibility to make a reasonable effort to correct any protocol violations and to continue the subject's participation in the trial. Protocol violations do not justify withdrawal of a subject from the trial.

Protocol violations will be reported to the sponsor/sponsor delegated person during the course of the trial in the monitoring reports.

Protocol violations will be listed and the impact on the evaluation of the subjects concerned will be discussed and evaluated at regular time intervals (depending on the amount of protocol violations), and prior to unblinding for a statistical analysis. Handling of major deviations in statistical analyses will be fixed during a blinded data review before unblinding.

13.6 Handling of Dropouts, Withdrawal, and Missing Data

Subjects dropping out of the trial prior to randomization will be listed including the reason for dropout.

Subjects dropping out totally from the study or merely from the study assigned treatments after randomization will be analysed using all available data. No multiple imputation techniques will be performed for the primary efficacy analysis which is based on an "all observed data approach".

For secondary analyses, dropouts will be included, following ITT principles, by modern imputation techniques for missing data assuming ignorable missingness (e.g. by using the multiple imputations by chained equations (MICE)-approach). For per-protocol analyses, missings will not be imputed.

Sensitivity analyses will be performed to explore several testable and untestable assumptions including various scenarios of informative missingness, in order to assess the robustness of the overall trial result. A detailed description of the applied methods to investigate assumptions concerning missingness will be provided in the *statistical analysis plan*.

14 Data Collection, Handling and Record Keeping

Data management shall be performed by the Institute for Medical Informatics, Biometry and Epidemiology; LMU (IBE). All procedures have to follow Good Clinical Practice (GCP), existing standard operating procedures (SOP) and local laws and regulations as applicable.

14.1 Data Management

Details concerning data management procedures and query management will be described in a *data management* and *data validation plan*.

For the transfer of paper-based study data to the study database (eCRF if applicable) double data entry will be performed by two different persons. The two data entries will then be compared for data verification. An audit trail will be created to provide an electronic record of which data were entered or subsequently changed, by whom and when.

SAS software will be used to set up the trial (master and derived) database, and to review the data for completeness, consistency and plausibility. The checks to be programmed will be specified in the *data validation plan*. After running the check programs, the resulting queries will be sent to the investigator for clarification. All programs which can be used to influence the data or the data quality will be validated (e.g. check programs, programs used to import external data, etc.).

14.2 Data Coding

Coding of adverse events, medical history and concomitant diseases will be done according to the following coding systems:

- *MedDRA*

14.3 Documentation of Trial Data

14.3.1 Documentation of Trial Data in the Medical Record

The investigator will record the participation in the trial, the frequency of the trial visits, the relevant medical data, the concomitant treatment and the occurrence of adverse events in the medical record of each subject. The SARA and SCAFI scores will be documented by the investigator on source data worksheets that will be provided by the sponsor delegated person. The worksheets should be filed as source documents in the medical records of the respective subject.

The self-administered QoL questionnaires, BDI-II and FSS filled in by the patient will be filed as source data in the respective patient’s records. The original file will be provided to the data management for database entry.

Data collected on the CRFs must match the source data. These may include but are not limited to the hospitals' or the physician's medical files, laboratory and pharmacy records, diaries etc.

14.3.2 Case Report Form (CRF)

CRFs provided by the sponsor will be used to document the subject's data during the course of the trial. The data will be pseudonymized, processed and stored according to local law. This also allows preservation for future use.

The investigator has ultimate responsibility for the accuracy, authenticity, timely collection and reporting of all clinical, safety, laboratory data entered on the CRFs. All these data may only be entered and updated in the CRFs by authorized trial personnel.

In case of paper CRFs the completed pages will be sent to the data management for data entry and will be transferred to the sponsor delegated person at the end of the trial. A copy of each CRF page is archived at the investigator's site. The CRFs must be signed by the investigator or by an authorized staff member (physician) to confirm the correctness of the data documented on the CRF. Any corrections to entries made in the CRFs, source documents must be dated, signed and explained (if necessary) and should not obscure the original entry.

Changes are recorded (audit trail) and have to be annotated, once the respective CRF has been marked as completed.

After data cleaning (see section 14.1) and blinded data review, the data base will be locked and be used for unblinded statistical analysis.

Copies of the completed query forms will be stored per subject together with the CRF at the trial site.

14.4 Trial site File

The Trial site will be provided with a trial site file (ISF) containing all sponsor-specific essential and trial specific documents. The monitor will regularly check the trial site file for accuracy and completeness. The trial site file has to be stored locked and sure.

The ISF includes the subject identification list, where the investigator has to record the trial participation of each subject. This list allows identification of each subject and contains the subject number, the name, birth date and the date of inclusion (date of informed consent) of the subject into the trial, and will be reviewed by the monitor for completeness. After end of the trial the subject identification list remains with the study site (this information is not transferred to the sponsor). In addition, trial participation of the subject should be recorded in the Subject Screening and Randomization Log (where patients are pseudonymized, this can be transferred to the sponsor).

The investigator should maintain a list of appropriately qualified persons to whom he/she has delegated trial duties. This list (Team Delegation Log) will be provided with the ISF, too.

Furthermore, trial personnel responsible for documentation in the CRFs should be identifiable. Therefore a signature list with the name, signature, initials/abbreviation and trial responsibilities of all persons who are allowed to make entries into the CRF will be filed in the investigator's site file.

The trial documents provided by the sponsor are confidential and may not be made accessible to third parties not involved in the trial by the investigator or other staff members. All trial data are collected with subjects pseudonymized.

14.5 Archiving

In case of studies with marketed IMPs consider the requirements described below.

14.5.1 Sponsor

The sponsor must retain all essential documents inclusively the case report forms for the duration of at least 10 years after end or stop of trial. The sponsor must archive all trial related documents according to regulatory requirements.

14.5.2 Investigator

The investigator should maintain all subject documents as specified in Essential Documents for conduct of a clinical trial (see ICH-GCP, section 8) and as required by the applicable regulatory requirement(s) after completion of the clinical trial so that they will be available for audits and inspections by the authorities. The investigator will be responsible for the storage.

The following retention periods will apply after completion or stop of the clinical trial:

- all essential documents and trial related data must be retained securely for at least 10 years (GCP-V § 13 (10),
- the subject identification list for at least 15 years,
- medical records and other source documents for the longest possible period allowed by the hospital, the institution or the private practice.

The investigator/institution should take arrangements to prevent accidental or premature destruction and illegitimate access to these documents.

To enable evaluations and/or audits from regulatory authorities or the sponsor, the investigator agrees to keep records, including the identity of all participating subjects (sufficient information to link records, e. g. CRFs and hospital records), all original signed informed consent forms, copies of all CRFs, serious adverse event forms, source documents, and detailed records of treatment disposition, drug accountability and adequate documentation of relevant correspondence (e. g. letters, meeting minutes, telephone calls reports).

15 Reporting

15.1 Statistical Report

The statistical analyses and the statistical report are performed, evaluated and approved by Prof. Dr. Ulrich Mansmann, Institute for Medical Informatics, Biometry and Epidemiology. All data in this report are strictly confidential.

16 Definition of End of Trial

16.1 Regular End of the Trial

The regular end of the trial is defined as “Last Patient Last Visit“.

16.2 Termination of the Trial for Individual Subjects

If the clinical trial is prematurely terminated or suspended for any reason, the investigator should promptly inform the subjects and ensure appropriate therapy and follow-up.

Where required by the applicable regulatory requirements, the competent authority(ies) and the ethics committee(s) will also be informed.

There are different grades of deviations from the study flow ranging from minor, major protocol violations, (such as delayed or unscheduled study visits, discontinuation of taking the study drug, taking medication listed under prohibited therapy / concomitant medication (see protocol violations section 13.5) to complete withdrawal. Complete withdrawal should be a rare exception.

Whenever a subject is withdrawn totally from the trial, a complete final examination as scheduled for the premature termination/dropout visit should be conducted, and the circumstances of the withdrawal or discontinuation have to be recorded in detail in the medical file and the CRF.

If a subject does not return for a scheduled visit, every effort should be made to contact the subject (e.g. by *telephone visit* to assess and document treatment compliance, safety, or concomitant medication/ therapy) in order to avoid loss-to follow-up or dropout. These efforts have to be documented in the medical file.

Regardless of any decision to modify or discontinue their assigned intervention, study participants should be retained in the trial whenever possible to enable follow-up data collection and prevent missing data. In any circumstance, every effort should be made to document (key) subject outcomes, if possible. The subject has to be requested to return all unused investigational product(s), if applicable, and followed-up regarding any unresolved adverse events.

16.2.1 Termination by the Subject

Subjects may withdraw totally from the study trial at any time at their own request without stating the reason(s) for withdrawal. Nevertheless the investigator should inquire about the reason for withdrawal. They will experience no disadvantage as a result of this decision and no alternative therapy will be withheld by the investigator.

In this case the investigator is urged to ask the subject to return for a premature termination / dropout visit and to document information as much as possible in the CRF as defined in premature termination / dropout visit (see section 10.2.6).

Treatment discontinuation in treatment period 1: patient can be enrolled into treatment period 2 after a 4 week wash-out period. This would be classified as a minor protocol violation.

16.2.2 Termination by the Investigator

Subjects may also be withdrawn at any time at the discretion of the investigator for safety, behavioral, or administrative reasons, e.g.

- Occurrence of intolerable adverse events and which would constitute an unacceptable high risk for the subject
- Medically indicated e.g. because it is found that inclusion/ exclusion criteria were violated
- Continuation is unacceptable because risks outweigh the benefits
- Pregnancy
- Lack of compliance of the subject (e.g. taking prohibited medication)
- Significant protocol violations
- Logistical reasons (e.g. subject changes his/her doctor or hospital or moves to another location)

Whenever a subject is withdrawn from the trial, the reasons of the withdrawal or treatment discontinuation together with the date of last intake have to be recorded in detail in the patient’s medical records and the CRF. If a patient drops out totally from the study and no data will be collected after withdrawal, a complete final examination (dropout visit, see 10.2.6) should be conducted. In particular, every effort should be taken to assess the primary outcome. The subject has to be requested to return all unused investigational product(s), if applicable, and followed-up regarding any unresolved adverse events.

If a subject does not return for a scheduled visit, every effort should be made to contact the patient in order to regain him for further visits according to the protocol.

16.3 Termination of the Trial in Individual Sites

If the clinical trial is prematurely terminated or suspended for any reason, the investigator should promptly inform the subjects and ensure appropriate therapy and follow-up.

Where required by the applicable regulatory requirements, the competent authority(ies) and the ethics committee(s) will also be informed.

Both the investigator and the sponsor/sponsor delegated person have the right to terminate the trial at one of the centers at any time for instances:

- Unforeseeable circumstances have arisen at the trial center concerned what preclude the continuation of the clinical trial.
- The investigator considers that the resources for continuation are no longer available.
- The investigator considers that the continuation of the trial is no longer ethically or medically justifiable.
- Subject recruitment is inadequate.
- Serious problems arise with regard to the quality of the collected data which cannot be resolved.
- Withdrawal of the assenting opinion of the EC and/or regulatory authority.

Premature termination at one of the trial centers does not automatically mean a termination of already enrolled trial subjects. A separate decision on further treatment must be made for each subject, depending on the overall situation. So, it has to be clarified that:

An adequate further treatment and follow-up of already enrolled subject subjects must be ensured.

The documentation of already enrolled subject subjects will be reviewed for completeness and plausibility. Queries may be raised for further clarification before the center is closed. These queries must be answered properly by the center.

The competent authority(ies) and ethics committee(s) must be duly notified of the center's closure, including reasons, within the specified period(s).

The trial center concerned will be closed in stages by the CRA when a decision has been made on the further treatment of the subjects concerned.

17 Monitoring, Audits and Inspections

During the clinical trial, quality control and quality assurance will be endured through monitoring, auditing and inspections by authorities.

17.1 Monitoring

To ensure accurate, complete, consistent, and reliable data, the investigator's site(s) and trial procedures will be monitored by a representative of the sponsor. The sponsor's representative will visit the site:

- to evaluate the progress and recruitment of the trial,
- to review the source documents, CRFs and patient questionnaires for protocol compliance, data completeness, accuracy and validation,
- to assess facilities and equipment,
- to check for protocol compliance,
- to assure the AE/SAE reporting,
- to verify proper handling and dispensing of the IMP(s), and other factors.

The clinical monitor is also responsible for the transfer of the original CRFs to the sponsor. Frequency and scope of the monitoring visits will be defined in the *Monitoring Plan* for this trial which also includes the extent of source data verification that is required.

The investigator agrees to cooperate with the monitor to ensure that any problems detected in the course of these monitoring visits are addressed and resolved, and therefore ensures the accuracy and consistency of the trial with GCP and all applicable laws. The investigator allows the monitor to have access to all trial related original data and documents relevant for the monitoring of the trial.

17.2 Source Data Verification (SDV)

Source data verification will be performed in order to verify the accuracy and completeness of the entries on the case report form (CRF) by comparing them with the source data, and to ensure and increase the quality of the data. All data which are subject to SDV must have been entered in the medical record or, in the case of source documents, enclosed with the medical record. The investigators will afford the CRA access to the medical records for the performance of SDV.

Source data as defined by ICH-GCP include data such as hospital records, clinical and office charts, laboratory notes, memoranda, subjects' diaries or evaluation checklists, pharmacy dispensing records, recorded data from automated instruments, copies or transcriptions certified after verification as being

accurate copies, microfiches, photographic negatives, microfilm or magnetic media, x-rays, and records kept at the pharmacy, at the laboratories and at medico-technical departments involved in the clinical trial.

For each subject, a 100% SDV has to be performed at least for the following data:

- Year of birth
- Gender
- Ethnic origin, if applicable
- (Serious) adverse events (SAEs)
- Main inclusion/exclusion criteria
- Informed Consent
- Primary outcome

17.3 Audits and Inspections

In accordance with ICH GCP this trial may be selected for audit by representatives of the sponsor or for inspection by site responsible representatives of the local regulatory authority.

The investigator agrees to give the auditor access to all relevant documents for review and to support the sponsor to solve possible audit findings concerning the trial conduct at the respective site.

After every audit the auditee(s) will receive an audit certificate by the auditor. This document has to be filed together with the trial documentation and has to be made available also to the authorities in case of an inspection.

At the end of the trial, a copy of the audit certificate(s) will be included in the final report.

18 Ethics and Good Clinical Practice

The trial will be conducted in accordance with the ICH Guideline for Good Clinical Practice, the relevant national regulations and the Declaration of Helsinki.

18.1 Responsibilities of the Sponsor

The applicable national regulatory requirements will be complied with, according to the applicable legal requirements of the countries concerned.

According to German law (AMG §§ 40 – 42) the sponsor is responsible for obtaining the approval from the respective competent authority and the respective main research ethics committee before initiation of the trial. In addition the trial will be submitted to and approved by the appropriate independent research ethics committee for each participating center, prior to entering any subject into the trial.

According to German law (§ 4 Abs. 25 and § 40 Abs. 1 Nr. 5 AMG) the sponsor announces a Leader of the clinical trial (LKP) who has more than two years of experience in the field of clinical trial and holds a medical license.

In addition the trial has to be indicated to the local regulatory authority(ies) according to German law (§ 67 AMG) before initiation of the trial (inclusion of the first subject).

18.2 Responsibilities of the Investigator

By signing this protocol the local investigator declares his/her commitment:

- to not enrol any person dependent on him/her or the sponsor in accordance with the principles of ICH-GCP
- to follow the regulations for data security according to local legislation
- to inform the subjects of the transmission of their pseudonymized data according to documentation and transmission obligations and to make sure that subjects unwilling to give consent to the processing of their data are not included into the trial
- to certify that he/she was informed of the pharmacological – toxicological issues and risks of the clinical trial
- to be qualified by education, training and experience to assume responsibility for the proper conduct of the subject
- to be thoroughly familiar with the appropriate use of the trial drug(s), as described in the protocol, the product information and other information sources provided by the sponsor
- to be aware of, and comply with GCP and the applicable regulatory requirements

- to maintain a list of appropriately qualified persons to whom the investigator has delegated significant subject related duties (if applicable).

18.3 Ethics Committee and Competent Authority(ies)

The clinical trial protocol and amendments have to be approved by the Competent Authorities (CA), in addition to protocol and amendments the subject information and informed consent, and any other written information to be provided to the trial subjects have to be approved by the respective main research ethics committee (“federführende Ethikkommision” according to German law)) and by the appropriate independent research ethics committee for each participating site.

A copy of the written approval must be received by the sponsor before recruitment of subjects into the trial and shipment of trial drug.

Any substantial amendments to the protocol or subsequent changes to the informed consent form as a result of changes to the protocol must also be sent to the EC/CA. Records of the EC review and opinion of all documents pertaining to this trial must be kept on file by the investigator and are subject to regulatory authority and / or sponsor inspection during or after completion of the trial.

The *sponsor* will provide a safety update of the trial to the EC(s)/CA, including line listing, individual reports of SUSARs, if applicable, annually or more frequently if requested.

At the end of the trial, the sponsor will notify the EC(s)/CA about the trial completion. A copy of all reports submitted to the EC will be sent to the sponsor.

18.4 Compliance with the Protocol

The investigator should conduct the clinical trial in compliance with this protocol. For this purpose, the document will be signed by the sponsor and the investigator. As a general rule, the investigator should not deviate from the protocol or make amendments to the protocol without the agreement of the sponsor/authority/ethics committee (unless subject safety is at risk, see below).

Any deviations from the approved protocol should be documented and explained by the investigator or an individual who is designated by the investigator.

The investigator may deviate from the protocol or make an amendment to the protocol without prior approval of the ethics committee to eliminate immediate risks to the subject subjects. The deviation or amendment should subsequently be reported to the ethics committee, the sponsor or sponsor delegated person and, if necessary, the competent authority, giving reasons.

18.5 Notification of General Amendments to the Protocol

The sponsor can make general amendments to the protocol after the clinical trial has started. These may be of an administrative nature (logistical/administrative amendments) or substantial.

Substantial Amendments are changes that likely affect and /or change:

- the safety of the persons concerned,
- the interpretation of the scientific trial documents or the scientific informational value of the trial results,
- the nature of management or conduct of the clinical trial (e.g. change of coordinating investigator (German LKP), sponsor or sponsor's deputy),
- the pharmaceutical quality or safety of the investigational medicinal products
- the risk assessments concerning the health of persons who are not concerned, or the environment, in clinical trial with drugs consisting of or containing genetically modified organisms

Substantial Amendments require a new authorization of the Competent Authority and a new favourable opinion by the Ethics Committee.

The clinical trial may only be continued when a favourable opinion has been obtained from the competent ethics committee and if the competent authority has not raised any objections accompanied by reasons.

If applicable, an updated Informed Consent Form has to be signed by all subjects enrolled in the trial who are affected by the amendment.

Amendments which only have to be approved by the EC (e.g. changes in an advertisement for subjects to participate in the trial or changes in facilities for the trial, also will be notified to the CA with the comment “For information only”. Similarly, the EC will be informed of any substantial amendments for which only the CA is responsible (e.g. quality data).

If administrative protocol changes (e.g. change of monitoring, telephone numbers) are necessary, the EC and CA will be notified only.

18.6 Notification of the end of the trial

The end of the clinical trial is the date of the last visit of the last subject undergoing the trial.

If required by national law the CA(s) and EC(s) will be notified after the end of the trial in the country concerned or when the complete trial has ended.

Within one year of the end of the complete trial a summary of the trial report will be provided to the CA(s) and EC(s).

18.7 Annual Safety Report

Together with the notification of the end of the trial and once a year in case of long durations of the trial the sponsor/ sponsor delegated person will provide the CA(s) and EC(s) in the country concerned with a listing of all suspected serious adverse reactions which occurred over the trial period of the subject's safety. The annual safety report is provided by sponsor delegated person after AE reconciliation following the trial database.

18.8 Subject Information and Informed Consent

Every participating subject will be informed of nature, importance, treatment methods, risks and consequences of the trial by the local investigator. Details of indemnity and insurance are also stated.

The local investigator is responsible for obtaining written informed consent from a subject or legally acceptable representative before any protocol-specific screening procedures will be performed or any investigational products will be administered. The written informed consent document has to be prepared and provided in the language(s) of the potential subject population.

It is also the responsibility of the investigator for asking the subject if he/she agrees to have her primary care physician informed of his/her participation in the clinical trial. If the subject agrees to such notification, the investigator shall inform the primary care physician of the subject's participation by sending a message letter.

Subjects must understand that it is their own free will to participate and that they can withdraw consent at any time without giving reasons and without penalty or loss of benefits to which the subject is entitled. Also, subjects must understand that they will experience no disadvantage as a result of this decision and that no alternative therapy will be withheld by the investigator.

The subject will be given ample of time and opportunity to obtain answers to any open questions. All questions relating to the clinical trial should be answered to the satisfaction of the subject and/or his/her legal representative. On the other hand by signing the consent form subjects give their consent to the evaluation, recording and usage of their personal data.

The written consent form will be personally dated and signed by the subject and the by investigator conducting the informed consent discussion. The informed consent forms will be filed in the Trial site File at each site.

The acquisition of informed consent and the subject's agreement or refusal of the notification of the primary care physician should be documented in the subject's medical record.

A copy of the signed and dated informed consent form will be given to the subject or legally acceptable representative and a copy will be held in the subject's medical notes. The existence of written informed consent will have to be confirmed before any trial-specific test/treatment has been performed.

In the case of substantial amendments, e.g. any new data providing information on the safety profile of any of the investigational medicinal product and leading to significant changes in the risk-benefit ratio, the subject must be informed with an appropriately revised subject information and the consent of the subject has to be obtained again.

Changed trial procedures can only be carried out if they have been approved by the competent authority and the leading Ethics Committee, and if the subject has been appropriately informed and has given his/her written consent.

18.9 Subject Insurance

Every subject participating in the trial is insured against any trial-related illness/injuries pursuant to the legal requirements which may occur during the trial, in Germany according to § 40 Abs. 1 Nr. 8 and Abs. 3 AMG.

Excluded from this, however, are injuries to health and deterioration of illnesses already in existence which would have continued to exist even if the subject had not taken part in the clinical trial.

The investigator will inform the subject of the existence of the insurance, including the obligations arising from it. The subjects must be granted access to insurance documents and provided with a copy of the general conditions of insurance on request.

The insurance cover is jeopardized if the subject fails to immediately report to the investigator or responsible physician any injury to health which might have resulted from the participation in the clinical trial, or if she/he undergoes any other medical treatment (except for emergency treatment) without the investigator’s knowledge before her/his participation in the clinical trial has officially ended.

In case of any health impairment the subjector legally authorized representative is obliged to notify the insurance and additionally the investigator as soon as possible. The investigator is obliged to make a report to the sponsor.

The subject insurance will be arranged by the sponsor. The insurer will be:

Name of Insurer:	HDI-Gerling Industrieversicherung AG
Insurance Number:	39 130537 03026
Address:	Niederlassung München, Vertragsservice/M-B<br>Ganghoferstr. 39, D-80339 München
Phone:	+49 (0)89/2107-527
Fax:	+49 (0)89/9243-356

Organized by Ecclesia Mildenbrger Hospital GmbH

Klingenbergstrasse 4

32758 Detmold, Germany

Phone: +49/5231-603-6167

Fax: +49/5231-603-606167

E-Mail: daniela.schier@em-hospital.de

This insurance covers trial related injuries to health up to a maximum of 500.000 Euro per subject.

18.10 Data Protection and Subject Confidentality

The pertinent provisions of the country-specific legislation on data protection must be fully complied with.

The collection, transmission, archiving and evaluation of personal data in this clinical trial are performed according to local applicable laws (Data Protection Act). Prior to trial participation each subject must be informed by the investigator about the purpose and extent of the collection and use of personal data, particularly medical data, and must give written informed consent.

The subjects must be informed that:

Any subject related data in this trial are handled confidentially and will be captured in pseudonymized form (patient ID - year of birth) and will only be transmitted to

- the coordinating investigator/sponsor/sponsor delegated person/data monitoring safety board for scientific and adverse event evaluation
- the responsible regulatory authority(ies), the ECs of the trial sites and the European Data Base (EudraCT data base) for verifying the proper conduct of the trial and for assessment of trial results and adverse events

During monitoring, audits or inspections representatives of the sponsor (monitor, auditor) or of the local regulatory authority(ies) must have direct access to personal data. In this case, the investigator is released from confidential medical communication.

18.11 Financing of the Trial

The present trial is an investigator initiated trial (IIT).

The trial is funded by the Federal Ministry of Education and Research (Bundesministerium für Bildung und Forschung, BMBF) (grant code 01KG1422)

18.11.1 Trial Agreement / Investigator Compensation

According to ICH-GCP 4.9.6, a trial agreement on the conduct of the clinical trial and the compensation for conducting the subject will be signed between the sponsor (donor) of the clinical trial and the investigators

including their heads of administration (donee). A compensation will be paid for each fully documented, completed case.

18.11.2 Reimbursement of Subjects

Subjects will be compensated for their travel expenses in the context of trial site visits.

19 Trial Reports

After completion of the analysis by the responsible biostatistician, the final integrated medical and statistical report will be prepared and signed jointly with the biostatistician.

Except when required by law, no one will disclose a result of the clinical trial to third parties unless all parties involved have first agreed on the results of the analysis and their interpretation.

The final trial report will be written and signed in co-operation between the sponsor/coordinating investigator and the representatives of coordinating investigator.

20 Publication

20.1 Publication Policy

Data of the trial will be registered in a public data base (DRKS: http://www.germanctr.de; ISRCTN registry: http://www.isrctn.com). Efficacy and safety results will be submitted for at least one main publication in a peer-reviewed journal. Publication or lecture of data needs a previous annotation and approval of the Coordinating Investigator. All subject related data need to be published in a pseudonymous form.

The right of publication rests primarily with the sponsor, the coordinating investigator and the other investigators involved. All data collected in connection with the clinical trial will be treated in confidence by the sponsor/coordinating investigator and all others involved in the trial, until publication. Interim data and final results may only be published (orally or in writing) with the agreement of the sponsor. This is indispensable for a full exchange of information between the above-named parties, which will ensure that the opinions of all parties involved have been heard before publication. The agreement, which does not include any veto right or right of censorship for any of the parties involved, may not be refused without good reason.

Specific regulations concerning the publication policy in the applicable contracts will precede this trial protocol in any case.

21 References

1. Jacobi, H., et al., *The natural history of spinocerebellar ataxia type 1, 2, 3, and 6: a 2-year follow-up study.* Neurology, 2011. **77**(11): p. 1035-41.
2. Tsuji, S., et al., *Sporadic ataxias in Japan--a population-based epidemiological study.* Cerebellum, 2008. **7**(2): p. 189-97.
3. Erichsen, A.K., et al., *Prevalence of hereditary ataxia and spastic paraplegia in southeast Norway: a population-based study.* Brain, 2009. **132**(Pt 6): p. 1577-88.
4. Muzaimi, M.B., et al., *Population based study of late onset cerebellar ataxia in south east Wales.* J Neurol Neurosurg Psychiatry, 2004. **75**(8): p. 1129-34.
5. Ilg, W., et al., *Consensus Paper: Management of Degenerative Cerebellar Disorders.* Cerebellum, 2013.
6. Strupp, M., et al., *A randomized trial of 4-aminopyridine in EA2 and related familial episodic ataxias.* Neurology, 2011. **77**(3): p. 269-75.
7. Strupp, M., et al., *Treatment of episodic ataxia type 2 with the potassium channel blocker 4-aminopyridine.* Neurology, 2004. **62**(9): p. 1623-5.
8. Claassen, J., et al., *Dalfampridine in patients with downbeat nystagmus--an observational study.* J Neurol, 2013. **260**(8): p. 1992-6.
9. Ilg, W., et al., *Intensive coordinative training improves motor performance in degenerative cerebellar disease.* Neurology, 2009. **73**(22): p. 1823-30.
10. Strupp, M., et al., *Effects of acetyl-DL-leucine in patients with cerebellar ataxia: a case series.* J Neurol, 2013. **260**(10): p. 2556-61.
11. Ferber-Viart, C., C. Dubreuil, and P.P. Vidal, *Effects of acetyl-DL-leucine in vestibular patients: a clinical study following neurotomy and labyrinthectomy.* Audiol Neurootol, 2009. **14**(1): p. 17-25.
12. Highstein, S.M. and G.R. Holstein, *The anatomy of the vestibular nuclei.* Prog Brain Res, 2006. **151**: p. 157-203.
13. Vibert, N. and P.P. Vidal, *In vitro effects of acetyl-DL-leucine (tanganil) on central vestibular neurons and vestibulo-ocular networks of the guinea-pig.* Eur J Neurosci, 2001. **13**(4): p. 735-48.
14. Suh, B.C. and B. Hille, *PIP2 is a necessary cofactor for ion channel function: how and why?* Annu Rev Biophys, 2008. **37**: p. 175-95.
15. Witter, L., et al., *The cerebellar nuclei take center stage.* Cerebellum, 2011. **10**(4): p. 633-6.
16. Gunther, L., et al., *N-acetyl-L-leucine accelerates vestibular compensation after unilateral labyrinthectomy by action in the cerebellum and thalamus.* PLoS One, 2015. **10**(3): p. e0120891.
17. Sweatt, A.J., et al., *Branched-chain amino acids and neurotransmitter metabolism: expression of cytosolic branched-chain aminotransferase (BCATc) in the cerebellum and hippocampus.* J Comp Neurol, 2004. **477**(4): p. 360-70.
18. Hull, J., et al., *Distribution of the branched chain aminotransferase proteins in the human brain and their role in glutamate regulation.* J Neurochem, 2012. **123**(6): p. 997-1009.
19. Yennawar, N.H., et al., *Crystal structures of human mitochondrial branched chain aminotransferase reaction intermediates: ketimine and pyridoxamine phosphate forms.* Biochemistry, 2002. **41**(39): p. 11592-601.
20. Johnston, A.R., J.R. Seckl, and M.B. Dutia, *Role of the flocculus in mediating vestibular nucleus neuron plasticity during vestibular compensation in the rat.* J Physiol, 2002. **545**(Pt 3): p. 903-11.
21. Murai, N., et al., *Vestibular compensation in glutamate receptor delta-2 subunit knockout mice: dynamic property of vestibulo-ocular reflex.* Eur Arch Otorhinolaryngol, 2004. **261**(2): p. 82-6.
22. Aleisa, M., A.G. Zeitouni, and K.E. Cullen, *Vestibular compensation after unilateral labyrinthectomy: normal versus cerebellar dysfunctional mice.* J Otolaryngol, 2007. **36**(6): p. 315-21.

23. Klockgether, T., *Sporadic adult-onset ataxia of unknown etiology.* Handb Clin Neurol, 2012. **103**: p. 253-62.

24. Schmitz-Hubsch, T., et al., *Self-rated health status in spinocerebellar ataxia--results from a European multicenter study.* Mov Disord, 2010. **25**(5): p. 587-95.

25. Perlman, S.L., *Symptomatic and disease-modifying therapy for the progressive ataxias.* Neurologist, 2004. **10**(5): p. 275-89.

26. Lopez-Bastida, J., et al., *Social economic costs and health-related quality of life in patients with degenerative cerebellar ataxia in Spain.* Mov Disord, 2008. **23**(2): p. 212-7.

27. Subramony, S.H., *SARA--a new clinical scale for the assessment and rating of ataxia.* Nat Clin Pract Neurol, 2007. **3**(3): p. 136-7.

28. Schmitz-Hubsch, T., et al., *Scale for the assessment and rating of ataxia: development of a new clinical scale.* Neurology, 2006. **66**(11): p. 1717-20.

29. Schmitz-Hubsch, T., et al., *SCA Functional Index: a useful compound performance measure for spinocerebellar ataxia.* Neurology, 2008. **71**(7): p. 486-92.

30. Ristori, G., et al., *Riluzole in cerebellar ataxia: a randomized, double-blind, placebo-controlled pilot trial.* Neurology, 2010. **74**(10): p. 839-45.

31. Zesiewicz, T.A., et al., *A randomized trial of varenicline (Chantix) for the treatment of spinocerebellar ataxia type 3.* Neurology, 2012. **78**(8): p. 545-50.

32. Rabin, R. and F. de Charro, *EQ-5D: a measure of health status from the EuroQol Group.* Ann Med, 2001. **33**(5): p. 337-43.

33. Schmitz-Hubsch, T., et al., *Depression comorbidity in spinocerebellar ataxia.* Mov Disord, 2011. **26**(5): p. 870-6.

34. Brusse, E., et al., *Fatigue in spinocerebellar ataxia: patient self-assessment of an early and disabling symptom.* Neurology, 2011. **76**(11): p. 953-9.

22 Appendix

22.1 Ataxia score: SARA

Rater: ____________________________date: ______________patient:_______________________

Scale for the assessment and rating of ataxia (SARA)

**1) Gait**<br>Proband is asked (1) to walk at a safe distance parallel to a wall including a half-turn (turn around to face the opposite direction of gait) and (2) to walk in tandem (heels to toes) without support.<br>**0** **Normal, no difficulties in walking, turning and walking tandem (up to one misstep allowed)**<br>**1** **Slight difficulties, only visible when walking 10 consecutive steps in tandem**<br>**2** **Clearly abnormal, tandem walking >10 steps not possible**<br>**3** **Considerable staggering, difficulties in half-turn, but without support**<br>**4** **Marked staggering, intermittent support of the wall required**<br>**5** **Severe staggering, permanent support of one stick or light support by one arm required**<br>**6** **Walking > 10 m only with strong support (two special sticks or stroller or accompanying person)**<br>**7** **Walking < 10 m only with strong support (two special sticks or stroller or accompanying person)**<br>**8** **Unable to walk, even supported**	**2) Stance**<br>Proband is asked to stand (1) in natural position, (2) with feet together in parallel (big toes touching each other) and (3) in tandem (both feet on one line, no space between heel and toe). Proband does not wear shoes, eyes are open. For each condition, three trials are allowed. Best trial is rated.<br>**0** **Normal, able to stand in tandem for > 10 s**<br>**1** **Able to stand with feet together without sway, but not in tandem for > 10s**<br>**2** **Able to stand with feet together for > 10 s, but only with sway**<br>**3** **Able to stand for > 10 s without support in natural position, but not with feet together**<br>**4** **Able to stand for >10 s in natural position only with intermittent support**<br>**5** **Able to stand >10 s in natural position only with constant support of one arm**<br>**6** **Unable to stand for >10 s even with constant support of one arm**
**Score**	**Score**
**3) Sitting**<br>Proband is asked to sit on an examination bed without support of feet, eyes open and arms outstretched to the front.<br>**0** **Normal, no difficulties sitting >10 sec**<br>**1** **Slight difficulties, intermittent sway**<br>**2** **Constant sway, but able to sit > 10 s without support**<br>**3** **Able to sit for > 10 s only with intermittent support**<br>**4** **Unable to sit for >10 s without continuous support**	**4) Speech disturbance**<br>Speech is assessed during normal conversation.<br>**0** **Normal**<br>**1** **Suggestion of speech disturbance**<br>**2** **Impaired speech, but easy to understand**<br>**3** **Occasional words difficult to understand**<br>**4** **Many words difficult to understand**<br>**5** **Only single words understandable**<br>**6** **Speech unintelligible / anarthria**
**Score**	**Score**

Rater: ___________________________date: _____________patient:______________________

5) Finger chase

Rated separately for each side
Proband sits comfortably. If necessary, support of feet and trunk is allowed. Examiner sits in front of proband and performs 5 consecutive sudden and fast pointing movements in unpredictable directions in a frontal plane, at about 50 % of proband´s reach. Movements have an amplitude of 30 cm and a frequency of 1 movement every 2 s. Proband is asked to follow the movements with his index finger, as fast and precisely as possible. Average performance of last 3 movements is rated.

0 No dysmetria
1 Dysmetria, under/ overshooting target <5 cm
2 Dysmetria, under/ overshooting target < 15 cm
3 Dysmetria, under/ overshooting target > 15 cm
4 Unable to perform 5 pointing movements

Score	Right	Left
mean of both sides (R+L)/2		

6) Nose-finger test

Rated separately for each side
Proband sits comfortably. If necessary, support of feet and trunk is allowed. Proband is asked to point repeatedly with his index finger from his nose to examiner's finger which is in front of the proband at about 90 % of proband's reach. Movements are performed at moderate speed. Average performance of movements is rated according to the amplitude of the kinetic tremor.

0 No tremor
1 Tremor with an amplitude < 2 cm
2 Tremor with an amplitude < 5 cm
3 Tremor with an amplitude > 5 cm
4 Unable to perform 5 pointing movements

Score	Right	Left
mean of both sides (R+L)/2		

7) Fast alternating hand movements

Rated separately for each side
Proband sits comfortably. If necessary, support of feet and trunk is allowed. Proband is asked to perform 10 cycles of repetitive alternation of pro- and supinations of the hand on his/her thigh as fast and as precise as possible. Movement is demonstrated by examiner at a speed of approx. 10 cycles within 7 s. Exact times for movement execution have to be taken.

0 Normal, no irregularities (performs <10s)
1 Slightly irregular (performs <10s)
2 Clearly irregular, single movements difficult to distinguish or relevant interruptions, but performs <10s
3 Very irregular, single movements difficult to distinguish or relevant interruptions, performs >10s
4 Unable to complete 10 cycles

Score	Right	Left
mean of both sides (R+L)/2		

8) Heel-shin slide

Rated separately for each side
Proband lies on examination bed, without sight of his legs. Proband is asked to lift one leg, point with the heel to the opposite knee, slide down along the shin to the ankle, and lay the leg back on the examination bed. The task is performed 3 times. Slide-down movements should be performed within 1 s. If proband slides down without contact to shin in all three trials, rate 4.

0 Normal
1 Slightly abnormal, contact to shin maintained
2 Clearly abnormal, goes off shin up to 3 times during 3 cycles
3 Severely abnormal, goes off shin 4 or more times during 3 cycles
4 Unable to perform the task

Score	Right	Left
mean of both sides (R+L) / 2		

2

22.2 Ataxia score: SCAFI

SCA Functional Index – Case Report Form

Rater (initials):___________________

Date of examination:______________

Proband-Pseudonym

____

Timed walking test: 8m walk (8MW)

☐ test not performed, reason:____

☐ proband unable to walk due to physical limitations

assistive device ☐ none ☐ one cane /crutches ☐ two cane /crutches ☐ wheeled walker ☐ orthesis

Did situations arise that necessitated repetition of a trial (e.g. proband fell, external interference during walking, examiner forgot to start/ reset stopwatch) ?

____

Other factors that might have affected performance ?

____

Times are only given for two successfully completed trials.

Trial 1 ☐ *(0.1 sec)* **8MW_T1**

Trial 2 ☐ *(0.1 sec)* **8MW_T2**

Timed dexterity test: 9-hole peg test (9HPT)

☐ test not performed,
reason:____

☐ proband unable to perform test due to physical limitations

Did situations arise that necessitated repetition of a trial (e.g. pegboard not sufficiently secured on the table, external interference, examiner forgot to start/ reset stopwatch/ turn pegboard) ?

____

Other factors that might have affected performance ?

____

Times are only given for two successfully completed trials for each hand

DOMINANT HAND — **Right** ☐ **Left** ☐

Trial 1 [ ] 9HPTD_T1 *(0.1 sec)* **Trial 2** [ ] 9HPTD_T2 *(0.1 sec)*

NON-DOMINANT HAND — **Right** ☐ **Left** ☐

Trial 1 [ ] 9HPTN_T1 *(0.1 sec)* **Trial 2** [ ] 9HPTN_T2 *(0.1 sec)*

Timed speech task: PATA rate

☐ PATA rate task not performed, reason:______________________________________

☐ Proband unable to perform PATA rate task

Did situations arise that necessitated repetition of a trial (e.g. proband coughing, external interference during testing, examiner forgot to start stopwatch/ tape) ?

____

Othe factors that might have affected performance ?

____

Counts are only given for two successfully completed trials.

Trial 1 [ ] PATA_T1 **Trial 2** [ ] PATA_T2

2

22.3 Questionnaire for quality of life: EQ-5D-5L

EQ-5D-5L

Gesundheitsfragebogen

Deutsche Version

(German version for Germany)

Germany (German) v.2 © 2010 EuroQol Group. EQ-5D™ is a trade mark of the EuroQol Group

Bitte kreuzen Sie unter jeder Überschrift DAS Kästchen an, das Ihre Gesundheit HEUTE am besten beschreibt.

BEWEGLICHKEIT / MOBILITÄT

Ich habe keine Probleme, herumzugehen ❑
Ich habe leichte Probleme, herumzugehen ❑
Ich habe mäßige Probleme, herumzugehen ❑
Ich habe große Probleme, herumzugehen ❑
Ich bin nicht in der Lage, herumzugehen ❑

FÜR SICH SELBST SORGEN

Ich habe keine Probleme, mich selbst zu waschen oder anzuziehen ❑
Ich habe leichte Probleme, mich selbst zu waschen oder anzuziehen ❑
Ich habe mäßige Probleme, mich selbst zu waschen oder anzuziehen ❑
Ich habe große Probleme, mich selbst zu waschen oder anzuziehen ❑
Ich bin nicht in der Lage, mich selbst zu waschen oder anzuziehen ❑

ALLTÄGLICHE TÄTIGKEITEN *(z. B. Arbeit, Studium, Hausarbeit, Familien- oder Freizeitaktivitäten)*

Ich habe keine Probleme, meinen alltäglichen Tätigkeiten nachzugehen ❑
Ich habe leichte Probleme, meinen alltäglichen Tätigkeiten nachzugehen ❑
Ich habe mäßige Probleme, meinen alltäglichen Tätigkeiten nachzugehen ❑
Ich habe große Probleme, meinen alltäglichen Tätigkeiten nachzugehen ❑
Ich bin nicht in der Lage, meinen alltäglichen Tätigkeiten nachzugehen ❑

SCHMERZEN / KÖRPERLICHE BESCHWERDEN

Ich habe keine Schmerzen oder Beschwerden ❑
Ich habe leichte Schmerzen oder Beschwerden ❑
Ich habe mäßige Schmerzen oder Beschwerden ❑
Ich habe starke Schmerzen oder Beschwerden ❑
Ich habe extreme Schmerzen oder Beschwerden ❑

ANGST / NIEDERGESCHLAGENHEIT

Ich bin nicht ängstlich oder deprimiert ❑
Ich bin ein wenig ängstlich oder deprimiert ❑
Ich bin mäßig ängstlich oder deprimiert ❑
Ich bin sehr ängstlich oder deprimiert ❑
Ich bin extrem ängstlich oder deprimiert ❑

Germany (German) v.2 © 2010 EuroQol Group. EQ-5D™ is a trade mark of the EuroQol Group

- Wir wollen herausfinden, wie gut oder schlecht Ihre Gesundheit HEUTE ist.
- Diese Skala ist mit Zahlen von 0 bis 100 versehen.
- 100 ist die beste Gesundheit, die Sie sich vorstellen können.
  0 (Null) ist die schlechteste Gesundheit, die Sie sich vorstellen können.
- Bitte kreuzen Sie den Punkt auf der Skala an, der Ihre Gesundheit HEUTE am besten beschreibt.
- Jetzt tragen Sie bitte die Zahl, die Sie auf der Skala angekreuzt haben, in das Kästchen unten ein.

IHRE GESUNDHEIT HEUTE = ☐

Beste Gesundheit, die Sie sich vorstellen können

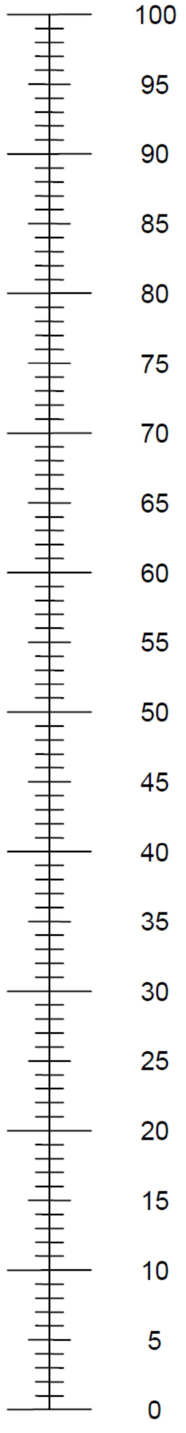

Schlechteste Gesundheit, die Sie sich vorstelle können

Germany (German) v.2 © 2010 EuroQol Group. EQ-5D™ is a trade mark of the EuroQol Group

22.4 BDI-II

BDI-II Fragebogen

Name	Alter	Geschlecht m / w	Datum

Anleitung: Dieser Fragebogen enthält 21 Gruppen von Aussagen. Bitte lesen Sie jede dieser Gruppen von Aussagen sorgfältig durch und suchen Sie sich dann in jeder Gruppe **eine Aussage** heraus, die am besten beschreibt, wie Sie sich **in den letzten zwei Wochen, einschließlich heute, gefühlt haben**. Kreuzen Sie die Zahl neben der Aussage an, die Sie sich herausgesucht haben (0, 1, 2 oder 3). Falls in einer Gruppe mehrere Aussagen gleichermaßen auf Sie zutreffen, kreuzen Sie die Aussage mit der höheren Zahl an. Achten Sie bitte darauf, dass Sie in jeder Gruppe nicht mehr als eine Aussage ankreuzen, das gilt auch für Gruppe 16 (Veränderungen der Schlafgewohnheiten) oder Gruppe 18 (Veränderungen des Appetits).

1.) Traurigkeit
- **0** Ich bin nicht traurig.
- **1** Ich bin oft traurig.
- **2** Ich bin ständig traurig.
- **3** Ich bin so traurig oder unglücklich, dass ich es nicht aushalte.

2.) Pessimismus
- **0** Ich sehe nicht mutlos in die Zukunft.
- **1** Ich sehe mutloser in die Zukunft als sonst.
- **2** Ich bin mutlos und erwarte nicht, dass meine Situation besser wird.
- **3** Ich glaube, dass meine Zukunft hoffnungslos ist und nur noch schlechter wird.

3.) Versagensgefühle
- **0** Ich fühle mich nicht als Versager.
- **1** Ich habe häufiger Versagensgefühle.
- **2** Wenn ich zurückblicke, sehe ich eine Menge Fehlschläge.
- **3** Ich habe das Gefühl, als Mensch ein völliger Versager zu sein.

4.) Verlust von Freude
- **0** Ich kann die Dinge genauso gut genießen wie früher.
- **1** Ich kann die Dinge nicht mehr so genießen wie früher.
- **2** Dinge, die mir früher Freude gemacht haben, kann ich kaum mehr genießen.
- **3** Dinge, die mir früher Freude gemacht haben, kann ich überhaupt nicht mehr genießen.

5.) Schuldgefühle
- **0** Ich habe keine besonderen Schuldgefühle.
- **1** Ich habe oft Schuldgefühle wegen Dingen, die ich getan habe oder hätte tun sollen.
- **2** Ich habe die meiste Zeit Schuldgefühle.
- **3** Ich habe ständig Schuldgefühle.

6.) Bestrafungsgefühle
- **0** Ich habe nicht das Gefühl, für etwas bestraft zu sein.
- **1** Ich habe das Gefühl, vielleicht bestraft zu werden.
- **2** Ich erwarte, bestraft zu werden.
- **3** Ich habe das Gefühl, bestraft zu sein.

7.) Selbstablehnung
- **0** Ich halte von mir genauso viel wie immer.
- **1** Ich habe Vertrauen in mich verloren.
- **2** Ich bin von mir enttäuscht.
- **3** Ich lehne mich völlig ab.

8.) Selbstvorwürfe
- **0** Ich kritisiere oder tadle mich nicht mehr als sonst.
- **1** Ich bin mir gegenüber kritischer als sonst.
- **2** Ich kritisiere mich für all meine Mängel.
- **3** Ich gebe mir die Schuld für alles Schlimme, was passiert.

9.) Selbstmordgedanken
- **0** Ich denke nicht daran, mir etwas anzutun.
- **1** Ich denke manchmal an Selbstmord, aber ich würde es nicht tun.
- **2** Ich möchte mich am liebsten umbringen.
- **3** Ich würde mich umbringen, wenn ich die Gelegenheit dazu hätte.

10.) Weinen
- **0** Ich weine nicht öfter als früher.
- **1** Ich weine jetzt mehr als früher.
- **2** Ich weine beim geringsten Anlass.
- **3** Ich möchte gern weinen, aber ich kann nicht.

PEARSON PsychCorp

© 2010 Pearson Assessment & Information GmbH, Frankfurt/M.

Summe Seite 1:

Bitte wenden

11.) Unruhe
- **0** Ich bin nicht unruhiger als sonst.
- **1** Ich bin unruhiger als sonst.
- **2** Ich bin so unruhig, dass es mir schwerfällt, still zu sitzen.
- **3** Ich bin so unruhig, dass ich mich ständig bewegen oder etwas tun muss.

12.) Interessenverlust
- **0** Ich habe das Interesse an anderen Menschen oder an Tätigkeiten nicht verloren.
- **1** Ich habe weniger Interesse an anderen Menschen oder an Dingen als sonst.
- **2** Ich habe das Interesse an anderen Menschen oder Dingen zum größten Teil verloren.
- **3** Es fällt mir schwer, mich überhaupt für irgend etwas zu interessieren.

13.) Entschlussunfähigkeit
- **0** Ich bin so entschlussfreudig wie immer.
- **1** Es fällt mir schwerer als sonst, Entscheidungen zu treffen.
- **2** Es fällt mir sehr viel schwerer als sonst, Entscheidungen zu treffen.
- **3** Ich habe Mühe, überhaupt Entscheidungen zu treffen.

14.) Wertlosigkeit
- **0** Ich fühle mich nicht wertlos.
- **1** Ich halte mich für weniger wertvoll und nützlich als sonst.
- **2** Verglichen mit anderen Menschen fühle ich mich viel weniger wert.
- **3** Ich fühle mich völlig wertlos.

15.) Energieverlust
- **0** Ich habe so viel Energie wie immer.
- **1** Ich habe weniger Energie als sonst.
- **2** Ich habe so wenig Energie, dass ich kaum noch etwas schaffe.
- **3** Ich habe keine Energie mehr, um überhaupt noch etwas zu tun.

16.) Veränderungen der Schlafgewohnheiten
- **0** Meine Schlafgewohnheiten haben sich nicht verändert.
- **1a** Ich schlafe etwas mehr als sonst
- **1b** Ich schlafe etwas weniger als sonst.
- **2a** Ich schlafe viel mehr als sonst.
- **2b** Ich schlafe viel weniger als sonst.
- **3a** Ich schlafe fast den ganzen Tag.
- **3b** Ich wache 1-2 Stunden früher auf als gewöhnlich und kann dann nicht mehr einschlafen.

17.) Reizbarkeit
- **0** Ich bin nicht reizbarer als sonst.
- **1** Ich bin reizbarer als sonst.
- **2** Ich bin viel reizbarer als sonst.
- **3** Ich fühle mich dauernd gereizt.

18.) Veränderungen des Appetits
- **0** Mein Appetit hat sich nicht verändert.
- **1a** Mein Appetit ist etwas schlechter als sonst.
- **1b** Mein Appetit ist etwas größer als sonst.
- **2a** Mein Appetit ist viel schlechter als sonst.
- **2b** Mein Appetit ist viel größer als sonst.
- **3a** Ich habe überhaupt keinen Appetit.
- **3b** Ich habe ständig Heißhunger.

19.) Konzentrationsschwierigkeiten
- **0** Ich kann mich so gut konzentrieren wie immer.
- **1** Ich kann mich nicht mehr so gut konzentrieren wie sonst.
- **2** Es fällt mir schwer, mich längere Zeit auf irgend etwas zu konzentrieren.
- **3** Ich kann mich überhaupt nicht mehr konzentrieren.

20.) Ermüdung oder Erschöpfung
- **0** Ich fühle mich nicht müder oder erschöpfter als sonst.
- **1** Ich werde schneller müde oder erschöpft als sonst.
- **2** Für viele Dinge, die ich üblicherweise tue, bin ich zu müde oder erschöpft.
- **3** Ich bin so müde oder erschöpft, dass ich fast nichts mehr tun kann.

21.) Verlust an sexuellem Interesse
- **0** Mein Interesse an Sexualität hat sich in letzter Zeit nicht verändert.
- **1** Ich interessiere mich weniger für Sexualität als früher.
- **2** Ich interessiere mich jetzt viel weniger für Sexualität.
- **3** Ich habe das Interesse an Sexualität völlig verloren.

Summe Seite 2:	
Übertrag Seite 1:	Gesamt Seite 1+2:

22.5 FSS

Fatigue Severity Scale (FSS)

Erschöpft und müde ist jeder von uns manchmal, doch viele Patienten leiden neben ihrer Grunderkrankung unter einer krankhaften Müdigkeit: der sogenannten *Fatigue*.

Mithilfe dieses Fragebogens lässt sich feststellen, ob eine Fatigue-Symptomatik vorliegt.

Wir möchten Sie bitten, die folgenden neun Aussagen jeweils auf einer Skala von 1 bis 7 zu bewerten.

1 bedeutet **„die Aussage trifft nicht zu"**
7 bedeutet **„die Aussage trifft in vollem Umfang zu"**

trifft nicht zu — trifft voll zu

	1	2	3	4	5	6	7
Ich habe weniger Motivation, wenn ich erschöpft bin.	☐	☐	☐	☐	☐	☐	☐
Körperliche Betätigung führt zu mehr Erschöpfung.	☐	☐	☐	☐	☐	☐	☐
Ich bin schnell erschöpft.	☐	☐	☐	☐	☐	☐	☐
Die Erschöpfung beeinflusst meine körperliche Belastbarkeit.	☐	☐	☐	☐	☐	☐	☐
Die Erschöpfung verursacht Probleme für mich.	☐	☐	☐	☐	☐	☐	☐
Meine Erschöpfung behindert körperliche Betätigung.	☐	☐	☐	☐	☐	☐	☐
Die Erschöpfung behindert mich an der Ausführung bestimmter Aufgaben und Pflichten.	☐	☐	☐	☐	☐	☐	☐
Die Erschöpfung gehört zu den drei mich am meisten behindernden Beschwerden.	☐	☐	☐	☐	☐	☐	☐
Die Erschöpfung hat Einfluss auf meine Arbeit, meine Familie bzw. mein soziales Leben.	☐	☐	☐	☐	☐	☐	☐

Mittelwert: ______________________

Aus physioonline © Georg Thieme Verlag Stuttgart 2008 (Adrian Pfeffer, Assessment: Fatigue Severity Scale, Physiopraxis 10/08)
