# Supplementary material for: Safety and Efficacy of Acetyl-DL-Leucine in Certain Types of Cerebellar Ataxia: The ALCAT Randomized Clinical Crossover Trial
Source: JAMA Netw Open. 2021 Dec 14;4(12):e2135841. doi: 10.1001/jamanetworkopen.2021.35841 (PMC8672236; doi:10.1001/jamanetworkopen.2021.35841)
Supplement: Supplement 2. — eMethods. Supplemental Methods eFigure 1. Trial Design: Schedule of Trial Activities and Crossover Intervention Scheme eTable 1. Full List of Eligibility Criteria as Stated in the Trial Protocol eTable 2. Results of the Primary Analysis (Full Analysis Set [FAS]): Fixed Effects and Variance Components, Estimates of the Principal Model (MMRM) eAppendix 1. Per Protocol (PP) Analysis for Primary Outcome eAppendix 2. Supplementary Analysis (FAS): Binary Outcome Measure for Treatment Response eTable 3. Individual Patient Data Listings: Errors in Stratification During Randomization (Hereditary vs. Non-Hereditary/Unknown Etiology) eTable 4. Additional Baseline Data for ITT Population eTable 5. Adverse Events (AEs) by Outcome, Grade, Attribution, and Seriousness in the Safety Set eFigure 2. Missingness Plot for SARA Total Score (FAS Population) eReferences [file jamanetwopen-e2135841-s002.pdf]

Supplemental Online Content

Feil K, Adrion C, Boesch S, et al; ALCAT Study Group. Safety and efficacy of acetyl-DL-leucine in certain types of cerebellar ataxia: the ALCAT randomized clinical crossover trial. *JAMA Netw Open.* 2021;4(12):e2135841. doi:10.1001/jamanetworkopen.2021.35841

eMethods. Supplemental Methods

eFigure 1. Trial Design: Schedule of Trial Activities and Crossover Intervention Scheme

eTable 1. Full List of Eligibility Criteria as Stated in the Trial Protocol

eTable 2. Results of the Primary Analysis (Full Analysis Set [FAS]): Fixed Effects and Variance Components, Estimates of the Principal Model (MMRM)

eAppendix 1. Per Protocol (PP) Analysis for Primary Outcome

eAppendix 2. Supplementary Analysis (FAS): Binary Outcome Measure for Treatment Response

eTable 3. Individual Patient Data Listings: Errors in Stratification During Randomization (Hereditary vs. Non-Hereditary/Unknown Etiology)

eTable 4. Additional Baseline Data for ITT Population

eTable 5. Adverse Events (AEs) by Outcome, Grade, Attribution, and Seriousness in the Safety Set

eFigure 2. Missingness Plot for SARA Total Score (FAS population)

eReferences

© 2021 Feil K et al. *JAMA Network Open.*

eMethods. Supplemental Methods

1. Trial participants

Patients' organizations have been included in the planning of the study, and a written declaration has been obtained with the Deutsche Heredo-Ataxie Gesellschaft (DHAG) Bundesverband e.V. Recruitment was via the center's own patients, referral from other neurologists, and by posting information on the website of the DHAG (https://ataxie.de/forschung).

Patients were not required to have a confirmed genotypic diagnosis and were not requested to undergo genetic testing to confirm underlying diagnosis prior to enrolment which increased the risk of misclassification during the stratified randomization.

2. Trial procedures

If within four weeks prior to screening visit (visit 1), a patient has received any of the medications for cerebellar ataxia as specified in the exclusion criteria (prohibited medication, e.g. 4-Aminopyridine retarded, acetyl-DL-leucine), irrespective of the preceding treatment duration, a run-in period of four weeks was required prior to enrolment. Otherwise, visits 1 and 2 coincided (**figure S1**).
Both the study examinations and treatment were performed in an outpatient setting.

This is a pragmatic trial. We aimed to assess the comparative effectiveness of the assigned prophylactic treatment regardless of additional concomitant therapies or symptomatic rescue medications, which can be denoted as a *'treatment policy strategy'* according to the ICH E9 addendum 2019.[1] Nevertheless, the doses of concomitant medications, e.g. for additional underling chronic conditions, or physio- or speech therapy due to cerebellar ataxia, had to be kept as constant as possible throughout the patient's observation period.

During the study, any adverse event (any untoward medical occurrence, including an abnormal laboratory finding, regardless of its causal relation to the study treatment) was recorded. The severity of the AE was graded as mild (minimal or no treatment required and no interference with the patient's daily activities); moderate (low level of inconvenience or concern, might need treatment and cause some interference with functioning); severe (patient's daily activities interrupted and systemic drug therapy or other treatment needed, usually incapacitating); and life-threatening (immediate risk of death).

3. Primary outcome: Scale for the Assessment and Rating of Ataxia (SARA)

The semi-quantitative rating scale of ataxia (SARA) specified as key inclusion criterion and measurement instrument to derive the primary efficacy outcome 'total score' is in the public domain and can be downloaded from
http://www.ataxia-study-group.net/html/about/ataxiascales/sara/SARA.pdf (accessed 20 Nov 2020).

High SARA scores indicate bad motor performance.

4. Secondary Efficacy Outcomes

In the following, outcome measurement instruments used to derive secondary efficacy outcomes are briefly described.

Composite SCA Functional index (SCAFI)
The Spinocerebellar ataxia functional index (SCAFI) is a quantitative composite performance measure and was generated as the arithmetic mean of all three Z-scores according to the *SCAFI Rating Manual* (available online at http://www.ataxia-study-group.net/html/about/ataxiascales/scafi/SCAFI.pdf; accessed 01 Nov 2020).

© 2021 Feil K et al. *JAMA Network Open.*

Z-scores standardize raw scores derived for the 3 subtests

- timed walking test (*8 meter walk* at maximum speed testing gait),
- timed dexterity test (*9-hole peg test* assessing limb ataxia),
- timed speech task (*"PATA"* repetition rate).

For each subtest/each hand in 9-hole peg test, the mean of trial1 and trial2 is calculated (e.g., $\text{PATA}_{\text{average}}$). Further, for each subtest, the individual raw score is converted into a Z-score standardizing the original velocity measures by the baseline values for the trial population (reference values). They result in a dimensionless quantity. E.g.,

$$\text{PATA Z-score} = (\text{PATA}_{\text{average}} - \text{baseline PATA}_{\text{average}} \text{ mean}) / \text{baseline SD PATA}_{\text{average}},$$

with SD = standard deviation.

Hence, the individual Z-scores can be expressed as SD higher (positive Z-score) or below (negative Z-score) the baseline mean of the population under study in each subtest.
The SCAFI is generated as the arithmetic mean of all three Z-scores. Increases in SCAFI score reflect improvement.

Beck's Depression Inventory (BDI-II)

We chose depression as representative measure of mental health problems. The severity of depression was assessed by the Beck Depression Inventory Second Edition (BDI-II).[2] The self-report BDI-II is widely used in depression trials and is used to measure the severity of depressive symptoms according the DSM-IV criteria.[3]
The BDI score is a sum score ranging from 0 to 63 for 21 items, each with a 4-point severity scale from 0 to 3; time frame: past 2 weeks including today; higher total scores on the BDI-II indicate more severe depressive symptoms.
Standardized cut-offs for total score values:
0 – 13 no or minimal, 14 – 19 mild, 20 – 28 moderate, ≥29 severe impairment.

Fatigue Severity Scale (FSS)

The FSS, a unidimensional fatigue scale, principally measures the impact of fatigue on specific types of functioning rather than the intensity of fatigue-related symptoms. The measurement instrument captures the patient's experience of mental or psychological fatigue and how it interferes with performing certain activities of daily living (exercise, work and family life). It is a self-reporting scale of 9 items (7-point Likert scale from 1 'strongly disagree' to 7 points 'strongly agree' per item); time frame: within the last week.

The *mean* of all answered items represents the fatigue severity ranging from 1 ('no signs of fatigue') to 7 ('most disabling fatigue'), whereas a high score value indicates high impairment. Due to its definition as a *mean* score handling incomplete questionnaires (i.e. missing items) can be done in a straightforward manner.

The mean (SD) FSS score for healthy individuals is 2.3 (0.7). A cut-off score of 4 or more is considered indicative of problematic fatigue.[4] A mean FSS value <5 means mild to moderate fatigue. A mean FSS value of 5 or higher indicates severe fatigue, the FSS value of 5 being the 95th percentile in healthy controls. [http://movingahead.psy.unsw.edu.au/documents/research/outcome%20measures/adult/TBI%20Related%20Symptoms/Website%20FSS.pdf; last accessed 20 Nov 2020].

Health related quality-of-life (EQ-5D-5L)

The EQ-5D-5L was applied as a self-report questionnaire of health status or health-related quality of life.
Time frame: today. Total questions: 6. Descriptive system: Mobility, Self-Care, Usual Activities, Pain/discomfort, Anxiety/depression; Health State by means of a Visual Analog Scale (VAS).
The VAS ranges from 0 to 100, with higher scores reflecting better perceived current health-related quality of life state.

For the description component, patients self-rate their health in terms of five dimensions; mobility, self-care, usual activities, pain/discomfort, and anxiety/depression using a five-level (EQ-5D-5L) scale for severity (no problems, slight problems, moderate problems, severe problems, extreme problems).
The patient's scores on these five dimensions were converted to a single utility index score using the EQ-5D-5L value set for Germany, which can be used in the clinical evaluation of health care. For the 10 patients enrolled at site Innsbruck, the value set for Germany was applied (no value set for Austria available).

© 2021 Feil K et al. *JAMA Network Open.*

5. Statistical analysis strategies

Two visits V1, V2 prior to enrolment
A 4 week run-in or screening period was required in the case the patient had to wash out medication related to the investigational medicinal product, with measurement of SARA score at V1 and at V2.
Due to regression-to-the mean, the second measurement assessed at V2 was considered as the true baseline visit for all statistical analyses (i.e. the measurement of motor function assessed at screening visit V1 was ignored).

Visits V1 and V2 coincided if a patient was not on prohibited medication for cerebellar ataxia four weeks prior to enrolment (**eFigure 1**).

Efficacy: Assumptions for the principal model (FAS; PP population)

The SARA total score rating ataxia-related symptoms was pre-specified as primary efficacy outcome.

A modelling approach was applied to deal with the longitudinal data structure for this crossover trial.[5-7]
A linear mixed-effects model was applied according to ITT, with the raw SARA total score as outcome measure assessed at each time points of both treatment periods.

Normally distributed patient-specific random intercepts were specified to account for patient-to-patient variation in symptom level at baseline visit. Missing at random was one of the key assumptions for the principal mixed model for repeated measures (MMRM) which avoids making any assumption about the response profile over time by treating time as a classification variable.[8] In particular, the evolution of the SARA total score was supposed to be nonlinear in the treatment period due to the underlying up-titration scheme.
To analyse the differences between both treatments at the end of the 6-week treatment period, 95% confidence intervals for target estimates and associated contrasts of primary interest were provided to quantitatively describe effects and to assess their clinical relevance.

Changes compared to the principal model pre-specified in the protocol (published version)
No mean of both period-dependent baseline SARA total scores was included as covariate in the principal model. Instead, pre-randomization/ period-dependent baseline score values (assessed at V2 and V5, respectively) were included in the model as additional time point.

Handling of missing values
Missing and unknown values in the SARA total score were not imputed due to the small number of missing values (see missingness plot in section 0). The same holds for symptom variables defined as secondary outcomes.

Subgroup analysis (hereditary vs. non-hereditary/unknown)
At the planning stage of the trial, we defined hereditary vs. non-hereditary/unknown as key subgroups.
Neither the hereditary nor the non-hereditary/unknown subgroup has demonstrated true drug versus placebo superiority with respect to the primary efficacy outcome SARA total score.
Neither subgroup has demonstrated true drug versus placebo superiority with respect to the secondary outcome SCAFI.

Safety: Analyses of treatment-emergent adverse events (TEAEs)

For some AEs, the exact starting date was partially missing (mainly day). In order to deal with this different input accuracy or partial date issues, AEs were classified with respect to their occurrence assuming the AE was experienced at the earliest possible date. By doing so, AEs were temporarily assigned to the placebo or acetyl-DL-leucine treatment period including a subsequent four week washout phase and post-treatment follow-up period, respectively (i.e. if occurring after the second treatment).

© 2021 Feil K et al. *JAMA Network Open.*

eFigure 1. Trial design. Schedule of trial activities and crossover intervention scheme.

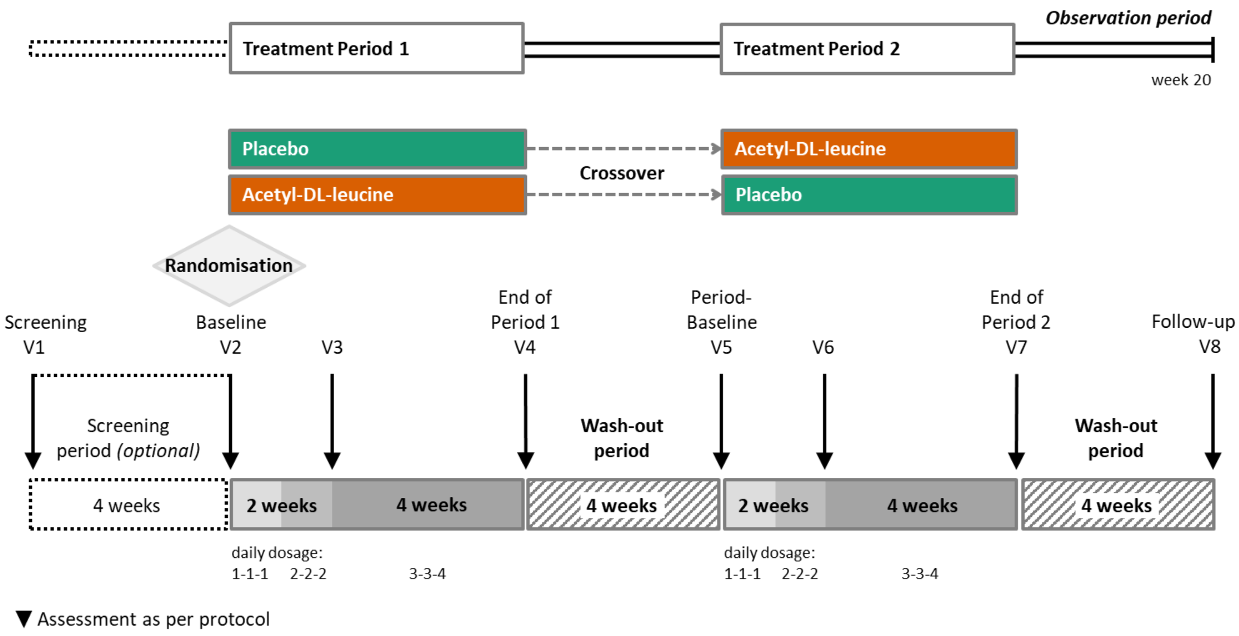

V = visit. V1 and V2 contemporaneous provided that the patient was not on prohibited medication for cerebellar ataxia four weeks prior to enrolment; otherwise, pre-treatment washout (run-in period) required. FU = follow-up. Three patients with hereditary cerebellar ataxia were excluded from the FAS after randomisation due to not fulfilling a major entry criterion: two were diagnosed as hereditary spastic paraplegia carriers (SPG7 gene), one patient with Friedreich's Ataxia.

© 2021 Feil K et al. *JAMA Network Open.*

eTable 1. Full list of eligibility criteria as stated in the trial protocol

**Inclusion Criteria**	Subjects will only be included in the study if they meet all of the following criteria:<br>▪ Clinically confirmed cerebellar ataxia (CA) with a total SARA score ≥3 (range 0−40) of hereditary or non-hereditary degenerative type<br>▪ Patient did not receive any of the following prohibited medication within 4 weeks prior to randomisation:<br>− aminopyridines (including sustained-release form)<br>− acetyl-DL-leucine<br>− riluzole<br>− gabapentin<br>− varenicline<br>− chlorzoxazone.<br>▪ The ability to follow study instructions and likely to attend and complete all required visits<br>▪ Written informed consent of the subject prior to any study specific intervention<br>▪ Age ≥18 years
**Exclusion Criteria**	Subjects will not be included in the study if any of the following criteria applies:<br>▪ Subject is not able to give consent<br>▪ Onset of ataxia in association with stroke, encephalitis, sepsis, hyperthermia or heat stroke<br>▪ Toxic causes for ataxia of cerebellar type<br>▪ Rapid progression of ataxia (development of severe ataxia in less than 12 weeks)<br>▪ Subject suffers from any of the following:<br>− chronic diarrhoea<br>− unexplained visual loss<br>− malignancies<br>− insulin-dependent diabetes mellitus<br>▪ Ataxia due to multiple sclerosis, ischemia, haemorrhage or tumour of the posterior fossa as confirmed by imaging<br>▪ Ataxia due to clinical likely multisystem atrophy type C (MSA-C) *<br>▪ Diagnosis of clinical likely Friedreich's ataxia (FRDA) **<br>▪ Known history of hypersensitivity to the investigational drug or derivates<br>▪ Liver failure defined as AST/ALT >300 U/l<br>▪ Simultaneous participation in another clinical trial or participation in any clinical trial involving administration of an investigational medical product within 30 days prior to the beginning of the clinical trial<br>▪ Subjects with a physical or psychiatric condition which at the investigator's discretion may put the subject at risk, may confound the trial results, or may interfere with the subject's participation in this clinical trial<br>▪ Known or persistent abuse of medication, drugs or alcohol<br>▪ Females of childbearing potential, who are not using and not willing to use medically reliable methods of contraception for the entire study duration as listed in the patient informed consent form

© 2021 Feil K et al. *JAMA Network Open.*

	▪ Current or planned pregnancy or nursing women<br>▪ Patients has received any of the following prohibited medication within 4 weeks prior to randomization<br>– aminopyridines (including sustained-release form)<br>– acetyl-DL-leucine<br>– riluzole<br>– gabapentin<br>– varenicline<br>– chlorzoxazone.<br><br>The indication specific exclusion criteria are chosen based on the available data on acetyl-DL-leucine of the French Agence nationale de sécurité du médicament et des produits de santé (http://agence-prd.ansm.sante.fr/php/ecodex/notice/N0126720.htm, dated on 20.02.2007), in particular hypersensitivity to the agent.

* MSA-C presents with heterogeneous symptoms and signs, including parkinsonian and autonomic symptoms. Furthermore, the course of the disease differs from other degenerative ataxias and is much faster, which could have hampered the observation of small but significant improvements. Therefore, they were not included.

** Patients with FRDA were not included because it is mainly caused by a degeneration of sensory neurons, dorsal root ganglia and the spinal cord leading to a mainly sensory ataxia. Based on the assumed mechanism of action of acetyl-DL-leucine primarily in the cerebellum we did not expect any benefit in patients with FRDA.

© 2021 Feil K et al. *JAMA Network Open.*

eTable 2. Results of the primary analysis (full analysis set (FAS), n=105): fixed effects and variance components for the principal model (MMRM)

Time points (visits) were denoted as follows:
t0 = pre-treatment or period-dependent baseline;
t1 = after 2-weeks on treatment; t2 = at the end of the 6-week treatment period (time point of primary interest).
ADL = acetyl-DL-leucine, PL = placebo.

	**SARA total score**			
	**Estimate**	**SE**	**95% CI**	**p value**
**Fixed effects**				
(Intercept)	13.470	0.547	(12.398 to 14.541)	<0.001
t0 (*period-dependent baseline)*	reference			
t1	-0.404	0.222	(-0.839 to 0.032)	0.069
t2	-0.515	0.223	(-0.952 to -0.078)	0.021
treatment: Placebo	reference			
treatment: ADL	-0.240	0.223	(-0.677 to 0.196)	0.281
period 1	reference			
period 2	-0.247	0.131	(-0.504 to 0.009)	0.059
t1 * ADL	0.431	0.314	(-0.184 to 1.046)	0.170
t2 * ADL	0.227	0.320	(-0.399 to 0.853)	0.478
**Random Effects**				
σ^2 *(residual variance)*	2.455			
τ_{patid} *(patient-specific intercept)*	28.382			
ICC	0.920			
N_{patid}	105			
Observations	590			
Marginal R^2 / Conditional R^2	0.002 / 0.921			

R-squared values are marginal and conditional R-squared statistics, based on Nakagawa et al. 2017.[9]
Parameters table drawn from the mixed model for repeated measures (MMRM) with SARA total score as response variable measured at three time points per period.
p value from MMRM model (fixed effects: factor variables for treatment (acetyl-DL-leucine vs. placebo), visit and treatment period, and treatment-by-visit interaction; random effects: patient-specific random intercepts).
FAS: Full analysis set; MMRM: mixed model for repeated measures; SE standard error of the parameter estimate; CI: Confidence interval; ICC: intra-class correlation coefficient.

Contrasts of interest concerning time (t0, t1, t2): estimated marginal means (least-squares (LS) means)

contrast	estimate	SE	df	lower CL	upper CL	t.ratio	p-value
t0 - t1	0.188	0.157	479.40	-0.181	0.557	1.199	0.455
t0 - t2	0.401	0.160	479.54	0.026	0.776	2.515	0.033
t1 - t2	0.213	0.160	479.20	-0.163	0.589	1.334	0.377

Results are averaged over the levels of: treatment, period.
SE = standard error. CL = confidence limit.

Contrasts of interest concerning treatment (acetyl-DL-leucine vs. placebo), by time t0, t1, t2: estimated marginal means

t0:

contrast	estimate	SE	df	lower CL	upper CL	t.ratio	p-value
PL vs ADL	0.240	0.223	480.09	-0.197	0.678	1.079	0.281

© 2021 Feil K et al. *JAMA Network Open.*

t1:

```
 contrast   estimate     SE      df   lower CL   upper CL   t.ratio   p-value
 PL vs ADL    -0.191  0.224  479.39     -0.630      0.248   -0.853      0.394
```

t2:

```
 contrast   estimate     SE      df   lower CL   upper CL   t.ratio   p-value
 PL vs ADL     0.013  0.230  479.35     -0.439      0.465    0.058      0.954
Results are averaged over the levels of: period
Degrees-of-freedom method: kenward-roger
```

The MMRM can also be used to compare differences in the mean absolute changes in SARA total score from period-dependent baseline (t0) to week 2 (t1) and week 6 (t2) between both treatment conditions.
The needed estimates derived from the principal model are given above.

**Changes in SARA total score from baseline to week 2 and 6**	**Estimated mean (95% CI)**		**Acetyl-DL-leucine – placebo**	**p value**
	**Acetyl-DL-leucine**	**Placebo**	**Mean difference (95% CI)**	
**Week 2**	0.027 (-0.407 to 0.462)	-0.404 (-0.839 to 0.032)	0.431 (-0.184 to 1.046)	0.170
**Week 6**	-0.288 (-0.736 to 0.160)	-0.515 (-0.952 to -0.078)	**0.227 (-0.399 to 0.853)**	0.478

[Results without considering period effects.]
Absolute change means post-treatment minus period-dependent baseline assessment. Week 6 represents the time point of primary interest.

© 2021 Feil K et al. *JAMA Network Open.*

eAppendix 1. Per protocol (PP) analysis for primary outcome

Mixed-model based analysis of primary outcome in the PP population (n=95 patients): Marginal means in SARA total score (raw values) for baseline, week 2, and week 6 representing the time point of primary interest; absolute change scores (baseline to week 2 and week 6).
(*For the definition of the PP set see Methods section (subsection Statistical Analysis) in the main article*.)

	**Estimated mean (95% CI)**		**Acetyl-DL-leucine – placebo**	**p value***
	**Acetyl-DL-leucine**	**Placebo**	**mean difference (95% CI)**	
**SARA total score**				
**Baseline † (t0)**	13.094 (11.972 to 14.216)	13.267 (12.145 to 14.389)	-0.173 (-0.624 to 0.278)	0.452
**Week 2 (t1)**	13.043 (11.922 to 14.165)	12.865 (11.743 to 13.987)	0.178 (-0.272 to 0.629)	0.437
**Week 6 (t2)**	12.754 (11.629 to 13.879)	12.801 (11.679 to 13.924)	**-0.047 (-0.509 to 0.414)**	0.840
**Changes in SARA total score from baseline to week 2 and 6**				
**Week 2**	-0.051 (-0.501 to 0.399)	-0.402 (-0.850 to 0.046)	0.352 (-0.285 to 0.988)	0.279
**Week 6**	-0.340 (-0.799 to 0.119)	-0.466 (-0.915 to -0.016)	**0.125 (-0.519 to 0.769)**	0.703

SARA = Scale for the assessment and rating of ataxia. t0 = pre-treatment or period-dependent baseline; t1 = after 2-weeks on treatment; t2 = at the end of the 6-week treatment period.

Estimated marginal means (LS means) derived from the mixed model for repeated measures (MMRM), averaged over the levels of period. Contrast of primary interest: difference is the effect of treatment (acetyl-DL-leucine vs placebo) on the efficacy outcome.
* p value from MMRM model (fixed effects: factor variables for treatment (acetyl-DL-leucine vs. placebo), visit and treatment period, and treatment-by-visit interaction; random effects: patient-specific random intercepts).

† Baseline means pre-treatment or period-dependent baseline.

Results of the primary analysis, PP set (n=95): fixed effects and variance components

	**SARA total score**			
	**Estimate**	**SE**	**95% CI**	**p value**
**Fixed effects**				
(Intercept)	13.389	0.570	(12.272 to 14.506)	<0.001
t0 (*period-dependent baseline)*	reference			
t1	-0.402	0.229	(-0.852 to 0.048)	0.080
t2	-0.466	0.230	(-0.917 to -0.014)	0.043
treatment: Placebo	reference			
treatment: ADL	-0.173	0.230	(-0.623 to 0.277)	0.452
period 1	reference			
period 2	-0.244	0.134	(-0.506 to 0.018)	0.068
t1 * ADL	0.352	0.325	(-0.285 to 0.988)	0.279
t2 * ADL	0.125	0.329	(-0.519 to 0.769)	0.703
**Random Effects**				
σ^2 *(residual variance)*	2.486			
τ_{patid} *(patient-specific intercept)*	27.939			
ICC	0.918			
N_{patid}	95			
Observations	560			
Marginal R^2 / Conditional R^2	0.002 / 0.918			

CI: Confidence interval; ICC: intra-class correlation coefficient.

© 2021 Feil K et al. *JAMA Network Open.*

eAppendix 2. Supplementary analysis (FAS): Binary outcome measure for treatment response

The absolute change from period-dependent baseline to week 6 in SARA total score was used to define a binary outcome measure for a post-hoc supplementary responder analysis (full analysis set).

Absolute change in SARA total score ≤ (-1.5) points

For this post-hoc supplementary analysis, a threshold of 1.5 score points was defined. Hence, treatment success means the SARA total scores improved by at least 1.5 points after 6 weeks.
success := absolute change ≤ (-1.5) score points after 6 weeks.

```
TRUE (“success”):      changescore <= (-1.5)
FALSE (“failure”):     changescore  > (-1.5)
```

whereas $changescore = TotalScore_{week6} - TotalScore_{period\text{-}dependent\ baseline}$

<u>Complete case analysis (ignoring missing values):</u>
(missing absolute change score values for period 1: 6 patients; for period 2: 16 patients)

```
       ADL
PL       FALSE TRUE <NA> Sum
  FALSE     36   22    5  63
  TRUE      16   11    0  27
  <NA>       6    7    2  15
  Sum       58   40    7 105

        Exact McNemar test (with central confidence intervals)
alternative hypothesis: true odds ratio is not equal to 1
odds ratio = 1.375 (95% CI 0.690 to 2.801; p=0.418)
```

<u>Worst score analysis, missing change scores set to treatment “failure”:</u>
To deal with missing values (e.g., missing second treatment period, no assessment at week 6 and/or second period-dependent baseline assessment; dropout before week 6 in the first treatment period), a missing absolute change score was set to failure (no matter if week 2 assessment was available or not). This is considered a ‘worst score analysis’ (single imputation method).

```
       ADL
PL       FALSE TRUE Sum
  FALSE     49   29  78
  TRUE      16   11  27
  Sum       65   40 105

        Exact McNemar test (with central confidence intervals)
alternative hypothesis: true odds ratio is not equal to 1
odds ratio = 1.813 (95% CI 0.952 to 3.573; p=0.072)
```

The probability for symptom relief (decrease in SARA total score of >1.5 points after 6 weeks) was not significantly higher on acetyl-DL-leucine compared to on placebo, odds ratio (OR) 1.813 (95% CI 0.952 to 3.573; p=0.072). The probability of treatment success increased by 81.3% (−4.8% to 257%) on acetyl-DL-leucine compared to on placebo; however, this was not statistically significant.
In a complete case analysis taking into account 85 completers (i.e. patients who completed both periods until week 6 and SARA measurements were available), the OR for treatment success on acetyl-DL-leucine compared to placebo was 1.375 (95% CI 0.690 to 2.801; p=0.418).

□

© 2021 Feil K et al. *JAMA Network Open.*

eTable 3. Individual patient listings: Errors in stratification during randomisation (hereditary vs. non-hereditary/unknown aetiology)

During the randomization process, 11/108 patients were misclassified due to uncertainties concerning cerebellar ataxia diagnosis at enrolment; 10 of these were part of the FAS sample (seven were incorrectly assigned to the stratum non-hereditary, three to the stratum hereditary).
The revised, detailed diagnosis for these patients was clinically re-assessed based on genetic testing while the patients were still on study treatment.

Due to this imbalance in errors in stratification, the intended treatment balance regarding the stratification factor was slightly disrupted.

No.	Site	FAS	PP	sequence group	allocated CA type at randomisation	CA type corrected *	Detailed CA diagnosis	CA type (coded)
1	Munich, DSGZ	**1**	**1**	P/A	non hereditary/ unknown	hereditary	SCA	other hereditary
2	Munich, DSGZ	**1**	**1**	P/A	non hereditary/ unknown	hereditary	SCA, dominant	ADCA (autosomal dominant)
3	Essen	**1**	**0**	P/A	non hereditary/ unknown	hereditary	SCA	other hereditary
4	Tuebingen	**1**	**1**	A/P	non hereditary/ unknown	hereditary	SCA, dominant	ADCA (autosomal dominant)
5	*Berlin, Charité*	***0***	***0***	*P/A*	*non hereditary/ unknown*	*hereditary*	*Friedreich's ataxia* †	*autosomal-recessive*
6	Innsbruck	**1**	**1**	A/P	non hereditary/ unknown	hereditary	SCA	other hereditary
7	Innsbruck	**1**	**1**	P/A	non hereditary/ unknown	hereditary	SCA	other hereditary
8	Innsbruck	**1**	**1**	P/A	non hereditary/ unknown	hereditary	SYNE1 ataxia	autosomal-recessive
9	Munich, DSGZ	**1**	**1**	P/A	hereditary	non hereditary	SAOA	sporadic
10	Munich, DSGZ	**1**	**0**	A/P	hereditary	non hereditary	CANVAS	sporadic
11	Munich, FBI	**1**	**1**	A/P	hereditary	non hereditary	CANVAS	sporadic

A/P (P/A) denotes treatment sequence acetyl-DL-leucine → placebo (or vice versa).

* CA diagnosis revised in the course of the trial (e.g. after diagnostic testing).

† One patient allocated to the P/A sequence group met the exclusion criterion Friedreich's Ataxia (diagnosis revised in the course of the trial leading to study dropout at the beginning of the second treatment period).

© 2021 Feil K et al. *JAMA Network Open.*

eTable 4. Additional baseline data for ITT population

Three composite subscales for SARA, three subtests for SCAFI.

Characteristics	Placebo followed by acetyl-DL-leucine (n=54)	Acetyl-DL-leucine followed by placebo (n=54)
**SARA composite subscales**		
SARA truncal ataxia (gait-posture subitems) *		
Mean (SD)	5.00 (3.18)	5.00 (3.66)
Median (range)	5.00 (1.00 – 15.00)	5.00 (1.00 – 16.00)
SARA speech disturbance †		
Mean (SD)	1.77 (0.94)	1.87 (1.19)
Median (range)	2.00 (0.00 – 4.00)	2.00 (0.00 – 4.00)
SARA limb ataxia ‡		
Mean (SD)	5.43 (2.37)	5.38 (2.38)
Median (range)	5.00 (1.50 – 10.50)	5.00 (0.50 – 13.50)
**SCAFI subtests** §		
8 meter walk test (8MWT) [sec]		
Mean (SD)	8.44 (4.41)	7.37 (3.43)
Median (range)	6.96 (4.30 – 23.85)	6.80 (4.25 – 22.80)
Missing, n (%)	5 (9.26)	7 (12.96)
9-hole peg test, dominated hand [sec.]		
Mean (SD)	41.01 (20.56)	43.09 (19.45)
Median (range)	35.17 (20.05 – 161.13)	38.05 (18.91 – 93.56)
Missing, n (%)	0	1 (1.85)
9-hole peg test, not dominated hand [sec.]		
Mean (SD)	46.20 (23.35)	44.38 (18.90)
Median (range)	39.35 (20.36 – 123.35)	38.75 (21.80 – 104.05)
Missing, n (%)	0	1 (1.85)
Timed speech task: PATA repetition rate [no. per 10 sec.]		
Mean (SD)	21.38 (6.11)	22.05 (6.15)
Median (range)	20.75 (10.00 – 38.50)	20.75 (11.50 – 36.50)
Missing, n (%)	0	0

Higher scores indicate greater impairment.

* This composite gait-posture subscore is the sum of item 1 (gait), 2 (stance) and 3 (sitting), resulting in a range of 0 to 18.
† Range 0 to 6.

‡ Limb kinetic function assessed by summarizing the composite subscores for item 5 to 8 (finger chase; nose-finger test; fast alternating hand movements; heel-shin slide test), resulting in a range of 0 to 16.

§ SCAFI, Spinocerebellar ataxia functional index: mean of two trials for each subtest/each hand.

© 2021 Feil K et al. *JAMA Network Open.*

eTable 5. Adverse events (AEs) by outcome, grade, attribution, and seriousness in the safety set (86 patients with at least one AE).

246 AEs for 86 patients (group A/P: 42 patients; group P/A: 45 patients) were documented throughout the course of the trial. Of these, 8 AEs were assessed as serious (**eTable 5**). In the A/P sequence group, two SAEs (skin texture abnormal; lumbar vertebral fracture), both events with moderate severity, were reported while on acetyl-DL-leucine. Of these, one SUSAR (lumbar vertebral fracture) was observed for a patient diagnosed with non-hereditary cerebellar ataxia leading to treatment termination in period 1 after 19 days. In the P/A group, four SAEs (myocardial infarction (moderate; severe); ischemic stroke (moderate); active suicidal ideation (severe)) were observed on acetyl-DL-leucine, two SAEs (thrombocytopenia (mild); transient global amnesia (moderate)) on placebo or the subsequent washout phase.
Overall, patients experienced a median of 2 AEs throughout their observational period. On acetyl-DL-leucine, 49.0% (51 out of 104) of the randomised participants reported no AEs, 36.5% (38) of participants reported one or two AEs throughout their observation period. On placebo, the corresponding numbers were: 47.6% (49 out of 103) patients no AEs, 44.7% (46) one or two AEs. In total, 191 (77.6%) AEs were of mild, 48 (19.5%) of moderate intensity, and 190 (77.2%) of AE outcomes were classified as recovered or resolved. The incidence of temporary discontinuation of drug intake or discontinuation of treatment due to AEs was only 4.9%; 4 patients discontinued due to an experienced AE on acetyl-DL-leucine, 8 on placebo. On acetyl-DL-leucine, 51 AEs were classified with a possible relationship to study treatment, compared to 41 on placebo (**eTable 5**).

The mean (SD) time from randomization to the occurrence of the patient’s first AE was 14.1 (17.6) days on acetyl-DL-leucine compared to 20.4 (18.8) days on placebo.

Throughout the course of the trial, 3 cases of unblinding of SAEs occurred:

- SAE: Preferred Term: myocardial infarction
- SAE: Preferred Term: idiopathic thrombocytopenic purpura
- SUSAR: Preferred Term: lumbar vertebral fracture.

In all three cases, study sites were not informed about the treatment code after unblinding of the coordinating investigator. Trial investigators and patients remained blinded. In the case of related SAEs and unexpected SAEs unblinding by means of emergency envelopes was done by the head of the clinical study centre not involved in outcome assessment.

© 2021 Feil K et al. *JAMA Network Open.*

Safety Assessment	Acetyl-DL-leucine (n=125 AEs)	Placebo (n=121 AEs)
**By outcome**		
Recovered	94 (49 [45%])	96 (49 [45%])
Recovered with sequelae	1 (1 [1%])	0
Not recovered	27 (16 [15%])	23 (16 [15%])
Deaths	0	0
Missing or unknown	3 (3 [3%])	2 (2 [2%])
**By grade**		
Mild	92 (43 [40%])	99 (48 [44%])
Moderate	29 (21 [19%])	19 (15 [14%])
Severe	3 (3 [3%])	3 (2 [2%])
Missing or unknown	1 (1 [1%])	0
**By attribution to study treatment** *		
Definite	0	0
Probable	3 (2 [2%])	9 (5 [5%])
Possible	51 (31 [29%])	41 (21 [19%])
Unlikely	44 (29 [27%])	48 (29 [27%])
Not related	26 (17 [16%])	23 (16 [15%])
Unknown	1 (1 [1%])	0
**By seriousness**		
Serious AEs	6 (6 [6%])	2 (2 [2%])
AEs but no serious AEs	118 (52[48%])	120 (59 [55%])
SUSARs	1 (1 [1%])	0
Discontinued because of (S)AEs †	4 (4 [4%])	8 (8 [7%])
**By System Organ Class** ‡		
General disorders and administration site conditions	17 (13.60); 13	16 (13.22); 15
Eye disorders	1 (0.80); 1	1 (0.83); 1
Respiratory, thoracic and mediastinal disorders	6 (4.80); 5	5 (4.13); 5
Skin and subcutaneous tissue disorders	7 (5.60); 5	2 (1.65); 2
Renal and urinary disorders	2 (1.60); 2	4 (3.31); 4
Blood and lymphatic system disorders	1 (0.80); 1	0
Gastrointestinal disorders §	20 (16.00); 16	24 (19.83); 19
Nervous system disorders	18 (14.40); 17	19 (15.70); 15
Ear and labyrinth disorders	3 (2.40); 3	0
Cardiac disorders	2 (1.60); 2	0
Infections and infestations	7 (5.60); 7	11 (9.09); 9
Psychiatric disorders	7 (5.60); 5	7 (5.79); 6
Musculoskeletal and connective tissue disorders	12 (9.60); 11	18 (14.88); 14
Metabolism and nutrition disorders	2 (1.60); 2	1 (0.83); 1
Injury, poisoning and procedural complications	20 (16.00); 14	13 (10.74); 9

Data are number of events (number of patients affected [%]), whereas percentages are based on the number of patients in the safety sample (n=108). For several patients, ≥ 1 AEs occurred (range 0 to 10 AEs per patient).
The safety set is identical to the full analysis set. In order to determine whether a single AE occurred during the 6-week treatment period on acetyl-DL-leucine or placebo, including the subsequent 4-week washout phase, the (estimated) start date of the AE was used for temporal assignment (appendix).

* Reasonable possibility for a causal relationship: drug-event relation as reported according to the adverse event case report form (CRF).

† AEs or SAEs leading to treatment discontinuation according to the adverse event CRF.

‡ AEs classified according to the Medical Dictionary for Regulatory Activities (MedDRA). System organ class (SOC) data are number of events (%); patients. For several patients, ≥ 1 AEs occurred (range 0 to 10 AEs per patient). For every treatment condition (acetyl-DL-leucine vs. placebo) a system organ class is counted only once for a single patient.

§ Nausea, vomiting, stomach pain, and diarrhoea reported most frequently

(S)AE = (serious) adverse event. SUSAR = Suspected unexpected serious adverse reactions.

© 2021 Feil K et al. *JAMA Network Open.*

eFigure 2. Missingness plot for SARA total score (FAS population).

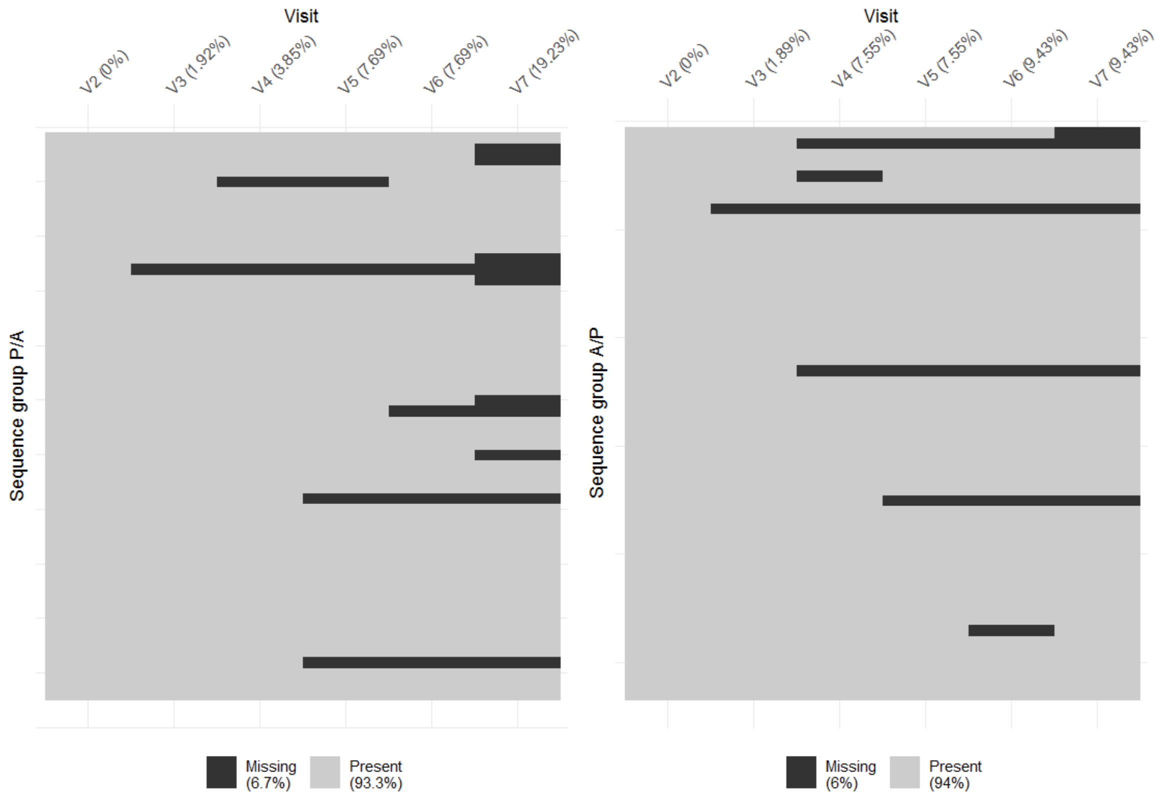

Missingness plot (stratified by sequence group) demonstrating the level of completeness over time concerning the primary outcome (FAS population).
Both figures display monotone and intermittent missing data patterns concerning SARA total score.
Light grey: data available; dark grey: missing data.
x-axis: visit number (definition according to figure S1).
y-axis: patients are sorted according to their original PatID within each sequence group.

© 2021 Feil K et al. *JAMA Network Open.*

eReferences

1. CHMP: **ICH E9 (R1) Addendum on estimands and sensitivity analysis in clinical trials to the guideline on statistical principles for clinical trials**. In: *EMA/CHMP/ICH/436221/201, Step 4 (Final Version adopted on 20 November 2019).* London, UK: European Medicines Agency, Committee for Medicinal Products for Human Use (CHMP); 2019.

2. Button KS, Kounali D, Thomas L, Wiles NJ, Peters TJ, Welton NJ, Ades AE, Lewis G: **Minimal clinically important difference on the Beck Depression Inventory--II according to the patient's perspective**. *Psychol Med* 2015, **45**(15):3269-3279.

3. American Psychiatric Association: **Diagnostic and statistical manual of mental disorders.**, 4th (DSM-IV) edn. Washington, DC: American Psychiatric Association; 1994.

4. Dittner AJ, Wessely SC, Brown RG: **The assessment of fatigue: a practical guide for clinicians and researchers**. *J Psychosom Res* 2004, **56**(2):157-170.

5. Jones B, Kenward MG: **Design and analysis of cross-over trials**, Third edn. Boca Raton, FL: Chapman & Hall / CRC Press; 2015.

6. Grieve AP: **A Note on the Analysis of the Two-Period Crossover Design When the Period-Treatment Interaction is Significant**. *Biom J* 1987, **29**(7):771-775.

7. Senn S: **Cross-over trials in clinical research**, vol. 5: John Wiley & Sons; 2002.

8. O'Kelly M, Ratitch B: **Clinical Trials with Missing Data: A Guide for Practitioners**: John Wiley & Sons; 2014.

9. Nakagawa S, Johnson PCD, Schielzeth H: **The coefficient of determination R^2 and intra-class correlation coefficient from generalized linear mixed-effects models revisited and expanded**. *Journal of The Royal Society Interface* 2017, **14**(134):20170213.

© 2021 Feil K et al. *JAMA Network Open.*
