# Supplementary material for: Safety and Efficacy of Acetyl-DL-Leucine in Certain Types of Cerebellar Ataxia: The ALCAT Randomized Clinical Crossover Trial
Source: JAMA Netw Open. 2021 Dec 14;4(12):e2135841. doi: 10.1001/jamanetworkopen.2021.35841 (PMC8672236; doi:10.1001/jamanetworkopen.2021.35841)
Supplement: Supplement 3. — Nonauthor Collaborators. ALCAT Study Group [file jamanetwopen-e2135841-s003.pdf]

*Indicates required information. Only first name, last name, and suffix will appear in PubMed.

*Group Name(s): ALCAT Study Group							
***First Name and Middle Initial(s)**	***Last Name**	***Suffix (eg, Jr, III)**	Academic Degrees	Institution	Location (city, state/province, country)	Role or Contribution, eg, chair, principal investigator	Group (if more than 1 Group listed in the byline) and/or Subgroup (eg, Steering Committee)
Annika	Spottke		MD	German Center for Neurodegenerative Diseases (DZNE), Center for Clinical Research, Bonn, Germany	Bonn, Germany	principal investigator	ALCAT
Ina	Vogt		MD	German Center for Neurodegenerative Diseases (DZNE), Center for Clinical Research, Bonn, Germany	Bonn, Germany	principal investigator	ALCAT
Jennifer	Faber		MD	German Center for Neurodegenerative Diseases (DZNE), Center for Clinical Research, Bonn, Germany	Bonn, Germany	principal investigator	ALCAT
Otmar	Bayer		MD	Department of Neurology with Friedrich-Baur-Institute, Ludwig Maximilians University, University Hospital, Munich, Germany and German Center for Vertigo and Balance Disorders (DSGZ), Ludwig Maximilians University, University Hospital, Munich, Germany	Munich, Germany	principal investigator	ALCAT
Nicolina	Goldschag		MD	Department of Neurology with Friedrich-Baur-Institute, Ludwig Maximilians University, University Hospital, Munich, Germany and German Center for Vertigo and Balance Disorders (DSGZ), Ludwig Maximilians University, University Hospital, Munich, Germany	Munich, Germany	principal investigator	ALCAT

Supplemental Online Content: Nonauthor Collaborators

*Indicates required information. Only first name, last name, and suffix will appear in PubMed.

***First Name and Middle Initial(s)**	***Last Name**	***Suffix (eg, Jr, III)**	Academic Degrees	Institution	Location (city, state/province, country)	Role or Contribution, eg, chair, principal investigator	Group (if more than 1 Group listed in the byline) and/or Subgroup (eg, Steering Committee)
Rachel-Maria	Zwergal		MD	Department of Neurology with Friedrich-Baur-Institute, Ludwig Maximilians University, University Hospital, Munich, Germany and German Center for Vertigo and Balance Disorders (DSGZ), Ludwig Maximilians University, University Hospital, Munich, Germany	Munich, Germany	principal investigator	ALCAT
Endy	Csanadi		MD	Department of Neurology with Friedrich-Baur-Institute, Ludwig Maximilians University, University Hospital, Munich, Germany and German Center for Vertigo and Balance Disorders (DSGZ), Ludwig Maximilians University, University Hospital, Munich, Germany	Munich, Germany	principal investigator	ALCAT
Ken	Möhwald		MD	Department of Neurology with Friedrich-Baur-Institute, Ludwig Maximilians University, University Hospital, Munich, Germany and German Center for Vertigo and Balance Disorders (DSGZ), Ludwig Maximilians University, University Hospital, Munich, Germany	Munich, Germany	principal investigator	ALCAT

Supplemental Online Content: Nonauthor Collaborators

*Indicates required information. Only first name, last name, and suffix will appear in PubMed.

***First Name and Middle Initial(s)**	***Last Name**	***Suffix (eg, Jr, III)**	Academic Degrees	Institution	Location (city, state/province, country)	Role or Contribution, eg, chair, principal investigator	Group (if more than 1 Group listed in the byline) and/or Subgroup (eg, Steering Committee)
Roman	Schniepp		MD	Department of Neurology with Friedrich-Baur-Institute, Ludwig Maximilians University, University Hospital, Munich, Germany and German Center for Vertigo and Balance Disorders (DSGZ), Ludwig Maximilians University, University Hospital, Munich, Germany	Munich, Germany	principal investigator	ALCAT
Christoph	Laub		MD	Department of Neurology with Friedrich-Baur-Institute, Ludwig Maximilians University, University Hospital, Munich, Germany and German Center for Vertigo and Balance Disorders (DSGZ), Ludwig Maximilians University, University Hospital, Munich, Germany and Department of Neurology, University of Augsburg, Augsburg, Germany	Munich, Germany	principal investigator	ALCAT
Olympia	Kremmyda		MD	Department of Neurology with Friedrich-Baur-Institute, Ludwig Maximilians University, University Hospital, Munich, Germany and German Center for Vertigo and Balance Disorders (DSGZ), Ludwig Maximilians University, University Hospital, Munich, Germany	Munich, Germany	principal investigator	ALCAT

Supplemental Online Content: Nonauthor Collaborators

*Indicates required information. Only first name, last name, and suffix will appear in PubMed.

***First Name and Middle Initial(s)**	***Last Name**	***Suffix (eg, Jr, III)**	Academic Degrees	Institution	Location (city, state/province, country)	Role or Contribution, eg, chair, principal investigator	Group (if more than 1 Group listed in the byline) and/or Subgroup (eg, Steering Committee)
Filipp	Filippopulos		MD	Department of Neurology with Friedrich-Baur-Institute, Ludwig Maximilians University, University Hospital, Munich, Germany and German Center for Vertigo and Balance Disorders (DSGZ), Ludwig Maximilians University, University Hospital, Munich, Germany	Munich, Germany	principal investigator	ALCAT
Anna Mira	Loesch-Biffar		MD	Department of Neurology with Friedrich-Baur-Institute, Ludwig Maximilians University, University Hospital, Munich, Germany and German Center for Vertigo and Balance Disorders (DSGZ), Ludwig Maximilians University, University Hospital, Munich, Germany	Munich, Germany	principal investigator	ALCAT
Josef	Herker		Dipl-Phys	Institute for Medical Informatics, Biometry and Epidemiology (IBE), Ludwig Maximilians University, Munich, Germany	Munich, Germany	data cleaning	ALCAT
Christiane	Neuhofer		MD	Department of Neurology with Friedrich-Baur-Institute, Ludwig Maximilians University, University Hospital, Munich, Germany and German Center for Vertigo and Balance Disorders (DSGZ), Ludwig Maximilians University, University Hospital, Munich, Germany	Munich, Germany	principal investigator	ALCAT

*Indicates required information. Only first name, last name, and suffix will appear in PubMed.

*First Name and Middle Initial(s)	*Last Name	*Suffix (eg, Jr, III)	Academic Degrees	Institution	Location (city, state/province, country)	Role or Contribution, eg, chair, principal investigator	Group (if more than 1 Group listed in the byline) and/or Subgroup (eg, Steering Committee)
Hans-Christoph	Diener		MD	Department of Neurology and Center for Translational Neuro- and Behavioral Sciences, University Hospital Essen, Essen, Germany	Essen, Germany	principal investigator	ALCAT
Ellen	Uslar		MD	Department of Neurology and Center for Translational Neuro- and Behavioral Sciences, University Hospital Essen, Essen, Germany	Essen, Germany	principal investigator	ALCAT
Jens	Claassen		MD	Department of Neurology and Center for Translational Neuro- and Behavioral Sciences, University Hospital Essen, Essen, Germany	Essen, Germany	principal investigator	ALCAT
Matthis	Synofzik		MD	Department of Neurology and Hertie-Institute for Clinical Brain Research, University Hospital Tübingen, Tübingen, Germany	Tübingen, Germany	principal investigator	ALCAT
Elisabetta	Indelicato		MD, PhD	Department of Neurology, Medical University Innsbruck, Innsbruck, Austria	Innsbruck, Austria	principal investigator	ALCAT
Andreas	Eigentler		MD, PhD	Department of Neurology, Medical University Innsbruck, Innsbruck, Austria	Innsbruck, Austria	principal investigator	ALCAT
Maria	Rönnefarth		MD	Department of Neurology, Charité – Universitätsmedizin Berlin, corporate member of Freie Universität Berlin, Humboldt-Universität zu Berlin, Berlin, Germany	Berlin, Germany	principal investigator	ALCAT
