# Supplementary material for: Safety and Efficacy of Acetyl-DL-Leucine in Certain Types of Cerebellar Ataxia: The ALCAT Randomized Clinical Crossover Trial
Source: JAMA Netw Open. 2021 Dec 14;4(12):e2135841. doi: 10.1001/jamanetworkopen.2021.35841 (PMC8672236; doi:10.1001/jamanetworkopen.2021.35841)
Supplement: Supplement 4. — Data Sharing Statement [file jamanetwopen-e2135841-s004.pdf]

Data Sharing Statement

Feil K, Adrion C, Boesch S, et al; ALCAT Study Group. Safety and efficacy of acetyl-DL-leucine in certain types of cerebellar ataxia (ALCAT): a randomized clinical crossover trial. *JAMA Netw Open.* 2021;4(11):e2135841. doi:10.1001/jamanetworkopen.2021.35841

Data

Data available: No

Additional Information

Explanation for why data not available: Data cannot be shared publicly because participants did not explicitly consent to the sharing of their data as per European Union's General Data Protection Regulation (EU GDPR) and the corresponding German privacy laws. The original study protocol, informed consent form, study data including de-identified participant data and an annotated case report form defining each field in the set will be made available to researchers upon formal request and receipt of a signed data sharing request form in accordance with the data sharing policies of the LMU Munich, Germany. Data are available through the Research Ethics Board of the LMU Munich for researchers who meet the criteria for access to confidential data and after approval of a research proposal. Requests for data access can be directed to ethikkommission@med.uni-muenchen.de and the Data Use and Access Committee (DUAC) of the Medical Faculty of the University of Munich (duac@med.uni-muenchen.de). Data will be made available for a minimum of 5 years after publication.
